# The therapeutic response of ER+/HER2− breast cancers differs according to the molecular Basal or Luminal subtype

**DOI:** 10.1038/s41523-020-0151-5

**Published:** 2020-03-06

**Authors:** François Bertucci, Pascal Finetti, Anthony Goncalves, Daniel Birnbaum

**Affiliations:** 1 Laboratoire d’Oncologie Prédictive, Centre de Recherche en Cancérologie de Marseille, Inserm U1068, CNRS UMR7258, Institut Paoli-Calmettes, Aix-Marseille Université, Marseille, France; 2 Département d’Oncologie Médicale, Institut Paoli-Calmettes, Aix-Marseille Université, Marseille, France

**Keywords:** Cancer therapeutic resistance, Breast cancer

## Abstract

The genomics-based molecular classifications aim at identifying more homogeneous classes than immunohistochemistry, associated with a more uniform clinical outcome. We conducted an in silico analysis on a meta-dataset including gene expression data from 5342 clinically defined ER+/HER2− breast cancers (BC) and DNA copy number/mutational and proteomic data. We show that the Basal (16%) versus Luminal (74%) subtypes as defined using the 80-gene signature differ in terms of response/vulnerability to systemic therapies of BC. The Basal subtype is associated with better chemosensitivity, lesser benefit from adjuvant hormone therapy, and likely better sensitivity to PARP inhibitors, platinum salts and immune therapy, and other targeted therapies under development such as FGFR inhibitors. The Luminal subtype displays potential better sensitivity to CDK4/6 inhibitors and vulnerability to targeted therapies such as PIK3CA, AR and Bcl-2 inhibitors. Expression profiles are very different, showing an intermediate position of the ER+/HER2− Basal subtype between the ER+/HER2− Luminal and ER− Basal subtypes, and let suggest a different cell-of-origin. Our data suggest that the ER+/HER2− Basal and Luminal subtypes should not be assimilated and treated as a homogeneous group.

## Introduction

Breast cancer (BC) is heterogeneous. The treatment and the patients’ inclusion in the clinical trials remain based upon clinicopathological features including immunohistochemistry (IHC), insufficient to capture the disease heterogeneity. The genomics-based classifications aim at identifying more homogeneous classes based on the functionality of molecular pathways and associated with a more uniform therapeutic response and outcome. Accumulating evidence suggests that these molecular subtypes provide clinically relevant information beyond clinicopathological classes^1–3^. In a recent study^4^, 13.1% of IHC estrogen receptor-positive HER2-negative (ER+/HER2−) BCs were reclassified as molecular Basal subtype by the 80-gene signature (80-GS)^5^. When compared to the ER+/HER2− cases reclassified as Luminal subtype (74.1%), the Basal samples displayed lower *ESR1* mRNA expression and increased relative ERΔ7 dominant-negative variant expression, shorter 3-year distant relapse-free interval (DRFI), and higher pathological complete response rate (pCR) to chemotherapy (CT). But the authors pointed to a few limitations: the limited number of ER+ Basal patients (54 for DRFI, 70 for pCR), the short median 34-months follow-up, and absence of information regarding the sensitivity to hormone therapy (HT). To reinforce these results and extend them to the response and/or potential vulnerability to HT and other systemic therapies of BC, and to assess the degree of difference between these subtypes, we analyzed in silico a meta-dataset including gene expression data from 8982 nonredundant BCs^6^, and DNA copy number/mutational and proteomic data from TCGA. Our aim was to compare the Basal versus Luminal samples.

## Results

### Prognostic analysis according to the molecular subtype

A total of 5836 samples were clinically defined as ER+/HER2−: 4341 (74%) were reclassified as Luminal by the 80-GS, 931 (16%) as Basal, and 564 (10%) as HER2-enriched. Because our aim was to compare the Luminal and Basal samples, the HER2-enriched samples were excluded, leaving 5272 samples for analysis. Regarding the prognostic features, the Basal samples comprised more grade 3 than the Luminal (*p* = 7.91E−22), more pT3 tumors (*p* = 3.25E−03), more *TP53*-mutated tumors (*p* = 2.77E−12), and more “high-risk” tumors according to prognostic gene expression signatures (GES): Mammaprint (*p* = 3.94E−56), Recurrence Score (*p* = 2.43E−121), and EndoPredict (*p* = 3.15E−73; Table 1). ER expression level was lower in the Basal samples than in the Luminal samples, in terms of both mRNA expression (*p* = 6.14E−193) and percentage of positive tumor cells by IHC (*p* = 4.33E−02; *p* = 2.33E−19; Fig. S1).

**Table 1:** Clinicopathological characteristics of patients and samples according to the molecular subtype.

Characteristics	*N*	ER+ Luminal	ER+ Basal	*p* value^a^	*N*	ER− Basal	*p* value^b^
Patients’ age				0.296			7.89E−26
≤50 years	1335	1115 (33%)	220 (31%)		632	632 (49%)	
>50 years	2795	2296 (67%)	499 (69%)		666	666 (51%)	
Pathological type				0.561			3.48E−16
Ductal	2241	1825 (74%)	416 (76%)		710	710 (85%)	
Lobular	394	324 (13%)	70 (13%)		21	21 (3%)	
Other	380	318 (13%)	62 (11%)		103	103 (12%)	
Pathological grade				7.91E−22			1.86E−251
1	637	562 (18%)	75 (11%)		15	15 (1%)	
2	1941	1660 (54%)	281 (42%)		160	160 (14%)	
3	1137	829 (27%)	308 (46%)		997	997 (85%)	
Pathological tumor size (pT)				3.25E−03			3.38E−10
pT1	1431	1207 (42%)	224 (38%)		288	288 (31%)	
pT2	1681	1398 (49%)	283 (49%)		542	542 (57%)	
pT3	324	248 (9%)	76 (13%)		114	114 (12%)	
Pathological axillary lymph node status (pN)				0.275			0.38
Negative	2168	1791 (58%)	377 (55%)		596	596 (59%)	
Positive	1626	1320 (42%)	306 (45%)		422	422 (41%)	
*TP53* mutation status				2.77E−12			6.02E−42
Wild-type	1559	1323 (95%)	236 (84%)		255	255 (71%)	
Mutated	108	63 (5%)	45 (16%)		103	103 (29%)	
Mammaprint relapse risk				3.94E−56			1.99E−231
Low	1879	1757 (40%)	122 (13%)		13	13 (1%)	
High	3393	2584 (60%)	809 (87%)		1647	1647 (99%)	
Recurrent score relapse risk				2.43E−121			<2.00E−255
Low	2110	1968 (45%)	142 (15%)		19	19 (1%)	
Intermediate	1575	1357 (31%)	218 (23%)		1555	86 (5%)	
High	1587	1016 (23%)	571 (61%)		86	1555 (94%)	
EndoPredict relapse risk				3.15E−73			<2.00E−255
Low	2729	2498 (58%)	231 (25%)		19	19 (1%)	
High	2543	1843 (42%)	700 (75%)		1641	1641 (99%)	
Pathological complete response (pCR)				3.72E−08			1.08E−15
No	468	410 (91%)	58 (68%)		271	271 (69%)	
Yes	69	42 (9%)	27 (32%)		123	123 (31%)	
Adjuvant HT				0.369			3.25E−83
No	1375	1134 (47%)	241 (49%)		655	655 (87%)	
Yes	1542	1292 (53%)	250 (51%)		101	101 (13%)	
Adjuvant CT				0.129			1.96E−45
No	3097	2598 (87%)	499 (84%)		756	756 (67%)	
Yes	496	402 (13%)	94 (16%)		367	367 (33%)	
5-year DRFI, % (95% CI)	2008	79% (77−82)	81% (77−86)	0.24	630	62% (58−67)	1.11E−15
DRFI event, yes	2008	362 (22%)	63 (18%)	0.168	630	201 (32%)	1.60E−07

*HT* hormone therapy, *CT* chemotherapy.

^a^*p* value for the comparison ER+ Basal versus ER+ Luminal.

^b^*p* value for the comparison between ER+ Basal, ER+ Luminal, and ER− Basal.

**Fig. 1:**
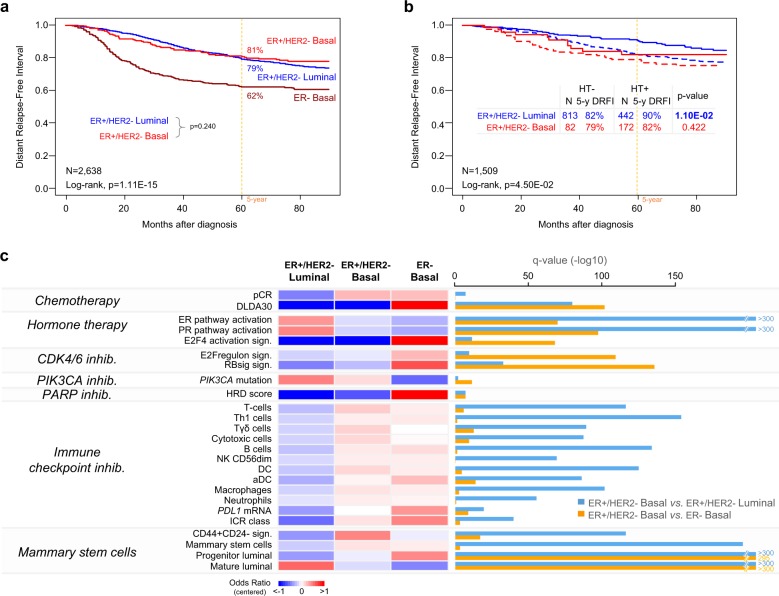
Comparison of the ER+/HER2− Basal subtype, ER+/HER2− Luminal subtype, and ER− Basal subtype breast cancers. **a** Kaplan−Meier postoperative DRFI curves in early BCs according to the ER IHC status and the 80-GS molecular type. **b** Similar to (**a**), but in ER+/HER2− early BCs patients only, untreated (dashed curves) and treated (solid curves) with adjuvant HT. **c** Heatmap of the odds ratios (ORs) of regression analysis between the three tumor subtypes (ER+/HER2− Luminal subtype, ER+/HER2− Basal subtype, and ER− Basal subtype used as reference for comparison) for different variables related to the percent of pCR after CT or the probability of therapeutic response of BC to CT, HT, CDK4/6 inhibitors, PIK3CA inhibitor, PARP inhibitors, and immune checkpoint inhibitors. Variables associated to mammary stem cells are also shown. For each variable, the ORs are mean-centered and color-coded according to the color scale shown below. On the right, the bar plots represent the log10-transformed *q* values of regression analysis for the comparison of each variable between ER+/HER2− Basal subtype versus ER+/HER2− Luminal subtype (blue bar), and between ER+/HER2− Basal subtype versus ER− Basal subtype (orange bar). The longer is the bar, the lower is the *q* value.

Regarding DRFI, 2008 patients operated for ER+/HER2− early BC were informative, including 1664 Luminal and 344 Basal. None of them had received any neoadjuvant systemic treatment, whereas 524 (35%) had received adjuvant HT and 342 (21%) adjuvant CT. With a median follow-up of 65 months (range, 1–299), the 5-year DRFI was not different between the two subtypes: 81% (95%CI 77–86) in Basal versus 79% (95%CI 77–82) in Luminal (*p* = 0.240; Fig. 1a). However, the temporal pattern of events differed with 65% of events (41/63) in the Basal within the first 3 years, versus only 44% (159/362) in the Luminal (*p* = 2.46E−03). In univariate analysis, the pathological grade and tumor size, and the use of adjuvant HT were associated with DRFI, whereas grade, axillary lymph node status and use of adjuvant HT were significant in multivariate analysis (Table S1).

### Therapeutic response/vulnerability according to the molecular subtype

Eighty-five Basal BCs and 452 Luminal BCs had received anthracycline-based neoadjuvant CT followed by surgery. We confirmed the higher chemosensitivity of Basal subtype with 32% (27/85) pCR rate versus 9% (42/452) in the Luminal subtype (*q* = 1.63E−06, Fig. 1c). Such difference was also predicted by the predictive DLDA30 GES^7^, which identified 30% (276/931) of Basal samples as “pCR-predicted” versus <1% (19/4341) of Luminal (*q* = 1.63E−78). Among the 1509 Luminal or Basal ER+/HER2− patients informative for DRFI and adjuvant HT, 524 and 985 had and had not received adjuvant HT, respectively. The Luminal subtype benefited from adjuvant HT with 90% (95%CI 87–94) 5-year DRFI with HT versus 82% (95%CI 79–85) without (*p* = 1.10E−02), whereas the Basal subtype did not (82% [95%CI 73–92] 5-year DRFI with versus 79% [95%CI 72–86] without; *p* = 0.422; Fig. 1b). This was confirmed in multivariate analyses (Table S2). In the Luminal subtype, the use of adjuvant HT was an independent favorable prognostic variable (*p* = 3.13E−02), whereas positive pN and higher grade were unfavorable variables. By contrast, in the Basal subtype, the use of adjuvant HT was not associated with DRFI (*p* = 0.952). Such lesser hormone sensitivity of the Basal subtype was also suggested (Fig. 1c) by the higher percentage of “high-risk” cases according to the E2F4-activation signature associated with resistance to HT^8^ (47% of Basal versus 34% of Luminal; *q* = 8.93E−11).

We then compared the potential vulnerabilities of ER+/HER2− Basal versus Luminal tumors to targeted therapies. We found higher RBsig score^9^ and E2F regulon score^10^ in the Basal subtype (*q* = 2.44E−31 and *q* = 1.02E−08 respectively), evocative of RB1-pathway disruption and associated with resistance to CDK4/6 inhibitors, suggesting a likely lower sensitivity to these drugs. By contrast, the percentage of patients with signature evocative of homologous recombination deficiency^11^ was higher in the Basal (21%: 25/109) versus Luminal samples (6%: 29/520; *q* = 5.18E−06), suggesting potential higher sensitivity to PARP inhibitors. The percentage of patients with *PIK3CA* hotspot mutation was higher in Luminal (37%: 571/1528) versus Basal (30%: 88/290), but the difference lost significance after correction for multiple testing (*q* = 0.100). Regarding the other actionable genetic alterations (AGAs) of BC with clinical evidence level equal to 1−2^12,13^, *FGFR2* and *KRAS* amplifications tended to more frequent and *PIK3CA* amplification more frequent in the Basal subtype (Table S3). Finally, the immune expression profiles were also very different. The Basal subtype displayed enrichment for the Bindea’s expression modules^14^ representing cell populations associated with adaptive and innate immunity (*q* < 1.00E−50), higher *PDL1* mRNA expression (*q* = 4.19E−23), and more frequent ICR4 class (24% versus 7%; *q* = 5.75E−39)^6^, suggesting potential for better response to immune checkpoint inhibitors.

### Molecular profiles of the ER+/HER2− Basal versus Luminal subtypes

We then compared the whole-exome mutational, whole-genome transcriptional, and proteomic (RPPA) profiles of the two subtypes in the TCGA set. Only one gene, *TP53*, was differentially mutated, in 39% of Basal versus 12% of Luminal samples (*q* = 2.17E−05: Table S4). We identified 906 genes as differentially expressed between the two ER+/HER2− subtypes (Table S5). The robustness of this gene list was confirmed by its capacity to differentiate the two subtypes in the independent validation set (Fig. S2). Ontology analysis (Table S6) showed that the genes upregulated in Basal samples were mainly involved in immune response. Comparison of RPPA profiles identified 89 proteins/phosphoproteins as differentially expressed between the two ER+/HER2− subtypes (Table S7). ER-alpha, PR and GATA3 were as expected among the top proteins overexpressed in the Luminal subtype, as well as androgen receptor (AR)^15^ and Bcl-2^16^, two therapeutic targets under development in ER+/HER2 BC, whereas PARP1 was among the top proteins overexpressed in the Basal samples.

To further compare the extent of differences between the ER+/HER2− Basal versus Luminal samples, we included the ER− Basal subtype. There was a gradient between the three molecular subtypes for nearly all comparisons (Table 1, Fig. 1c), the ER+/HER2− Basal subtype being intermediate between the two other ones, but much more different (as indicated by the *q* values in Fig. 1c) in terms of response or vulnerability to systemic therapies from the ER+HER2− Luminal subtype than from the ER− Basal subtype. Principal component analysis (PCA) of whole-genome transcriptional profiles (Fig. S3), and the classification of samples based on the 906 gene list (Fig. S2) confirmed that the ER+/HER2− Basal subtype was intermediate between the two other subtypes. Differences were also present with respect to the mammary stem cells signatures^17,18^, the ER+/HER2− Basal subtype being more related to the mammary stem cells, the ER+/HER2− Luminal to the mature luminal cells, and the ER− Basal to the progenitor luminal cells.

## Discussion

Our in silico analysis shows that the ER+/HER2− Basal subtype is very different from the ER+/HER2− Luminal subtype—and sometimes closer to the ER− Basal subtype—in terms of response and/or potential vulnerability to systemic therapies of BC. These results obtained on a large series reinforce the potential clinical value of the molecular subtypes within ER+/HER2− BCs, already suggested in smaller series^4,19^ regarding the prognosis after HT and the sensitivity to chemotherapy and CDK4/6 inhibitors. They also suggest differential therapeutic vulnerability regarding PARP inhibitors and platinum salts, PIK3CA inhibitor, immune therapy and other targeted therapies under development.

The Basal samples were more frequently associated with poor-prognosis features than the Luminal samples. However, the 5-year DRFI was not different, in agreement with the MINDACT trial^20^, but in contrast with the Groenendijk’s study likely because of a longer follow-up. Other studies have reported decreased survival outcomes in the Basal subtype when compared with the Luminal one in early BC treated with adjuvant tamoxifen^21^ and in advanced BC treated with letrozole^22^. In our study, the high percentage of ER+/HER2− patients untreated with adjuvant HT (65%) allowed to compare the benefit of adjuvant HT in the two subtypes, showing higher benefit in the Luminal subtype, even if the number of Basal patients was relatively small (*N* = 254) and precluded any definite conclusion. This high percentage, notably in the Luminal subtype (65% versus 32% in the Basal subtype), and this observation likely explain the absence of difference observed for DRFI between the two subtypes. Higher sensitivity to HT of the Luminal subtype might be at least in part related to higher ER expression level (mRNA and protein) and a more functional ER pathway (Gatza’s activation signatures). The Basal-type was more sensitive to neoadjuvant CT as previously reported in smaller series of ER+/HER2− patients^23,24^, likely in part because of higher pathological grade and cell proliferation rate (prognostic GES). Signatures predictive of response to CDK4/6 inhibitors, recently approved in BC^25^, suggested higher sensitivity of the Luminal subtype. In the PALOMA-2 study, the non-Luminal subtypes (20% of the entire population) had very small absolute benefits, if any, from palbociclib, whereas the Luminal subtype benefited substantially from palbociclib plus letrozole versus letrozole^26^. Our data provide new insights regarding the potential vulnerability to other drugs recently approved or in development in BC. For example, no data are available in the literature regarding the sensitivity to PARP inhibitors of the Luminal versus Basal subtypes within ER+/HER2− cases. Our results (HRD score) suggest that the drugs being evaluated in Basal/TN breast cancers (PARP inhibitors and platinum salts) deserve to be tested in the Basal subtype. No signature predictive for sensitivity exists for the alpelisib inhibitor^27^, but we found more frequent *PIK3CA* mutations, a clinically validated selection criterion for alpelisib, in the Luminal subtype, even if the difference was not significant after correction for multiple testing. A few level 2 AGAs, *FGFR2* and *KRAS* amplifications and *PIK3CA* amplification were more frequent in Basal samples. Finally, the Basal subtype displayed immune profile of “hotter” tumors than the Luminal subtype, suggesting potentially higher sensitivity to immune checkpoint inhibitors. Importantly, all these differences were not dependent on the molecular classification used because we observed similar results when using the PAM50 signature (Table S2, Fig. S4).

To our knowledge, we also report the first comparative analysis of large-scale molecular profiles of ER+/HER2− Basal versus Luminal subtypes. Only one gene (*TP53) was* differentially mutated, whereas important differences existed at the mRNA and protein levels. Five percent of genes tested were differentially expressed, including many immune genes upregulated in Basal samples. Thirty-nine percent of 226 proteins/phosphoproteins tested were differentially expressed, including therapeutic targets of drugs under development in ER+/HER2− BC such as PARP1, AR and Bcl-2. Inclusion of the ER− Basal subtype showed a gradient between the three subtypes from the ER+/HER2− Luminal subtype to the ER− Basal subtype in terms of clinicopathological correlations and transcriptional profiles. However, the ER+/HER2− Basal subtype was closer to the ER− Basal subtype than to the ER+/HER2− Luminal subtype in terms of response/probability of response to systemic therapies. The extent of differences between subtypes was also suggested by the mammary stem cell signatures, which could suggest a different cell-of-origin. Strikingly, the ER+/HER2− Basal samples were closer to the mammary stem cells than were the ER− Basal samples, warranting further investigations.

In conclusion, our results reinforce the potential clinical value of the different molecular classifications in ER+/HER2− BCs: the Basal and Luminal subtypes are so different with respect to therapeutic response/vulnerability, metastatic risk and cell-of-origin that they cannot continue to be assimilated and treated as a unique homogeneous ER+/HER2− group. Validation in prospective clinical trials is warranted, and caution is required in the interpretation of ongoing trials and the design of future trials.

## Methods

### Breast cancer samples, gene profiling and data analysis

We analyzed our BC gene expression database^6^ pooled from 36 public datasets (Table S8), comprising 8982 invasive primary BCs. The details of Institutional Review Board and Ethical Committee approval and patients’ consent for all 36 studies are present in their corresponding publications listed in Table S8. The preanalytic data processing was done as described^6^. We also collected DNA and proteomic data from TCGA (WES data, array-CGH, HRD score, RPPA)^11^ and the *PIK3CA* and *TP53* mutational statutes of TCGA and Metabric^28^.

ER and HER2 statutes were preferentially determined by study annotation when in agreement with the recent ASCO guidelines^29,30^. Where unavailable, the normalized gene expression data were used to infer the receptor status. Indeed, several points made impossible to apply the same definition of IHC positivity to all samples: the definition of ER-positivity by IHC was not available across all studies, and when available, the positivity cut-off was not similar between all studies (1% or 10%); the percentage of tumor cells stained was not available across most of studies; and the ER status was missing for a few samples. The cut-off value for ER status in the most recent ASCO guidelines is 1%^30^. Thus, we have kept the IHC status annotated in the original study when it was based upon a cut-off value of 1%. When it was annotated positive with a 10% cut-off, the sample remained of course positive with the 1% cut-off. When we could not redefine the ER status according to the ASCO guidelines (e.g. in case of a negative status when a 10% cut-off on IHC was used in the original study, or in case of unavailability), we inferred the ER status from transcriptional data of ESR1, by using a two-component Gaussian finite Mixture Model using maximum likelihood estimation on a per-study basis as previously described^31^. This was made possible thanks to the bimodal distribution of mRNA expression level. The mRNA threshold was verified on the 5548 samples of our database ER-annotated according to the 1% ASCO guideline IHC cut-off: the concordance rate was 93%, suggesting good performance. Out of the 8982 samples of our database, 6563 were defined as ER+ (5342 according to IHC and 1221 according to inferred stratus).

The same process was applied for defining the HER2 status, the cut-off of which was based on the recent ASCO guidelines^29^. In accordance with this guideline, the samples were considered HER2+ when they reached an IHC score of 3+ or 2+ with FISH amplification. A HER2 IHC score of +1 or 0 was considered negative. When the information was not available, and thanks to the bimodal distribution of mRNA expression level, the HER2 status was inferred from the mRNA expression level as described for ER^31^. The mRNA threshold was verified within the 6563 ER+ samples on the 3050 HER2-annotated according to the ASCO guidelines: the concordance rate was 91%, suggesting good performance. Out of the 6563 ER+ samples of our database, 727 were defined as HER2+ (421 according to IHC and 306 according to inferred stratus), leaving 5836 ER+/HER2− samples.

We applied to each dataset separately several GES: 80-GS^5^ and PAM50^32^, and the surrogate signatures of three commercial prognostic signatures (Recurrence Score^33^, Mammaprint^34^, EndoPredict^35^), Gatza’s ER and PR pathways activation signatures^36^, E2F4-activation signature associated with resistance to HT^8^, two scores associated with resistance to CDK4/6 inhibitors on preclinical models for RBsig^9^ and on clinical samples of PALOMA-3 trial for E2F regulon^10^, several Bindea’s expression modules^14^ representing different immune cell populations, the immunologic constant of rejection (ICR) signature^6^, and two signatures related to mammary epithelial cell hierarchy and stem cells^17,18^. For all above-quoted GES, all 5272 samples (931 Basal and 4341 Luminal) were informative. The *PDL1*/CD274 mRNA expression level was assessed as previously described^17,18^. The present in silico study was approved by our Institutional Review Board (No. 19-006; March 15, 2019).

Supervised analyses compared the profiles of ER+/HER2− Basal versus Luminal TCGA samples at several levels: WES mutational and RPPA proteomic using logistic regression with significance thresholds of *p* ≤ 0.05 and *q* ≤ 0.10, and transcriptional using moderated *t* test with significance thresholds of fold-change and |FC | > 2, *p* ≤ 0.05 and *q* ≤ 0.10.

### Statistical analysis

The data generated and analyzed during this study are described in^37^ and correspond to 36 publicly available datasets that are listed in Supplementary Table 8 (all datasets) and in data refs ^7,11,28,34,38–105^. The prognostic clinicopathological variables tested included the patients’ age (≤50 years versus >50), and pathological type (ductal versus lobular versus other), grade (1 versus 2 versus 3), tumor size (pT1 versus pT2 versus pT3), and axillary lymph node status (negative versus positive), and use of adjuvant HT and CT (yes versus no). Distant relapse-free interval was calculated from the date of diagnosis until the date of distant relapse or death from breast cancer. Event-free patients lost to follow-up or dead from unspecified cause or from cause unrelated to breast cancer were censored at time of last contact. The follow-up was calculated from the date of diagnosis until the date of last news for event-free patients using the reverse Kaplan−Meier method. Survivals were calculated using the Kaplan−Meier method and compared with the log-rank test. Uni- and multivariate analyses for DRFI were done using Cox regression analysis (Wald test). The pCR after neoadjuvant chemotherapy was defined as absence of invasive cancer in both breast and axillary lymph nodes (ypT0/Tis-ypN0). Correlations between molecular subtypes and other variables were analyzed using Fisher’s exact test and *t* test (Table 1) and regression analysis with binomial or Gaussian family (Figs. 1 and S4) for discrete or continuous variables respectively. All statistical tests were two-sided at the 5% level of significance. In the case of multiple testing, the *p* values were replaced by the corrected *q* values. Analyses were done in the R software (version 3.5.2) using glm function and the survival package (version 2.44).

### Reporting summary

Further information on research design is available in the Nature Research Reporting Summary linked to this article.

## Supplementary information

Supplementary materials

Reporting Summary

## Data Availability

The data generated and analyzed during this study are described in the following data record: 10.6084/m9.figshare.11558676. The dataset Breast_cancer_classifications.csv supporting Fig. 1, Table 1, and Supplementary Tables 1−3, and containing the clinicopathological and molecular data of tumors analyzed, is publicly available in the figshare repository as part of the data record mentioned above. (GEO, Array Express and EGA datasets).

## Code availability

All codes and scripts used for applying the gene classifiers are available at https://github.com/predictiveoncologyCRCM/NPJbc_2019_ERposBC.
