# Supplementary material for: The therapeutic response of ER+/HER2− breast cancers differs according to the molecular Basal or Luminal subtype
Source: NPJ Breast Cancer. 2020 Mar 6;6:8. doi: 10.1038/s41523-020-0151-5 (PMC7060267; doi:10.1038/s41523-020-0151-5)
Supplement: Supplementary file 1 — Supplementary materials [file 41523_2020_151_MOESM1_ESM.pdf]

Supplementary Figure 1

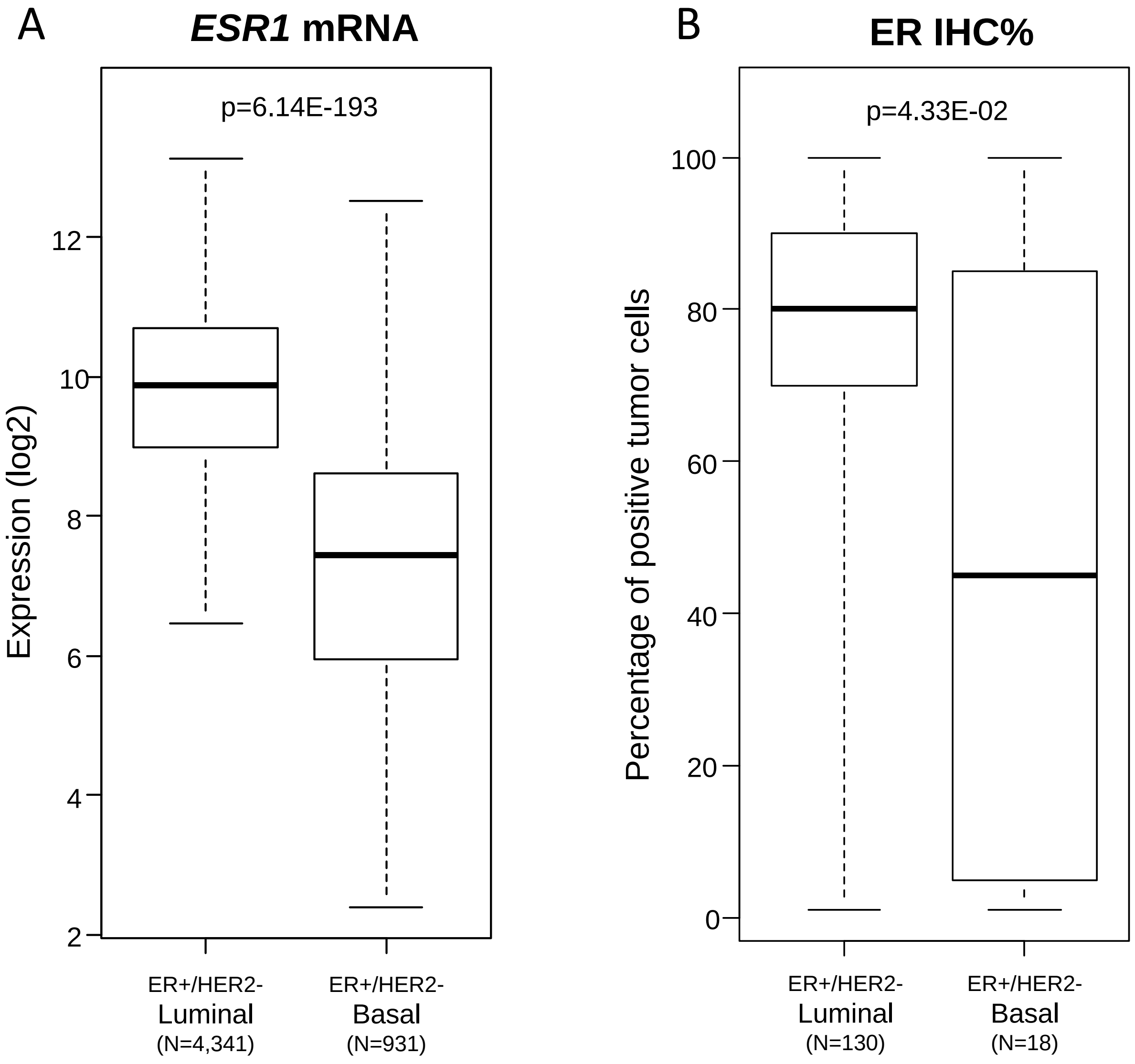

C

ER IHC10%

	N	Negative	Positive
ER+/HER2- Luminal	3,716	132 (4%)	3,584 (96%)
ER+/HER2- Basal	810	98 (12%)	712 (88%)

p=2.33E-19

Supplementary Figure 1: Comparison of ER expression level in the Basal *versus* Luminal ER+/HER2- breast cancers.
a Box plots of *ESR1* mRNA expression level in the Basal subtype (N=931) *versus* Luminal subtype (N=4.341) ER+/HER2- breast cancers. The p-value is for the Student t-test. **b** Similar to a/, but box plots of percentage of positive tumor cells by ER IHC in the Basal subtype (N=18) *versus* Luminal subtype (N=130) ER+/HER2- breast cancers. C/ Cross-table of molecular subtype (Luminal and Basal) *versus* percentage of positive tumor cells ≥10 or <10 by ER IHC. The p-value is for the Fisher's exact test.

Supplementary Figure 2

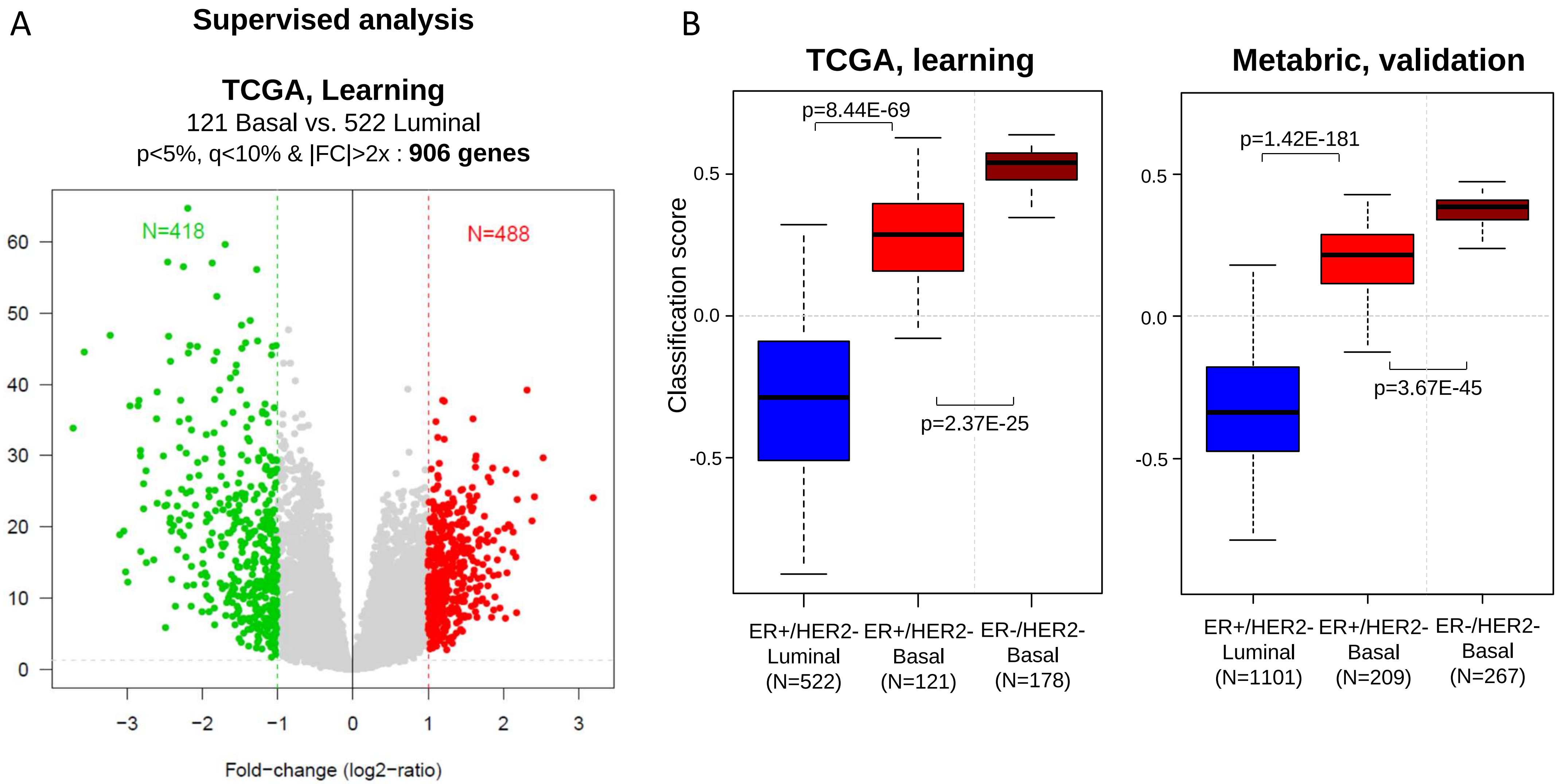

Supplementary Figure 2: Identification and validation of 906 genes differentially expressed between the ER+/HER2- Luminal and Basal breast cancers

a Identification of the signature in the TCGA data set (N=643). Volcano-plot showing the 906 genes differentially expressed between the ER+/HER2- Luminal and Basal breast cancers. **b** Box plot of correlation coefficient of expression profile of each sample with the mean profile of the ER+/HER2- Basal TCGA samples in the learning set (TCGA) and the validation set (Metabric). The horizontal grey line indicates the threshold 0 that separates the two predicted classes of samples, "Basal-like" (above the line) and “Luminal-like “(under the line). The subtype of samples is color-coded as follows: Blue for ER+/HER2- Luminal, red for ER+/HER2- Basal, and brown for ER- basal. The p-value is for the Student t-test.

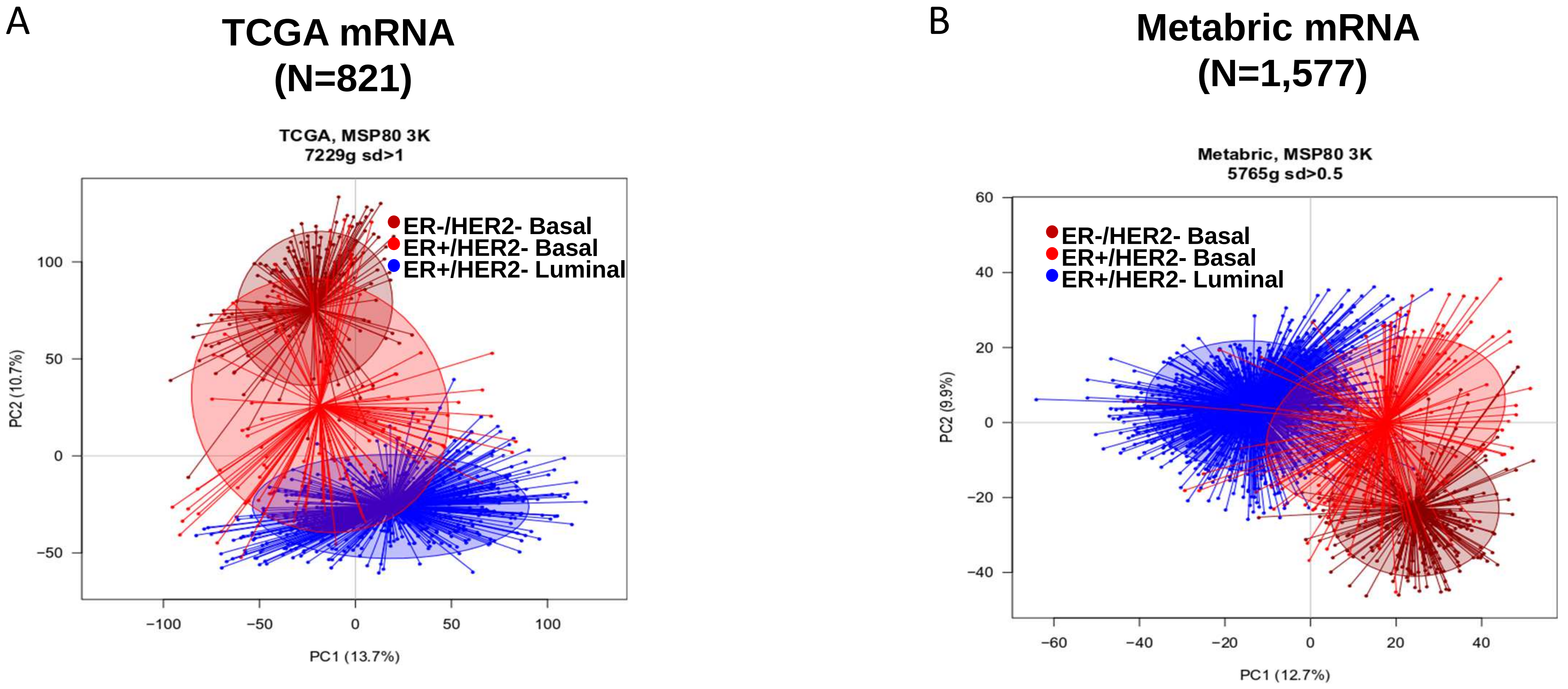

Supplementary Figure 3: Principal Component Analysis (PCA) of breast cancer samples (TCGA and Metabric)
a PCA was applied to the 821 samples (522 ER+/HER2- Luminal, 121 ER+/HER2- Basal, and 178 ER- Basal) and the 7,229 most variable genes (SD≥1.0) extracted from the TCGA data sets. Thesamples are grouped according to their molecular subtype. **b** Similar to a/, but in the 1,577 Metabric samples (1,101 ER+/HER2- Luminal, 209 ER+/HER2- Basal, and 267 ER- Basal).

Supplementary Figure 4

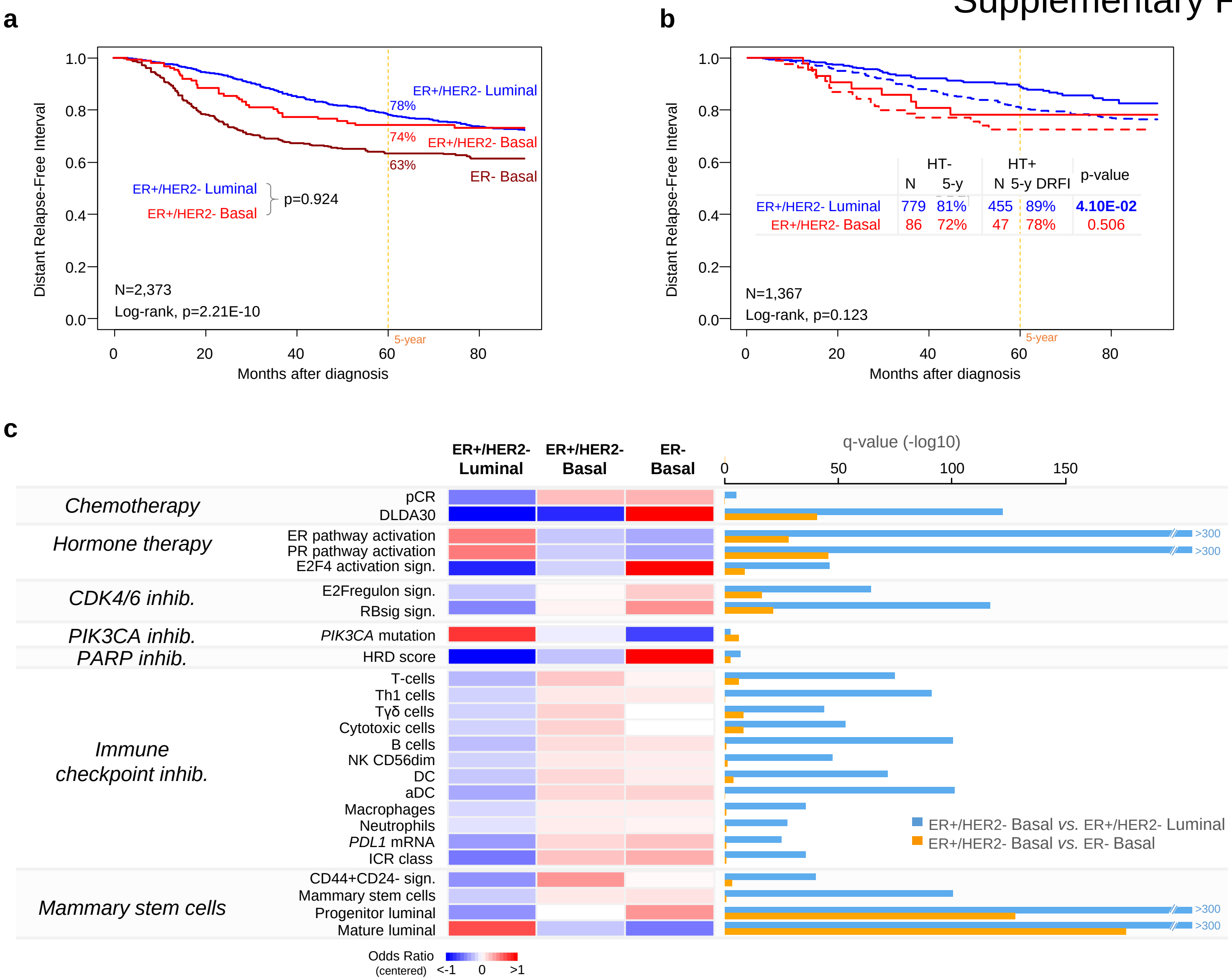

Supplementary Figure 4: Comparison of the ER+/HER2- Basal subtype, ER+/HER2- Luminal subtype; and ER- Basal subtype breast cancers defined using the PAM50 signature

a Kaplan-Meier post-operative DRFI curves in early BCs according to the ER IHC status and the PAM50 type. **b** Similar to a/, but in ER+/HER2- early BCs patients only, untreated (dashed curves) and treated (solid curves) with adjuvant HT. **c** Heatmap of the Odds Ratios (ORs) of regression analysis between the three tumor types (ER+/HER2- Luminal subtype, ER+/HER2- Basal subtype, and ER- Basal subtype used as reference for comparison) for different variables related to the percent of pCR after CT or the probability of therapeutic response of BC to CT, HT, CDK4/6 inhibitors, PIK3CA inhibitor, PARP inhibitors, and immune checkpoint inhibitors. Variables associated to mammary stem cells are also shown. For each variable, the ORs are mean-centered and color-coded according to the color scale shown below. On the right, the bar plots represent the log10-transformed q-values of regression analysis for the comparison of each variable between ER+/HER2- Basal subtype *versus* ER+/HER2- Luminal subtype (blue bar), and between ER+ Basal subtype *versus* ER- Basal subtype (orange bar). The longer is the bar, the lower is the q-value.

Supplementary Table 1: Uni- and multivariate analyses for DRFI in ER+/HER2- breast cancers

Characteristics		Univariate			Multivariate		
		N	HR [95%CI]	p-value	N	HR [95%CI]	p-value
Patients' age	>50 vs. ≤50 years	1403	0.94 [0.72-1.23]	0.635	592	1.20 [0.82-1.75]	0.353
Pathological grade	2 *vs.* 1	946	1.63 [1.10-2.43]	3.54E-10	592	1.52 [0.94-2.46]	0.091
	3 *vs.* 1		3.32 [2.24-4.92]		592	2.96 [1.84-4.76]	7.31E-06
Pathological tumor size (pT)	pT2 *vs.* pT1	1433	1.83 [1.38-2.43]	1.21E-04	592	1.40 [0.98-1.98]	0.064
	pT3 *vs.* pT1		1.71 [1.01-2.90]		592	1.83 [0.83-4.04]	0.136
Pathological axillary lymph node status (pN)	positive *vs.* negative	1738	1.14 [0.90-1.45]	0.272	592	1.69 [1.00-2.86]	5.00E-02
Adjuvant HT	yes *vs.* no	1509	0.67 [0.50-0.91]	9.28E-03	592	0.61 [0.38-0.98]	4.21E-02
Adjuvant CT	yes *vs.* no	1635	0.89 [0.64-1.23]	0.479	592	0.72 [0.42-1.24]	0.233
Molecular subtype	Basal *vs.* Luminal	2008	0.85 [0.65-1.11]	0.240	592	1.24 [0.77-1.98]	0.377

HT, hormone therapy; CT, chemotherapy

Supplementary Table 2: Multivariate analyses for DRFI in ER+/HER2- Luminal and Basal breast cancers

MSP80 definition of molecular subtypes

Luminal	N	Multivariate HR [95%CI]	p-value
Adjuvant HT, yes vs. no	524	0.57 [0.34-0.95]	3.13E-02
Patients' age, >50 vs. ≤50 years	524	1.16 [0.77-1.74]	0.474
Pathological grade, 2 vs. 1	524	1.52 [0.90-2.55]	0.114
3 vs. 1	524	3.28 [1.98-5.44]	4.08E-06
Pathological tumor size (pT), pT2 vs. pT1	524	1.38 [0.94-2.02]	0.096
pT3 vs. pT1	524	1.47 [0.60-3.63]	0.400
Pathological axillary lymph node status (pN), positive vs. negative	524	1.86 [1.04-3.32]	3.63E-02
Adjuvant CT, yes vs. no	524	0.71 [0.39-1.29]	0.255

Basal	N	Multivariate HR [95%CI]	p-value
Adjuvant HT, yes vs. no	68	1.05 [0.23-4.68]	0.952
Patients' age, >50 vs. ≤50 years	68	1.29 [0.40-4.18]	0.667
Pathological grade, 2 vs. 1	68	1.61 [0.39-6.55]	0.508
3 vs. 1	68	2.08 [0.50-8.60]	0.312
Pathological tumor size (pT), pT2 vs. pT1	68	1.28 [0.44-3.77]	0.652
pT3 vs. pT1	68	3.96 [0.45-34.4]	0.213
Pathological axillary lymph node status (pN), positive vs. negative	68	1.28 [0.29-5.67]	0.744
Adjuvant CT, yes vs. no	68	0.83 [0.19-3.64]	0.809

PAM50 definition of molecular subtypes

Luminal	N	Multivariate HR [95%CI]	p-value
Adjuvant HT, yes vs. no	500	0.60 [0.36-0.99]	4.70E-02
Patients' age, >50 vs. ≤50 years	500	1.22 [0.81-1.84]	0.335
Pathological grade, 2 vs. 1	500	1.51 [0.88-2.58]	0.135
3 vs. 1	500	3.31 [1.97-5.56]	6.59E-06
Pathological tumor size (pT), pT2 vs. pT1	500	1.35 [0.92-1.97]	0.125
pT3 vs. pT1	500	1.07 [0.37-3.07]	0.907
Pathological axillary lymph node status (pN), positive vs. negative	500	1.38 [0.77-2.49]	0.278
Adjuvant CT, yes vs. no	500	0.94 [0.51-1.73]	0.841

Basal	N	Multivariate HR [95%CI]	p-value
Adjuvant HT, yes vs. no	40	0.54 [0.07-4.15]	0.555
Patients' age, >50 vs. ≤50 years	40	1.95 [0.27-14.09]	0.508
Pathological grade, 2 vs. 1	40	0.29 [0.03-2.99]	0.300
3 vs. 1	40	0.8 [0.12-5.50]	0.819
Pathological tumor size (pT), pT2 vs. pT1	40	0.95 [0.16-5.61]	0.952
pT3 vs. pT1	40	6.02 [0.44-82.40]	0.179
Pathological axillary lymph node status (pN), positive vs. negative	40	1.92 [0.09-40.35]	0.674
Adjuvant CT, yes vs. no	40	0.91 [0.04-18.87]	0.954

Supplementary Table 3: Frequency of actionable genetic alterations (AGAs) in the ER+/HER2- Luminal and Basal breast cancers (TCGA and Metabric)

AGAs	Clinical evidence level for the target	Luminal			Basal			Basal vs. Luminal		
		N tested	N altered	Fq alteration	N tested	N altered	Fq alteration	OR [95%CI]	p-value	q-value
ERBB2 amplification (>= 6 copies)	1A	1528	1	0.07%	290	1	0.34%	5.28 [0.07-414]	0.294	0.499
PIK3CA mutation	1A	1528	571	37.37%	290	88	30.34%	0.73 [0.55-0.96]	2.35E-02	0.100
ESR1 mutation	2A	1528	1	0.07%	290	0	0.00%	0.00 [0.00-205]	1.000	1.000
PTEN deletion (homozigous)	2A	1528	6	0.39%	290	2	0.69%	1.76 [0.17-9.91]	0.621	0.754
AKT1 mutation	2B	1528	45	2.95%	290	8	2.76%	0.93 [0.38-2.03]	1.000	1.000
ERBB2 mutation	2B	1528	15	0.98%	290	4	1.38%	1.41 [0.34-4.47]	0.528	0.690
FGFR1 amplification	2	1585	122	7.70%	324	30	9.26%	1.22 [0.78-1.88]	0.367	0.567
FGFR2 amplification	2	1585	4	0.25%	324	5	1.54%	6.19 [1.32-31.4]	9.52E-03	0.081
EGFR amplification	2	1585	2	0.13%	324	3	0.93%	7.39 [0.84-88.9]	3.71E-02	0.126
PIK3CA amplification	2	1585	4	0.25%	324	6	1.85%	7.45 [1.75-36.1]	2.61E-03	4.44E-02
AKT1 amplification	2	1585	2	0.13%	324	2	0.62%	4.91 [0.35-68.1]	0.136	0.279
AKT2 amplification	2	1585	2	0.13%	324	1	0.31%	2.45 [0.04-47.2]	0.428	0.606
AKT3 amplification	2	1585	22	1.39%	324	8	2.47%	1.80 [0.69-4.24]	0.148	0.279
KRAS amplification	2	1585	5	0.32%	324	5	1.54%	4.95 [1.13-21.6]	1.64E-02	0.093
BRAF amplification	2	1585	3	0.19%	324	3	0.93%	4.92 [0.66-36.9]	0.065	0.184
INPP4B deletion (homozigous)	2	1585	0	0.00%	324	0	0.00%	0.00 [0.00 - Inf.]	1.000	1.000

Supplementary Table 4: Comparison of frequency of mutations in the ER+/HER2- Luminal and Basal breast cancers among the 1,180 genes mutated in at least 5/579 tested samples (TCGA)

Symbol	Luminal			Basal			Basal vs. Luminal		
	N tested	N altered	Fq alteration	N tested	N altered	Fq alteration	OR [95%CI]	p-value*	q-value
PIK3CA	478	198	41.42%	101	31	30.69%	0.63 [0.38-1.01]	0.057	1.000
TP53	478	56	11.72%	101	39	38.61%	4.72 [2.81-7.92]	1.29E-09	2.17E-05
TTN	478	65	13.60%	101	20	19.80%	1.57 [0.85-2.8]	0.122	1.000
CDH1	478	76	15.90%	101	10	9.90%	0.58 [0.26-1.19]	0.165	1.000
GATA3	478	68	14.23%	101	6	5.94%	0.38 [0.13-0.91]	2.16E-02	1.000
CROCCP2	478	35	7.32%	101	9	8.91%	1.24 [0.51-2.74]	0.540	1.000
MAP3K1	478	51	10.67%	101	5	4.95%	0.44 [0.13-1.13]	0.094	1.000
MUC16	478	30	6.28%	101	9	8.91%	1.46 [0.59-3.29]	0.380	1.000
MUC12	478	25	5.23%	101	9	8.91%	1.77 [0.7-4.08]	0.163	1.000
MLL3	478	39	8.16%	101	6	5.94%	0.71 [0.24-1.76]	0.544	1.000
DMD	478	13	2.72%	101	8	7.92%	3.07 [1.07-8.25]	1.82E-02	1.000
PTEN	478	17	3.56%	101	7	6.93%	2.02 [0.69-5.29]	0.163	1.000
SYNE1	478	17	3.56%	101	6	5.94%	1.71 [0.54-4.7]	0.264	1.000
MACF1	478	16	3.35%	101	6	5.94%	1.82 [0.57-5.06]	0.247	1.000
HMCN1	478	11	2.30%	101	7	6.93%	3.15 [1.01-9.18]	2.41E-02	1.000
CSMD1	478	14	2.93%	101	6	5.94%	2.09 [0.64-5.97]	0.137	1.000
COL6A5	478	12	2.51%	101	6	5.94%	2.45 [0.73-7.26]	0.105	1.000
RYR2	478	12	2.51%	101	6	5.94%	2.45 [0.73-7.26]	0.105	1.000
NF1	478	7	1.46%	101	7	6.93%	4.99 [1.46-17.1]	4.79E-03	1.000
CACNA1A	478	6	1.26%	101	7	6.93%	5.83 [1.64-21.51]	2.80E-03	1.000
HUWE1	478	13	2.72%	101	5	4.95%	1.86 [0.51-5.73]	0.220	1.000
MUC4	478	21	4.39%	101	3	2.97%	0.67 [0.12-2.3]	0.783	1.000
MUC5B	478	21	4.39%	101	3	2.97%	0.67 [0.12-2.3]	0.783	1.000
FLG	478	16	3.35%	101	4	3.96%	1.19 [0.28-3.8]	0.764	1.000
HRNR	478	16	3.35%	101	4	3.96%	1.19 [0.28-3.8]	0.764	1.000
NEB	478	16	3.35%	101	4	3.96%	1.19 [0.28-3.8]	0.764	1.000
NCOR1	478	19	3.97%	101	3	2.97%	0.74 [0.14-2.59]	0.781	1.000
RNA5-8SP6	478	14	2.93%	101	4	3.96%	1.37 [0.32-4.47]	0.535	1.000
NOTCH2	478	9	1.88%	101	5	4.95%	2.71 [0.7-9.23]	0.079	1.000
RUNX1	478	18	3.77%	101	3	2.97%	0.78 [0.14-2.76]	1.000	1.000
RYR3	478	13	2.72%	101	4	3.96%	1.47 [0.34-4.91]	0.515	1.000
SYNE2	478	13	2.72%	101	4	3.96%	1.47 [0.34-4.91]	0.515	1.000
CHD6	478	8	1.67%	101	5	4.95%	3.05 [0.77-10.85]	0.059	1.000
LRBA	478	12	2.51%	101	4	3.96%	1.6 [0.37-5.42]	0.499	1.000
MAP2K4	478	26	5.44%	101	1	0.99%	0.17 [0-1.09]	0.066	1.000
CACNA1E	478	7	1.46%	101	5	4.95%	3.49 [0.86-13.1]	4.17E-02	1.000
PKHD1L1	478	7	1.46%	101	5	4.95%	3.49 [0.86-13.1]	4.17E-02	1.000
GOLGA6L2	478	11	2.30%	101	4	3.96%	1.75 [0.4-6.06]	0.311	1.000
APOBR	478	6	1.26%	101	5	4.95%	4.08 [0.97-16.42]	2.81E-02	1.000
CSMD2	478	6	1.26%	101	5	4.95%	4.08 [0.97-16.42]	2.81E-02	1.000
FAT3	478	6	1.26%	101	5	4.95%	4.08 [0.97-16.42]	2.81E-02	1.000
LRP1B	478	6	1.26%	101	5	4.95%	4.08 [0.97-16.42]	2.81E-02	1.000
CHD4	478	10	2.09%	101	4	3.96%	1.93 [0.43-6.86]	0.282	1.000
HERC2	478	10	2.09%	101	4	3.96%	1.93 [0.43-6.86]	0.282	1.000
ATRX	478	9	1.88%	101	4	3.96%	2.15 [0.47-7.88]	0.257	1.000
DYNC2H1	478	9	1.88%	101	4	3.96%	2.15 [0.47-7.88]	0.257	1.000
LRP1	478	9	1.88%	101	4	3.96%	2.15 [0.47-7.88]	0.257	1.000
ZFHX4	478	9	1.88%	101	4	3.96%	2.15 [0.47-7.88]	0.257	1.000
DNAH7	478	4	0.84%	101	5	4.95%	6.14 [1.3-31.56]	1.03E-02	1.000
PAPPA2	478	4	0.84%	101	5	4.95%	6.14 [1.3-31.56]	1.03E-02	1.000
SPEN	478	18	3.77%	101	2	1.98%	0.52 [0.06-2.22]	0.551	1.000
USH2A	478	13	2.72%	101	3	2.97%	1.09 [0.2-4.09]	0.749	1.000
ABCA8	478	8	1.67%	101	4	3.96%	2.42 [0.52-9.24]	0.238	1.000
GPR98	478	8	1.67%	101	4	3.96%	2.42 [0.52-9.24]	0.238	1.000
ABCA13	478	12	2.51%	101	3	2.97%	1.19 [0.21-4.52]	0.733	1.000
HECTD4	478	12	2.51%	101	3	2.97%	1.19 [0.21-4.52]	0.733	1.000
HS6ST1	478	7	1.46%	101	4	3.96%	2.77 [0.58-11.14]	0.107	1.000
APOB	478	11	2.30%	101	3	2.97%	1.3 [0.23-5.04]	0.720	1.000
FAM157B	478	11	2.30%	101	3	2.97%	1.3 [0.23-5.04]	0.720	1.000
MUC17	478	11	2.30%	101	3	2.97%	1.3 [0.23-5.04]	0.720	1.000
CDH23	478	6	1.26%	101	4	3.96%	3.24 [0.66-13.94]	0.079	1.000
SPTA1	478	6	1.26%	101	4	3.96%	3.24 [0.66-13.94]	0.079	1.000
WDFY4	478	6	1.26%	101	4	3.96%	3.24 [0.66-13.94]	0.079	1.000
ZAN	478	6	1.26%	101	4	3.96%	3.24 [0.66-13.94]	0.079	1.000
HTATSF1	478	1	0.21%	101	5	4.95%	24.64 [2.71-1169.39]	7.67E-04	1.000
FBN3	478	10	2.09%	101	3	2.97%	1.43 [0.25-5.7]	0.483	1.000
HYDIN	478	10	2.09%	101	3	2.97%	1.43 [0.25-5.7]	0.483	1.000
PRUNE2	478	10	2.09%	101	3	2.97%	1.43 [0.25-5.7]	0.483	1.000
CUBN	478	5	1.05%	101	4	3.96%	3.89 [0.76-18.42]	0.054	1.000
GREB1L	478	5	1.05%	101	4	3.96%	3.89 [0.76-18.42]	0.054	1.000
TAF1L	478	5	1.05%	101	4	3.96%	3.89 [0.76-18.42]	0.054	1.000
TBX3	478	19	3.97%	101	1	0.99%	0.24 [0.01-1.56]	0.226	1.000
FMN2	478	9	1.88%	101	3	2.97%	1.59 [0.27-6.54]	0.448	1.000
PARP4	478	9	1.88%	101	3	2.97%	1.59 [0.27-6.54]	0.448	1.000
PDZD2	478	9	1.88%	101	3	2.97%	1.59 [0.27-6.54]	0.448	1.000
DNAH10	478	4	0.84%	101	4	3.96%	4.87 [0.89-26.62]	3.47E-02	1.000
DNAH11	478	4	0.84%	101	4	3.96%	4.87 [0.89-26.62]	3.47E-02	1.000
DOCK5	478	4	0.84%	101	4	3.96%	4.87 [0.89-26.62]	3.47E-02	1.000
LRRK2	478	4	0.84%	101	4	3.96%	4.87 [0.89-26.62]	3.47E-02	1.000
MASP1	478	4	0.84%	101	4	3.96%	4.87 [0.89-26.62]	3.47E-02	1.000
PLEKHG2	478	4	0.84%	101	4	3.96%	4.87 [0.89-26.62]	3.47E-02	1.000
SCN3A	478	4	0.84%	101	4	3.96%	4.87 [0.89-26.62]	3.47E-02	1.000
TMEM132D	478	4	0.84%	101	4	3.96%	4.87 [0.89-26.62]	3.47E-02	1.000
FCGBP	478	13	2.72%	101	2	1.98%	0.72 [0.08-3.27]	1.000	1.000
KIAA1109	478	13	2.72%	101	2	1.98%	0.72 [0.08-3.27]	1.000	1.000
LRP2	478	13	2.72%	101	2	1.98%	0.72 [0.08-3.27]	1.000	1.000
OBSCN	478	13	2.72%	101	2	1.98%	0.72 [0.08-3.27]	1.000	1.000
AKD1	478	8	1.67%	101	3	2.97%	1.8 [0.3-7.65]	0.417	1.000
CEP350	478	8	1.67%	101	3	2.97%	1.8 [0.3-7.65]	0.417	1.000
GRIN2A	478	8	1.67%	101	3	2.97%	1.8 [0.3-7.65]	0.417	1.000
HEATR7B2	478	8	1.67%	101	3	2.97%	1.8 [0.3-7.65]	0.417	1.000
TEX15	478	8	1.67%	101	3	2.97%	1.8 [0.3-7.65]	0.417	1.000
CTTNBP2	478	3	0.63%	101	4	3.96%	6.5 [1.08-45.1]	2.00E-02	1.000
HIST1H3B	478	3	0.63%	101	4	3.96%	6.5 [1.08-45.1]	2.00E-02	1.000
MYH1	478	3	0.63%	101	4	3.96%	6.5 [1.08-45.1]	2.00E-02	1.000
MYO10	478	3	0.63%	101	4	3.96%	6.5 [1.08-45.1]	2.00E-02	1.000
MYOM2	478	3	0.63%	101	4	3.96%	6.5 [1.08-45.1]	2.00E-02	1.000
STAB1	478	3	0.63%	101	4	3.96%	6.5 [1.08-45.1]	2.00E-02	1.000
FRG1B	478	17	3.56%	101	1	0.99%	0.27 [0.01-1.77]	0.338	1.000
AHNAK	478	7	1.46%	101	3	2.97%	2.06 [0.34-9.21]	0.390	1.000
CBLB	478	7	1.46%	101	3	2.97%	2.06 [0.34-9.21]	0.390	1.000
DYNC1H1	478	7	1.46%	101	3	2.97%	2.06 [0.34-9.21]	0.390	1.000
PIK3R1	478	7	1.46%	101	3	2.97%	2.06 [0.34-9.21]	0.390	1.000
PTPRD	478	7	1.46%	101	3	2.97%	2.06 [0.34-9.21]	0.390	1.000
TG	478	7	1.46%	101	3	2.97%	2.06 [0.34-9.21]	0.390	1.000
UTRN	478	7	1.46%	101	3	2.97%	2.06 [0.34-9.21]	0.390	1.000
XIRP2	478	7	1.46%	101	3	2.97%	2.06 [0.34-9.21]	0.390	1.000
JMJD1C	478	2	0.42%	101	4	3.96%	9.76 [1.38-109.3]	9.90E-03	1.000
CBFB	478	16	3.35%	101	1	0.99%	0.29 [0.01-1.9]	0.330	1.000
DST	478	16	3.35%	101	1	0.99%	0.29 [0.01-1.9]	0.330	1.000
SACS	478	11	2.30%	101	2	1.98%	0.86 [0.09-4.02]	1.000	1.000
SDK2	478	11	2.30%	101	2	1.98%	0.86 [0.09-4.02]	1.000	1.000
AHCTF1	478	6	1.26%	101	3	2.97%	2.4 [0.38-11.48]	0.196	1.000
ASTN1	478	6	1.26%	101	3	2.97%	2.4 [0.38-11.48]	0.196	1.000
ATP10B	478	6	1.26%	101	3	2.97%	2.4 [0.38-11.48]	0.196	1.000

Symbol	Luminal			Basal			Basal vs. Luminal		
	N tested	N altered	Fq alteration	N tested	N altered	Fq alteration	OR [95%CI]	p-value*	q-value
COL14A1	478	6	1.26%	101	3	2.97%	2.4 [0.38-11.48]	0.196	1.000
DLG1	478	6	1.26%	101	3	2.97%	2.4 [0.38-11.48]	0.196	1.000
FBN1	478	6	1.26%	101	3	2.97%	2.4 [0.38-11.48]	0.196	1.000
HDAC6	478	6	1.26%	101	3	2.97%	2.4 [0.38-11.48]	0.196	1.000
HIVEP1	478	6	1.26%	101	3	2.97%	2.4 [0.38-11.48]	0.196	1.000
KCNT2	478	6	1.26%	101	3	2.97%	2.4 [0.38-11.48]	0.196	1.000
KDM6A	478	6	1.26%	101	3	2.97%	2.4 [0.38-11.48]	0.196	1.000
PLXNA2	478	6	1.26%	101	3	2.97%	2.4 [0.38-11.48]	0.196	1.000
RELN	478	6	1.26%	101	3	2.97%	2.4 [0.38-11.48]	0.196	1.000
RLF	478	6	1.26%	101	3	2.97%	2.4 [0.38-11.48]	0.196	1.000
TARBP1	478	6	1.26%	101	3	2.97%	2.4 [0.38-11.48]	0.196	1.000
TNRC6B	478	6	1.26%	101	3	2.97%	2.4 [0.38-11.48]	0.196	1.000
FAM5C	478	1	0.21%	101	4	3.96%	19.52 [1.9-965.03]	3.81E-03	1.000
RFX3	478	1	0.21%	101	4	3.96%	19.52 [1.9-965.03]	3.81E-03	1.000
RTL1	478	1	0.21%	101	4	3.96%	19.52 [1.9-965.03]	3.81E-03	1.000
SLC6A9	478	1	0.21%	101	4	3.96%	19.52 [1.9-965.03]	3.81E-03	1.000
ARID1A	478	15	3.14%	101	1	0.99%	0.31 [0.01-2.05]	0.329	1.000
MST1P9	478	10	2.09%	101	2	1.98%	0.95 [0.1-4.54]	1.000	1.000
MUC20	478	10	2.09%	101	2	1.98%	0.95 [0.1-4.54]	1.000	1.000
ODZ1	478	10	2.09%	101	2	1.98%	0.95 [0.1-4.54]	1.000	1.000
PCNXL2	478	10	2.09%	101	2	1.98%	0.95 [0.1-4.54]	1.000	1.000
PREX2	478	10	2.09%	101	2	1.98%	0.95 [0.1-4.54]	1.000	1.000
RYR1	478	10	2.09%	101	2	1.98%	0.95 [0.1-4.54]	1.000	1.000
VPS13C	478	10	2.09%	101	2	1.98%	0.95 [0.1-4.54]	1.000	1.000
BDP1	478	5	1.05%	101	3	2.97%	2.89 [0.44-15.13]	0.148	1.000
DCHS1	478	5	1.05%	101	3	2.97%	2.89 [0.44-15.13]	0.148	1.000
GRIA3	478	5	1.05%	101	3	2.97%	2.89 [0.44-15.13]	0.148	1.000
MTM1	478	5	1.05%	101	3	2.97%	2.89 [0.44-15.13]	0.148	1.000
NACAD	478	5	1.05%	101	3	2.97%	2.89 [0.44-15.13]	0.148	1.000
NIPBL	478	5	1.05%	101	3	2.97%	2.89 [0.44-15.13]	0.148	1.000
PCDHB18	478	5	1.05%	101	3	2.97%	2.89 [0.44-15.13]	0.148	1.000
SSPO	478	5	1.05%	101	3	2.97%	2.89 [0.44-15.13]	0.148	1.000
TANC2	478	5	1.05%	101	3	2.97%	2.89 [0.44-15.13]	0.148	1.000
TARS2	478	5	1.05%	101	3	2.97%	2.89 [0.44-15.13]	0.148	1.000
TMEM247	478	5	1.05%	101	3	2.97%	2.89 [0.44-15.13]	0.148	1.000
TNXB	478	5	1.05%	101	3	2.97%	2.89 [0.44-15.13]	0.148	1.000
TPR	478	5	1.05%	101	3	2.97%	2.89 [0.44-15.13]	0.148	1.000
TRRAP	478	5	1.05%	101	3	2.97%	2.89 [0.44-15.13]	0.148	1.000
WWP2	478	5	1.05%	101	3	2.97%	2.89 [0.44-15.13]	0.148	1.000
MDN1	478	9	1.88%	101	2	1.98%	1.05 [0.11-5.2]	1.000	1.000
MED12	478	9	1.88%	101	2	1.98%	1.05 [0.11-5.2]	1.000	1.000
MYCBP2	478	9	1.88%	101	2	1.98%	1.05 [0.11-5.2]	1.000	1.000
NBPF1	478	9	1.88%	101	2	1.98%	1.05 [0.11-5.2]	1.000	1.000
SCN2A	478	9	1.88%	101	2	1.98%	1.05 [0.11-5.2]	1.000	1.000
ARID4B	478	4	0.84%	101	3	2.97%	3.62 [0.52-21.74]	0.106	1.000
CCDC80	478	4	0.84%	101	3	2.97%	3.62 [0.52-21.74]	0.106	1.000
FAM75D1	478	4	0.84%	101	3	2.97%	3.62 [0.52-21.74]	0.106	1.000
GRIK1	478	4	0.84%	101	3	2.97%	3.62 [0.52-21.74]	0.106	1.000
LYST	478	4	0.84%	101	3	2.97%	3.62 [0.52-21.74]	0.106	1.000
MAGI1	478	4	0.84%	101	3	2.97%	3.62 [0.52-21.74]	0.106	1.000
NWD1	478	4	0.84%	101	3	2.97%	3.62 [0.52-21.74]	0.106	1.000
NXF1	478	4	0.84%	101	3	2.97%	3.62 [0.52-21.74]	0.106	1.000
OTOF	478	4	0.84%	101	3	2.97%	3.62 [0.52-21.74]	0.106	1.000
STK31	478	4	0.84%	101	3	2.97%	3.62 [0.52-21.74]	0.106	1.000
TNRC18	478	4	0.84%	101	3	2.97%	3.62 [0.52-21.74]	0.106	1.000
WDR13	478	4	0.84%	101	3	2.97%	3.62 [0.52-21.74]	0.106	1.000
ZNF541	478	4	0.84%	101	3	2.97%	3.62 [0.52-21.74]	0.106	1.000
PCLO	478	13	2.72%	101	1	0.99%	0.36 [0.01-2.44]	0.482	1.000
DNAH17	478	8	1.67%	101	2	1.98%	1.19 [0.12-6.07]	0.689	1.000
DNAH2	478	8	1.67%	101	2	1.98%	1.19 [0.12-6.07]	0.689	1.000
FREM2	478	8	1.67%	101	2	1.98%	1.19 [0.12-6.07]	0.689	1.000
GRID1	478	8	1.67%	101	2	1.98%	1.19 [0.12-6.07]	0.689	1.000
HCFC1	478	8	1.67%	101	2	1.98%	1.19 [0.12-6.07]	0.689	1.000
MAP2	478	8	1.67%	101	2	1.98%	1.19 [0.12-6.07]	0.689	1.000
MEGF8	478	8	1.67%	101	2	1.98%	1.19 [0.12-6.07]	0.689	1.000
SVEP1	478	8	1.67%	101	2	1.98%	1.19 [0.12-6.07]	0.689	1.000
ALDH1L1	478	3	0.63%	101	3	2.97%	4.83 [0.64-36.58]	0.069	1.000
APBA2	478	3	0.63%	101	3	2.97%	4.83 [0.64-36.58]	0.069	1.000
ASXL3	478	3	0.63%	101	3	2.97%	4.83 [0.64-36.58]	0.069	1.000
CACNA1B	478	3	0.63%	101	3	2.97%	4.83 [0.64-36.58]	0.069	1.000
KDR	478	3	0.63%	101	3	2.97%	4.83 [0.64-36.58]	0.069	1.000
KIF13B	478	3	0.63%	101	3	2.97%	4.83 [0.64-36.58]	0.069	1.000
KIF1B	478	3	0.63%	101	3	2.97%	4.83 [0.64-36.58]	0.069	1.000
KIFC1	478	3	0.63%	101	3	2.97%	4.83 [0.64-36.58]	0.069	1.000
MSH5	478	3	0.63%	101	3	2.97%	4.83 [0.64-36.58]	0.069	1.000
ODF2	478	3	0.63%	101	3	2.97%	4.83 [0.64-36.58]	0.069	1.000
PCDHB2	478	3	0.63%	101	3	2.97%	4.83 [0.64-36.58]	0.069	1.000
PCDHGB6	478	3	0.63%	101	3	2.97%	4.83 [0.64-36.58]	0.069	1.000
PLCB4	478	3	0.63%	101	3	2.97%	4.83 [0.64-36.58]	0.069	1.000
PPFIA2	478	3	0.63%	101	3	2.97%	4.83 [0.64-36.58]	0.069	1.000
PPP1R9A	478	3	0.63%	101	3	2.97%	4.83 [0.64-36.58]	0.069	1.000
PTPRQ	478	3	0.63%	101	3	2.97%	4.83 [0.64-36.58]	0.069	1.000
SCAPER	478	3	0.63%	101	3	2.97%	4.83 [0.64-36.58]	0.069	1.000
SCN10A	478	3	0.63%	101	3	2.97%	4.83 [0.64-36.58]	0.069	1.000
SPATA1	478	3	0.63%	101	3	2.97%	4.83 [0.64-36.58]	0.069	1.000
TLN2	478	3	0.63%	101	3	2.97%	4.83 [0.64-36.58]	0.069	1.000
TXNDC11	478	3	0.63%	101	3	2.97%	4.83 [0.64-36.58]	0.069	1.000
VPS13A	478	3	0.63%	101	3	2.97%	4.83 [0.64-36.58]	0.069	1.000
ZNF208	478	3	0.63%	101	3	2.97%	4.83 [0.64-36.58]	0.069	1.000
ZNF711	478	3	0.63%	101	3	2.97%	4.83 [0.64-36.58]	0.069	1.000
ZNF841	478	3	0.63%	101	3	2.97%	4.83 [0.64-36.58]	0.069	1.000
SF3B1	478	12	2.51%	101	1	0.99%	0.39 [0.01-2.68]	0.709	1.000
UBR4	478	12	2.51%	101	1	0.99%	0.39 [0.01-2.68]	0.709	1.000
AHNAK2	478	7	1.46%	101	2	1.98%	1.36 [0.14-7.28]	0.660	1.000
ANK2	478	7	1.46%	101	2	1.98%	1.36 [0.14-7.28]	0.660	1.000
ATM	478	7	1.46%	101	2	1.98%	1.36 [0.14-7.28]	0.660	1.000
BRWD1	478	7	1.46%	101	2	1.98%	1.36 [0.14-7.28]	0.660	1.000
CCNB3	478	7	1.46%	101	2	1.98%	1.36 [0.14-7.28]	0.660	1.000
COL12A1	478	7	1.46%	101	2	1.98%	1.36 [0.14-7.28]	0.660	1.000
COL6A6	478	7	1.46%	101	2	1.98%	1.36 [0.14-7.28]	0.660	1.000
ERBB2	478	7	1.46%	101	2	1.98%	1.36 [0.14-7.28]	0.660	1.000
FLG2	478	7	1.46%	101	2	1.98%	1.36 [0.14-7.28]	0.660	1.000
KIAA1210	478	7	1.46%	101	2	1.98%	1.36 [0.14-7.28]	0.660	1.000
KIAA1731	478	7	1.46%	101	2	1.98%	1.36 [0.14-7.28]	0.660	1.000
MYB	478	7	1.46%	101	2	1.98%	1.36 [0.14-7.28]	0.660	1.000
SELP	478	7	1.46%	101	2	1.98%	1.36 [0.14-7.28]	0.660	1.000
TAF1	478	7	1.46%	101	2	1.98%	1.36 [0.14-7.28]	0.660	1.000
WNK3	478	7	1.46%	101	2	1.98%	1.36 [0.14-7.28]	0.660	1.000
YLPM1	478	7	1.46%	101	2	1.98%	1.36 [0.14-7.28]	0.660	1.000
ZNF292	478	7	1.46%	101	2	1.98%	1.36 [0.14-7.28]	0.660	1.000
APBA1	478	2	0.42%	101	3	2.97%	7.25 [0.82-88]	3.95E-02	1.000
BAZ1B	478	2	0.42%	101	3	2.97%	7.25 [0.82-88]	3.95E-02	1.000
CACNA2D2	478	2	0.42%	101	3	2.97%	7.25 [0.82-88]	3.95E-02	1.000
CRB1	478	2	0.42%	101	3	2.97%	7.25 [0.82-88]	3.95E-02	1.000
DBC1	478	2	0.42%	101	3	2.97%	7.25 [0.82-88]	3.95E-02	1.000
DLGAP2	478	2	0.42%	101	3	2.97%	7.25 [0.82-88]	3.95E-02	1.000
DQX1	478	2	0.42%	101	3	2.97%	7.25 [0.82-88]	3.95E-02	1.000

Symbol	Luminal			Basal			Basal vs. Luminal		
	N tested	N altered	Fq alteration	N tested	N altered	Fq alteration	OR [95%CI]	p-value*	q-value
FANCD2	478	2	0.42%	101	3	2.97%	7.25 [0.82-88]	3.95E-02	1.000
GRIA1	478	2	0.42%	101	3	2.97%	7.25 [0.82-88]	3.95E-02	1.000
HOOK3	478	2	0.42%	101	3	2.97%	7.25 [0.82-88]	3.95E-02	1.000
IFT140	478	2	0.42%	101	3	2.97%	7.25 [0.82-88]	3.95E-02	1.000
L3MBTL3	478	2	0.42%	101	3	2.97%	7.25 [0.82-88]	3.95E-02	1.000
LIN54	478	2	0.42%	101	3	2.97%	7.25 [0.82-88]	3.95E-02	1.000
NBEA	478	2	0.42%	101	3	2.97%	7.25 [0.82-88]	3.95E-02	1.000
POMT2	478	2	0.42%	101	3	2.97%	7.25 [0.82-88]	3.95E-02	1.000
PPRC1	478	2	0.42%	101	3	2.97%	7.25 [0.82-88]	3.95E-02	1.000
PRDM10	478	2	0.42%	101	3	2.97%	7.25 [0.82-88]	3.95E-02	1.000
PSMD1	478	2	0.42%	101	3	2.97%	7.25 [0.82-88]	3.95E-02	1.000
RBM33	478	2	0.42%	101	3	2.97%	7.25 [0.82-88]	3.95E-02	1.000
SETD1B	478	2	0.42%	101	3	2.97%	7.25 [0.82-88]	3.95E-02	1.000
SPAG17	478	2	0.42%	101	3	2.97%	7.25 [0.82-88]	3.95E-02	1.000
SPAG9	478	2	0.42%	101	3	2.97%	7.25 [0.82-88]	3.95E-02	1.000
SPTBN4	478	2	0.42%	101	3	2.97%	7.25 [0.82-88]	3.95E-02	1.000
TCHH	478	2	0.42%	101	3	2.97%	7.25 [0.82-88]	3.95E-02	1.000
TRIOBP	478	2	0.42%	101	3	2.97%	7.25 [0.82-88]	3.95E-02	1.000
UHRF1BP1L	478	2	0.42%	101	3	2.97%	7.25 [0.82-88]	3.95E-02	1.000
WDR87	478	2	0.42%	101	3	2.97%	7.25 [0.82-88]	3.95E-02	1.000
XPO5	478	2	0.42%	101	3	2.97%	7.25 [0.82-88]	3.95E-02	1.000
ZMYM4	478	2	0.42%	101	3	2.97%	7.25 [0.82-88]	3.95E-02	1.000
ZNF431	478	2	0.42%	101	3	2.97%	7.25 [0.82-88]	3.95E-02	1.000
FOXA1	478	16	3.35%	101	0	0.00%	0 [0-1.21]	0.088	1.000
DNAH12	478	11	2.30%	101	1	0.99%	0.43 [0.01-2.99]	0.702	1.000
USP34	478	11	2.30%	101	1	0.99%	0.43 [0.01-2.99]	0.702	1.000
AKAP9	478	6	1.26%	101	2	1.98%	1.59 [0.15-9.05]	0.634	1.000
BSN	478	6	1.26%	101	2	1.98%	1.59 [0.15-9.05]	0.634	1.000
CADPS	478	6	1.26%	101	2	1.98%	1.59 [0.15-9.05]	0.634	1.000
CELSR2	478	6	1.26%	101	2	1.98%	1.59 [0.15-9.05]	0.634	1.000
CIT	478	6	1.26%	101	2	1.98%	1.59 [0.15-9.05]	0.634	1.000
CNOT1	478	6	1.26%	101	2	1.98%	1.59 [0.15-9.05]	0.634	1.000
DIAPH2	478	6	1.26%	101	2	1.98%	1.59 [0.15-9.05]	0.634	1.000
DOCK10	478	6	1.26%	101	2	1.98%	1.59 [0.15-9.05]	0.634	1.000
MUC2	478	6	1.26%	101	2	1.98%	1.59 [0.15-9.05]	0.634	1.000
NBPF10	478	6	1.26%	101	2	1.98%	1.59 [0.15-9.05]	0.634	1.000
NLGN3	478	6	1.26%	101	2	1.98%	1.59 [0.15-9.05]	0.634	1.000
PLCE1	478	6	1.26%	101	2	1.98%	1.59 [0.15-9.05]	0.634	1.000
RAPGEF6	478	6	1.26%	101	2	1.98%	1.59 [0.15-9.05]	0.634	1.000
RGPD3	478	6	1.26%	101	2	1.98%	1.59 [0.15-9.05]	0.634	1.000
RNF213	478	6	1.26%	101	2	1.98%	1.59 [0.15-9.05]	0.634	1.000
RPGR	478	6	1.26%	101	2	1.98%	1.59 [0.15-9.05]	0.634	1.000
SETX	478	6	1.26%	101	2	1.98%	1.59 [0.15-9.05]	0.634	1.000
TLR4	478	6	1.26%	101	2	1.98%	1.59 [0.15-9.05]	0.634	1.000
TNS1	478	6	1.26%	101	2	1.98%	1.59 [0.15-9.05]	0.634	1.000
CNTRL	478	10	2.09%	101	1	0.99%	0.47 [0.01-3.36]	0.699	1.000
FAT2	478	10	2.09%	101	1	0.99%	0.47 [0.01-3.36]	0.699	1.000
LCT	478	10	2.09%	101	1	0.99%	0.47 [0.01-3.36]	0.699	1.000
MXRA5	478	10	2.09%	101	1	0.99%	0.47 [0.01-3.36]	0.699	1.000
VPS13D	478	10	2.09%	101	1	0.99%	0.47 [0.01-3.36]	0.699	1.000
ANKRD28	478	5	1.05%	101	2	1.98%	1.91 [0.18-11.86]	0.352	1.000
ATP7A	478	5	1.05%	101	2	1.98%	1.91 [0.18-11.86]	0.352	1.000
C9orf174	478	5	1.05%	101	2	1.98%	1.91 [0.18-11.86]	0.352	1.000
CDH8	478	5	1.05%	101	2	1.98%	1.91 [0.18-11.86]	0.352	1.000
CNTLN	478	5	1.05%	101	2	1.98%	1.91 [0.18-11.86]	0.352	1.000
COL4A6	478	5	1.05%	101	2	1.98%	1.91 [0.18-11.86]	0.352	1.000
DCHS2	478	5	1.05%	101	2	1.98%	1.91 [0.18-11.86]	0.352	1.000
DIP2A	478	5	1.05%	101	2	1.98%	1.91 [0.18-11.86]	0.352	1.000
DSCAM	478	5	1.05%	101	2	1.98%	1.91 [0.18-11.86]	0.352	1.000
FAM186A	478	5	1.05%	101	2	1.98%	1.91 [0.18-11.86]	0.352	1.000
FAM86B1	478	5	1.05%	101	2	1.98%	1.91 [0.18-11.86]	0.352	1.000
FAN1	478	5	1.05%	101	2	1.98%	1.91 [0.18-11.86]	0.352	1.000
FLNC	478	5	1.05%	101	2	1.98%	1.91 [0.18-11.86]	0.352	1.000
FRAS1	478	5	1.05%	101	2	1.98%	1.91 [0.18-11.86]	0.352	1.000
HCFC2	478	5	1.05%	101	2	1.98%	1.91 [0.18-11.86]	0.352	1.000
HEATR1	478	5	1.05%	101	2	1.98%	1.91 [0.18-11.86]	0.352	1.000
HEATR5A	478	5	1.05%	101	2	1.98%	1.91 [0.18-11.86]	0.352	1.000
HTT	478	5	1.05%	101	2	1.98%	1.91 [0.18-11.86]	0.352	1.000
IL27RA	478	5	1.05%	101	2	1.98%	1.91 [0.18-11.86]	0.352	1.000
LIFR	478	5	1.05%	101	2	1.98%	1.91 [0.18-11.86]	0.352	1.000
LOXHD1	478	5	1.05%	101	2	1.98%	1.91 [0.18-11.86]	0.352	1.000
MED13L	478	5	1.05%	101	2	1.98%	1.91 [0.18-11.86]	0.352	1.000
MLL	478	5	1.05%	101	2	1.98%	1.91 [0.18-11.86]	0.352	1.000
MYH10	478	5	1.05%	101	2	1.98%	1.91 [0.18-11.86]	0.352	1.000
NBPF12	478	5	1.05%	101	2	1.98%	1.91 [0.18-11.86]	0.352	1.000
NLRP2	478	5	1.05%	101	2	1.98%	1.91 [0.18-11.86]	0.352	1.000
NPAS4	478	5	1.05%	101	2	1.98%	1.91 [0.18-11.86]	0.352	1.000
OTOGL	478	5	1.05%	101	2	1.98%	1.91 [0.18-11.86]	0.352	1.000
PASK	478	5	1.05%	101	2	1.98%	1.91 [0.18-11.86]	0.352	1.000
PCDH11X	478	5	1.05%	101	2	1.98%	1.91 [0.18-11.86]	0.352	1.000
PER3	478	5	1.05%	101	2	1.98%	1.91 [0.18-11.86]	0.352	1.000
PKD1L2	478	5	1.05%	101	2	1.98%	1.91 [0.18-11.86]	0.352	1.000
PLCH1	478	5	1.05%	101	2	1.98%	1.91 [0.18-11.86]	0.352	1.000
POLG	478	5	1.05%	101	2	1.98%	1.91 [0.18-11.86]	0.352	1.000
RALGAPA2	478	5	1.05%	101	2	1.98%	1.91 [0.18-11.86]	0.352	1.000
RB1	478	5	1.05%	101	2	1.98%	1.91 [0.18-11.86]	0.352	1.000
RBM44	478	5	1.05%	101	2	1.98%	1.91 [0.18-11.86]	0.352	1.000
ROBO2	478	5	1.05%	101	2	1.98%	1.91 [0.18-11.86]	0.352	1.000
SCN8A	478	5	1.05%	101	2	1.98%	1.91 [0.18-11.86]	0.352	1.000
SHANK2	478	5	1.05%	101	2	1.98%	1.91 [0.18-11.86]	0.352	1.000
SLIT2	478	5	1.05%	101	2	1.98%	1.91 [0.18-11.86]	0.352	1.000
SPTBN2	478	5	1.05%	101	2	1.98%	1.91 [0.18-11.86]	0.352	1.000
TBX22	478	5	1.05%	101	2	1.98%	1.91 [0.18-11.86]	0.352	1.000
TLN1	478	5	1.05%	101	2	1.98%	1.91 [0.18-11.86]	0.352	1.000
TNRC6A	478	5	1.05%	101	2	1.98%	1.91 [0.18-11.86]	0.352	1.000
TSC22D1	478	5	1.05%	101	2	1.98%	1.91 [0.18-11.86]	0.352	1.000
CTCF	478	14	2.93%	101	0	0.00%	0 [0-1.41]	0.145	1.000
ADAM21P1	478	9	1.88%	101	1	0.99%	0.52 [0.01-3.84]	1.000	1.000
AKAP13	478	9	1.88%	101	1	0.99%	0.52 [0.01-3.84]	1.000	1.000
ANKRD30BL	478	9	1.88%	101	1	0.99%	0.52 [0.01-3.84]	1.000	1.000
CACNA1D	478	9	1.88%	101	1	0.99%	0.52 [0.01-3.84]	1.000	1.000
DNAH9	478	9	1.88%	101	1	0.99%	0.52 [0.01-3.84]	1.000	1.000
DOCK11	478	9	1.88%	101	1	0.99%	0.52 [0.01-3.84]	1.000	1.000
GNAS	478	9	1.88%	101	1	0.99%	0.52 [0.01-3.84]	1.000	1.000
MYO5B	478	9	1.88%	101	1	0.99%	0.52 [0.01-3.84]	1.000	1.000
PDE4DIP	478	9	1.88%	101	1	0.99%	0.52 [0.01-3.84]	1.000	1.000
PLXNA4	478	9	1.88%	101	1	0.99%	0.52 [0.01-3.84]	1.000	1.000
USP9X	478	9	1.88%	101	1	0.99%	0.52 [0.01-3.84]	1.000	1.000
ZFHX3	478	9	1.88%	101	1	0.99%	0.52 [0.01-3.84]	1.000	1.000
ABCC1	478	4	0.84%	101	2	1.98%	2.39 [0.21-16.93]	0.282	1.000
ACVR2A	478	4	0.84%	101	2	1.98%	2.39 [0.21-16.93]	0.282	1.000
ADAMTS6	478	4	0.84%	101	2	1.98%	2.39 [0.21-16.93]	0.282	1.000
AGBL1	478	4	0.84%	101	2	1.98%	2.39 [0.21-16.93]	0.282	1.000
ANKRD17	478	4	0.84%	101	2	1.98%	2.39 [0.21-16.93]	0.282	1.000
ANO4	478	4	0.84%	101	2	1.98%	2.39 [0.21-16.93]	0.282	1.000
ASXL2	478	4	0.84%	101	2	1.98%	2.39 [0.21-16.93]	0.282	1.000
BRCA1	478	4	0.84%	101	2	1.98%	2.39 [0.21-16.93]	0.282	1.000

Symbol	Luminal			Basal			Basal vs. Luminal		
	N tested	N altered	Fq alteration	N tested	N altered	Fq alteration	OR [95%CI]	p-value*	q-value
C2CD3	478	4	0.84%	101	2	1.98%	2.39 [0.21-16.93]	0.282	1.000
CACNA1F	478	4	0.84%	101	2	1.98%	2.39 [0.21-16.93]	0.282	1.000
CAD	478	4	0.84%	101	2	1.98%	2.39 [0.21-16.93]	0.282	1.000
CASC5	478	4	0.84%	101	2	1.98%	2.39 [0.21-16.93]	0.282	1.000
CCDC155	478	4	0.84%	101	2	1.98%	2.39 [0.21-16.93]	0.282	1.000
CDC27	478	4	0.84%	101	2	1.98%	2.39 [0.21-16.93]	0.282	1.000
CTNNA2	478	4	0.84%	101	2	1.98%	2.39 [0.21-16.93]	0.282	1.000
CYB5R1	478	4	0.84%	101	2	1.98%	2.39 [0.21-16.93]	0.282	1.000
DENND4B	478	4	0.84%	101	2	1.98%	2.39 [0.21-16.93]	0.282	1.000
DHX57	478	4	0.84%	101	2	1.98%	2.39 [0.21-16.93]	0.282	1.000
DNMT1	478	4	0.84%	101	2	1.98%	2.39 [0.21-16.93]	0.282	1.000
DROSHA	478	4	0.84%	101	2	1.98%	2.39 [0.21-16.93]	0.282	1.000
EML5	478	4	0.84%	101	2	1.98%	2.39 [0.21-16.93]	0.282	1.000
ERBB3	478	4	0.84%	101	2	1.98%	2.39 [0.21-16.93]	0.282	1.000
ERBB4	478	4	0.84%	101	2	1.98%	2.39 [0.21-16.93]	0.282	1.000
ERCC6	478	4	0.84%	101	2	1.98%	2.39 [0.21-16.93]	0.282	1.000
FER1L6	478	4	0.84%	101	2	1.98%	2.39 [0.21-16.93]	0.282	1.000
FREM3	478	4	0.84%	101	2	1.98%	2.39 [0.21-16.93]	0.282	1.000
HEG1	478	4	0.84%	101	2	1.98%	2.39 [0.21-16.93]	0.282	1.000
KDM5C	478	4	0.84%	101	2	1.98%	2.39 [0.21-16.93]	0.282	1.000
KIAA0913	478	4	0.84%	101	2	1.98%	2.39 [0.21-16.93]	0.282	1.000
LAMA2	478	4	0.84%	101	2	1.98%	2.39 [0.21-16.93]	0.282	1.000
LAMB1	478	4	0.84%	101	2	1.98%	2.39 [0.21-16.93]	0.282	1.000
LARP1	478	4	0.84%	101	2	1.98%	2.39 [0.21-16.93]	0.282	1.000
LPA	478	4	0.84%	101	2	1.98%	2.39 [0.21-16.93]	0.282	1.000
MAGED1	478	4	0.84%	101	2	1.98%	2.39 [0.21-16.93]	0.282	1.000
MORC1	478	4	0.84%	101	2	1.98%	2.39 [0.21-16.93]	0.282	1.000
MRE11A	478	4	0.84%	101	2	1.98%	2.39 [0.21-16.93]	0.282	1.000
MUC6	478	4	0.84%	101	2	1.98%	2.39 [0.21-16.93]	0.282	1.000
MYADML	478	4	0.84%	101	2	1.98%	2.39 [0.21-16.93]	0.282	1.000
MYH11	478	4	0.84%	101	2	1.98%	2.39 [0.21-16.93]	0.282	1.000
MYH14	478	4	0.84%	101	2	1.98%	2.39 [0.21-16.93]	0.282	1.000
MYO7B	478	4	0.84%	101	2	1.98%	2.39 [0.21-16.93]	0.282	1.000
NLRC5	478	4	0.84%	101	2	1.98%	2.39 [0.21-16.93]	0.282	1.000
NUP160	478	4	0.84%	101	2	1.98%	2.39 [0.21-16.93]	0.282	1.000
OGDH	478	4	0.84%	101	2	1.98%	2.39 [0.21-16.93]	0.282	1.000
PCDHB12	478	4	0.84%	101	2	1.98%	2.39 [0.21-16.93]	0.282	1.000
PHF8	478	4	0.84%	101	2	1.98%	2.39 [0.21-16.93]	0.282	1.000
PHKA2	478	4	0.84%	101	2	1.98%	2.39 [0.21-16.93]	0.282	1.000
PIEZO1	478	4	0.84%	101	2	1.98%	2.39 [0.21-16.93]	0.282	1.000
PIK3CB	478	4	0.84%	101	2	1.98%	2.39 [0.21-16.93]	0.282	1.000
PLOD3	478	4	0.84%	101	2	1.98%	2.39 [0.21-16.93]	0.282	1.000
PRRX1	478	4	0.84%	101	2	1.98%	2.39 [0.21-16.93]	0.282	1.000
PTCHD1	478	4	0.84%	101	2	1.98%	2.39 [0.21-16.93]	0.282	1.000
PTPRT	478	4	0.84%	101	2	1.98%	2.39 [0.21-16.93]	0.282	1.000
PTPRU	478	4	0.84%	101	2	1.98%	2.39 [0.21-16.93]	0.282	1.000
RPTOR	478	4	0.84%	101	2	1.98%	2.39 [0.21-16.93]	0.282	1.000
SETD5	478	4	0.84%	101	2	1.98%	2.39 [0.21-16.93]	0.282	1.000
SLC4A11	478	4	0.84%	101	2	1.98%	2.39 [0.21-16.93]	0.282	1.000
SLFN14	478	4	0.84%	101	2	1.98%	2.39 [0.21-16.93]	0.282	1.000
SMC1A	478	4	0.84%	101	2	1.98%	2.39 [0.21-16.93]	0.282	1.000
SPTAN1	478	4	0.84%	101	2	1.98%	2.39 [0.21-16.93]	0.282	1.000
SRRM2	478	4	0.84%	101	2	1.98%	2.39 [0.21-16.93]	0.282	1.000
THSD7B	478	4	0.84%	101	2	1.98%	2.39 [0.21-16.93]	0.282	1.000
TLR8	478	4	0.84%	101	2	1.98%	2.39 [0.21-16.93]	0.282	1.000
TNC	478	4	0.84%	101	2	1.98%	2.39 [0.21-16.93]	0.282	1.000
TNKS1BP1	478	4	0.84%	101	2	1.98%	2.39 [0.21-16.93]	0.282	1.000
TRPM6	478	4	0.84%	101	2	1.98%	2.39 [0.21-16.93]	0.282	1.000
UNC13A	478	4	0.84%	101	2	1.98%	2.39 [0.21-16.93]	0.282	1.000
VAC14	478	4	0.84%	101	2	1.98%	2.39 [0.21-16.93]	0.282	1.000
ZNF646	478	4	0.84%	101	2	1.98%	2.39 [0.21-16.93]	0.282	1.000
ZNF831	478	4	0.84%	101	2	1.98%	2.39 [0.21-16.93]	0.282	1.000
BAZ2B	478	8	1.67%	101	1	0.99%	0.59 [0.01-4.47]	1.000	1.000
CPAMD8	478	8	1.67%	101	1	0.99%	0.59 [0.01-4.47]	1.000	1.000
CYFIP2	478	8	1.67%	101	1	0.99%	0.59 [0.01-4.47]	1.000	1.000
DNAH1	478	8	1.67%	101	1	0.99%	0.59 [0.01-4.47]	1.000	1.000
DNHD1	478	8	1.67%	101	1	0.99%	0.59 [0.01-4.47]	1.000	1.000
DYSF	478	8	1.67%	101	1	0.99%	0.59 [0.01-4.47]	1.000	1.000
EYS	478	8	1.67%	101	1	0.99%	0.59 [0.01-4.47]	1.000	1.000
FAM47C	478	8	1.67%	101	1	0.99%	0.59 [0.01-4.47]	1.000	1.000
FAT1	478	8	1.67%	101	1	0.99%	0.59 [0.01-4.47]	1.000	1.000
FLNA	478	8	1.67%	101	1	0.99%	0.59 [0.01-4.47]	1.000	1.000
HECW1	478	8	1.67%	101	1	0.99%	0.59 [0.01-4.47]	1.000	1.000
MAP1A	478	8	1.67%	101	1	0.99%	0.59 [0.01-4.47]	1.000	1.000
MYH7	478	8	1.67%	101	1	0.99%	0.59 [0.01-4.47]	1.000	1.000
MYH9	478	8	1.67%	101	1	0.99%	0.59 [0.01-4.47]	1.000	1.000
NRXN2	478	8	1.67%	101	1	0.99%	0.59 [0.01-4.47]	1.000	1.000
PCNT	478	8	1.67%	101	1	0.99%	0.59 [0.01-4.47]	1.000	1.000
RAB3GAP2	478	8	1.67%	101	1	0.99%	0.59 [0.01-4.47]	1.000	1.000
TRANK1	478	8	1.67%	101	1	0.99%	0.59 [0.01-4.47]	1.000	1.000
ABCB8	478	3	0.63%	101	2	1.98%	3.19 [0.26-28.23]	0.211	1.000
ACOXL	478	3	0.63%	101	2	1.98%	3.19 [0.26-28.23]	0.211	1.000
ACTG1	478	3	0.63%	101	2	1.98%	3.19 [0.26-28.23]	0.211	1.000
ADAM15	478	3	0.63%	101	2	1.98%	3.19 [0.26-28.23]	0.211	1.000
ANKRD36B	478	3	0.63%	101	2	1.98%	3.19 [0.26-28.23]	0.211	1.000
ANO5	478	3	0.63%	101	2	1.98%	3.19 [0.26-28.23]	0.211	1.000
ARHGAP33	478	3	0.63%	101	2	1.98%	3.19 [0.26-28.23]	0.211	1.000
ARHGEF15	478	3	0.63%	101	2	1.98%	3.19 [0.26-28.23]	0.211	1.000
ARID2	478	3	0.63%	101	2	1.98%	3.19 [0.26-28.23]	0.211	1.000
ASH1L	478	3	0.63%	101	2	1.98%	3.19 [0.26-28.23]	0.211	1.000
ATF6	478	3	0.63%	101	2	1.98%	3.19 [0.26-28.23]	0.211	1.000
ATP1A4	478	3	0.63%	101	2	1.98%	3.19 [0.26-28.23]	0.211	1.000
BCR	478	3	0.63%	101	2	1.98%	3.19 [0.26-28.23]	0.211	1.000
C10orf71	478	3	0.63%	101	2	1.98%	3.19 [0.26-28.23]	0.211	1.000
C14orf37	478	3	0.63%	101	2	1.98%	3.19 [0.26-28.23]	0.211	1.000
C1QTNF5	478	3	0.63%	101	2	1.98%	3.19 [0.26-28.23]	0.211	1.000
CACNA1S	478	3	0.63%	101	2	1.98%	3.19 [0.26-28.23]	0.211	1.000
CCPG1	478	3	0.63%	101	2	1.98%	3.19 [0.26-28.23]	0.211	1.000
CD72	478	3	0.63%	101	2	1.98%	3.19 [0.26-28.23]	0.211	1.000
CDC25C	478	3	0.63%	101	2	1.98%	3.19 [0.26-28.23]	0.211	1.000
CDO1	478	3	0.63%	101	2	1.98%	3.19 [0.26-28.23]	0.211	1.000
CNKSR2	478	3	0.63%	101	2	1.98%	3.19 [0.26-28.23]	0.211	1.000
DCC	478	3	0.63%	101	2	1.98%	3.19 [0.26-28.23]	0.211	1.000
DIAPH1	478	3	0.63%	101	2	1.98%	3.19 [0.26-28.23]	0.211	1.000
DMXL2	478	3	0.63%	101	2	1.98%	3.19 [0.26-28.23]	0.211	1.000
DNAH8	478	3	0.63%	101	2	1.98%	3.19 [0.26-28.23]	0.211	1.000
DOCK9	478	3	0.63%	101	2	1.98%	3.19 [0.26-28.23]	0.211	1.000
DOPEY1	478	3	0.63%	101	2	1.98%	3.19 [0.26-28.23]	0.211	1.000
EIF4ENIF1	478	3	0.63%	101	2	1.98%	3.19 [0.26-28.23]	0.211	1.000
EIF4G1	478	3	0.63%	101	2	1.98%	3.19 [0.26-28.23]	0.211	1.000
FAM123B	478	3	0.63%	101	2	1.98%	3.19 [0.26-28.23]	0.211	1.000
FCRL5	478	3	0.63%	101	2	1.98%	3.19 [0.26-28.23]	0.211	1.000
FHAD1	478	3	0.63%	101	2	1.98%	3.19 [0.26-28.23]	0.211	1.000
G3BP2	478	3	0.63%	101	2	1.98%	3.19 [0.26-28.23]	0.211	1.000
HCN4	478	3	0.63%	101	2	1.98%	3.19 [0.26-28.23]	0.211	1.000
HEPH	478	3	0.63%	101	2	1.98%	3.19 [0.26-28.23]	0.211	1.000
HNRNPUL1	478	3	0.63%	101	2	1.98%	3.19 [0.26-28.23]	0.211	1.000

Symbol	Luminal			Basal			Basal vs. Luminal		
	N tested	N altered	Fq alteration	N tested	N altered	Fq alteration	OR [95%CI]	p-value*	q-value
HSDL2	478	3	0.63%	101	2	1.98%	3.19 [0.26-28.23]	0.211	1.000
IGSF9B	478	3	0.63%	101	2	1.98%	3.19 [0.26-28.23]	0.211	1.000
KCNA1	478	3	0.63%	101	2	1.98%	3.19 [0.26-28.23]	0.211	1.000
KCNU1	478	3	0.63%	101	2	1.98%	3.19 [0.26-28.23]	0.211	1.000
KIAA0947	478	3	0.63%	101	2	1.98%	3.19 [0.26-28.23]	0.211	1.000
KIAA1211	478	3	0.63%	101	2	1.98%	3.19 [0.26-28.23]	0.211	1.000
KIAA1324L	478	3	0.63%	101	2	1.98%	3.19 [0.26-28.23]	0.211	1.000
KIAA1549	478	3	0.63%	101	2	1.98%	3.19 [0.26-28.23]	0.211	1.000
KRAS	478	3	0.63%	101	2	1.98%	3.19 [0.26-28.23]	0.211	1.000
LAMB4	478	3	0.63%	101	2	1.98%	3.19 [0.26-28.23]	0.211	1.000
LTK	478	3	0.63%	101	2	1.98%	3.19 [0.26-28.23]	0.211	1.000
MAGI2	478	3	0.63%	101	2	1.98%	3.19 [0.26-28.23]	0.211	1.000
MAN2B2	478	3	0.63%	101	2	1.98%	3.19 [0.26-28.23]	0.211	1.000
MAP3K4	478	3	0.63%	101	2	1.98%	3.19 [0.26-28.23]	0.211	1.000
MARK1	478	3	0.63%	101	2	1.98%	3.19 [0.26-28.23]	0.211	1.000
MDGA1	478	3	0.63%	101	2	1.98%	3.19 [0.26-28.23]	0.211	1.000
MYH4	478	3	0.63%	101	2	1.98%	3.19 [0.26-28.23]	0.211	1.000
MYO9A	478	3	0.63%	101	2	1.98%	3.19 [0.26-28.23]	0.211	1.000
NEBL	478	3	0.63%	101	2	1.98%	3.19 [0.26-28.23]	0.211	1.000
NLRC4	478	3	0.63%	101	2	1.98%	3.19 [0.26-28.23]	0.211	1.000
OBSL1	478	3	0.63%	101	2	1.98%	3.19 [0.26-28.23]	0.211	1.000
PER2	478	3	0.63%	101	2	1.98%	3.19 [0.26-28.23]	0.211	1.000
PHLPP1	478	3	0.63%	101	2	1.98%	3.19 [0.26-28.23]	0.211	1.000
PLCZ1	478	3	0.63%	101	2	1.98%	3.19 [0.26-28.23]	0.211	1.000
PRB2	478	3	0.63%	101	2	1.98%	3.19 [0.26-28.23]	0.211	1.000
PROSER1	478	3	0.63%	101	2	1.98%	3.19 [0.26-28.23]	0.211	1.000
PSIP1	478	3	0.63%	101	2	1.98%	3.19 [0.26-28.23]	0.211	1.000
PSMC6	478	3	0.63%	101	2	1.98%	3.19 [0.26-28.23]	0.211	1.000
PTPRM	478	3	0.63%	101	2	1.98%	3.19 [0.26-28.23]	0.211	1.000
RAPGEF2	478	3	0.63%	101	2	1.98%	3.19 [0.26-28.23]	0.211	1.000
RBM6	478	3	0.63%	101	2	1.98%	3.19 [0.26-28.23]	0.211	1.000
RPH3A	478	3	0.63%	101	2	1.98%	3.19 [0.26-28.23]	0.211	1.000
SAMD9	478	3	0.63%	101	2	1.98%	3.19 [0.26-28.23]	0.211	1.000
SCAF8	478	3	0.63%	101	2	1.98%	3.19 [0.26-28.23]	0.211	1.000
SEMA4D	478	3	0.63%	101	2	1.98%	3.19 [0.26-28.23]	0.211	1.000
SETBP1	478	3	0.63%	101	2	1.98%	3.19 [0.26-28.23]	0.211	1.000
SLC26A9	478	3	0.63%	101	2	1.98%	3.19 [0.26-28.23]	0.211	1.000
SLCO5A1	478	3	0.63%	101	2	1.98%	3.19 [0.26-28.23]	0.211	1.000
SLX4	478	3	0.63%	101	2	1.98%	3.19 [0.26-28.23]	0.211	1.000
SNTB1	478	3	0.63%	101	2	1.98%	3.19 [0.26-28.23]	0.211	1.000
SORBS2	478	3	0.63%	101	2	1.98%	3.19 [0.26-28.23]	0.211	1.000
SRGAP1	478	3	0.63%	101	2	1.98%	3.19 [0.26-28.23]	0.211	1.000
SSH2	478	3	0.63%	101	2	1.98%	3.19 [0.26-28.23]	0.211	1.000
TBC1D8B	478	3	0.63%	101	2	1.98%	3.19 [0.26-28.23]	0.211	1.000
TCP11	478	3	0.63%	101	2	1.98%	3.19 [0.26-28.23]	0.211	1.000
THSD7A	478	3	0.63%	101	2	1.98%	3.19 [0.26-28.23]	0.211	1.000
TNIK	478	3	0.63%	101	2	1.98%	3.19 [0.26-28.23]	0.211	1.000
TRPC4AP	478	3	0.63%	101	2	1.98%	3.19 [0.26-28.23]	0.211	1.000
TUBGCP2	478	3	0.63%	101	2	1.98%	3.19 [0.26-28.23]	0.211	1.000
VWA8	478	3	0.63%	101	2	1.98%	3.19 [0.26-28.23]	0.211	1.000
WLS	478	3	0.63%	101	2	1.98%	3.19 [0.26-28.23]	0.211	1.000
XPO1	478	3	0.63%	101	2	1.98%	3.19 [0.26-28.23]	0.211	1.000
ZBTB8OS	478	3	0.63%	101	2	1.98%	3.19 [0.26-28.23]	0.211	1.000
ZCCHC11	478	3	0.63%	101	2	1.98%	3.19 [0.26-28.23]	0.211	1.000
ZCWPW1	478	3	0.63%	101	2	1.98%	3.19 [0.26-28.23]	0.211	1.000
ZFP36L2	478	3	0.63%	101	2	1.98%	3.19 [0.26-28.23]	0.211	1.000
ZNF334	478	3	0.63%	101	2	1.98%	3.19 [0.26-28.23]	0.211	1.000
ZNF479	478	3	0.63%	101	2	1.98%	3.19 [0.26-28.23]	0.211	1.000
ZNF595	478	3	0.63%	101	2	1.98%	3.19 [0.26-28.23]	0.211	1.000
ZNFX1	478	3	0.63%	101	2	1.98%	3.19 [0.26-28.23]	0.211	1.000
ANKRD12	478	12	2.51%	101	0	0.00%	0 [0-1.69]	0.139	1.000
FAT4	478	12	2.51%	101	0	0.00%	0 [0-1.69]	0.139	1.000
MED23	478	12	2.51%	101	0	0.00%	0 [0-1.69]	0.139	1.000
ADAMTSL1	478	7	1.46%	101	1	0.99%	0.67 [0.01-5.34]	1.000	1.000
ATP2A1	478	7	1.46%	101	1	0.99%	0.67 [0.01-5.34]	1.000	1.000
CACNA1C	478	7	1.46%	101	1	0.99%	0.67 [0.01-5.34]	1.000	1.000
CASP8	478	7	1.46%	101	1	0.99%	0.67 [0.01-5.34]	1.000	1.000
CDH24	478	7	1.46%	101	1	0.99%	0.67 [0.01-5.34]	1.000	1.000
CHD5	478	7	1.46%	101	1	0.99%	0.67 [0.01-5.34]	1.000	1.000
DNAH5	478	7	1.46%	101	1	0.99%	0.67 [0.01-5.34]	1.000	1.000
DNM1P46	478	7	1.46%	101	1	0.99%	0.67 [0.01-5.34]	1.000	1.000
DSP	478	7	1.46%	101	1	0.99%	0.67 [0.01-5.34]	1.000	1.000
ITPR1	478	7	1.46%	101	1	0.99%	0.67 [0.01-5.34]	1.000	1.000
KIF16B	478	7	1.46%	101	1	0.99%	0.67 [0.01-5.34]	1.000	1.000
KIF21B	478	7	1.46%	101	1	0.99%	0.67 [0.01-5.34]	1.000	1.000
MADD	478	7	1.46%	101	1	0.99%	0.67 [0.01-5.34]	1.000	1.000
NCOA6	478	7	1.46%	101	1	0.99%	0.67 [0.01-5.34]	1.000	1.000
PAPPA	478	7	1.46%	101	1	0.99%	0.67 [0.01-5.34]	1.000	1.000
PCDH19	478	7	1.46%	101	1	0.99%	0.67 [0.01-5.34]	1.000	1.000
RBMXL3	478	7	1.46%	101	1	0.99%	0.67 [0.01-5.34]	1.000	1.000
RGAG1	478	7	1.46%	101	1	0.99%	0.67 [0.01-5.34]	1.000	1.000
RTTN	478	7	1.46%	101	1	0.99%	0.67 [0.01-5.34]	1.000	1.000
SDHAP1	478	7	1.46%	101	1	0.99%	0.67 [0.01-5.34]	1.000	1.000
SIK3	478	7	1.46%	101	1	0.99%	0.67 [0.01-5.34]	1.000	1.000
TBL1XR1	478	7	1.46%	101	1	0.99%	0.67 [0.01-5.34]	1.000	1.000
TGS1	478	7	1.46%	101	1	0.99%	0.67 [0.01-5.34]	1.000	1.000
TLR7	478	7	1.46%	101	1	0.99%	0.67 [0.01-5.34]	1.000	1.000
TP53BP1	478	7	1.46%	101	1	0.99%	0.67 [0.01-5.34]	1.000	1.000
XIST	478	7	1.46%	101	1	0.99%	0.67 [0.01-5.34]	1.000	1.000
ABCA10	478	6	1.26%	101	1	0.99%	0.79 [0.02-6.6]	1.000	1.000
ABCC9	478	6	1.26%	101	1	0.99%	0.79 [0.02-6.6]	1.000	1.000
ACACB	478	6	1.26%	101	1	0.99%	0.79 [0.02-6.6]	1.000	1.000
ADAMTS9	478	6	1.26%	101	1	0.99%	0.79 [0.02-6.6]	1.000	1.000
ADGB	478	6	1.26%	101	1	0.99%	0.79 [0.02-6.6]	1.000	1.000
ANK3	478	6	1.26%	101	1	0.99%	0.79 [0.02-6.6]	1.000	1.000
ANKHD1	478	6	1.26%	101	1	0.99%	0.79 [0.02-6.6]	1.000	1.000
C5orf42	478	6	1.26%	101	1	0.99%	0.79 [0.02-6.6]	1.000	1.000
CASZ1	478	6	1.26%	101	1	0.99%	0.79 [0.02-6.6]	1.000	1.000
CHD8	478	6	1.26%	101	1	0.99%	0.79 [0.02-6.6]	1.000	1.000
CMYA5	478	6	1.26%	101	1	0.99%	0.79 [0.02-6.6]	1.000	1.000
COL4A5	478	6	1.26%	101	1	0.99%	0.79 [0.02-6.6]	1.000	1.000
EP300	478	6	1.26%	101	1	0.99%	0.79 [0.02-6.6]	1.000	1.000
EP400	478	6	1.26%	101	1	0.99%	0.79 [0.02-6.6]	1.000	1.000
ERBB2IP	478	6	1.26%	101	1	0.99%	0.79 [0.02-6.6]	1.000	1.000
ESPNP	478	6	1.26%	101	1	0.99%	0.79 [0.02-6.6]	1.000	1.000
F8	478	6	1.26%	101	1	0.99%	0.79 [0.02-6.6]	1.000	1.000
FAM182B	478	6	1.26%	101	1	0.99%	0.79 [0.02-6.6]	1.000	1.000
FAM47B	478	6	1.26%	101	1	0.99%	0.79 [0.02-6.6]	1.000	1.000
FRMD4A	478	6	1.26%	101	1	0.99%	0.79 [0.02-6.6]	1.000	1.000
FRMPD4	478	6	1.26%	101	1	0.99%	0.79 [0.02-6.6]	1.000	1.000
GPR179	478	6	1.26%	101	1	0.99%	0.79 [0.02-6.6]	1.000	1.000
GRIN2B	478	6	1.26%	101	1	0.99%	0.79 [0.02-6.6]	1.000	1.000
GUCY2F	478	6	1.26%	101	1	0.99%	0.79 [0.02-6.6]	1.000	1.000
HEATR8	478	6	1.26%	101	1	0.99%	0.79 [0.02-6.6]	1.000	1.000
LRRC16B	478	6	1.26%	101	1	0.99%	0.79 [0.02-6.6]	1.000	1.000
LRRIQ1	478	6	1.26%	101	1	0.99%	0.79 [0.02-6.6]	1.000	1.000
LTBP2	478	6	1.26%	101	1	0.99%	0.79 [0.02-6.6]	1.000	1.000

Symbol	Luminal			Basal			Basal vs. Luminal		
	N tested	N altered	Fq alteration	N tested	N altered	Fq alteration	OR [95%CI]	p-value*	q-value
MICAL3	478	6	1.26%	101	1	0.99%	0.79 [0.02-6.6]	1.000	1.000
MLLT4	478	6	1.26%	101	1	0.99%	0.79 [0.02-6.6]	1.000	1.000
MTOR	478	6	1.26%	101	1	0.99%	0.79 [0.02-6.6]	1.000	1.000
MUC19	478	6	1.26%	101	1	0.99%	0.79 [0.02-6.6]	1.000	1.000
NOL6	478	6	1.26%	101	1	0.99%	0.79 [0.02-6.6]	1.000	1.000
NPHP4	478	6	1.26%	101	1	0.99%	0.79 [0.02-6.6]	1.000	1.000
PCDH10	478	6	1.26%	101	1	0.99%	0.79 [0.02-6.6]	1.000	1.000
PCDHB7	478	6	1.26%	101	1	0.99%	0.79 [0.02-6.6]	1.000	1.000
PIWIL1	478	6	1.26%	101	1	0.99%	0.79 [0.02-6.6]	1.000	1.000
PKD1	478	6	1.26%	101	1	0.99%	0.79 [0.02-6.6]	1.000	1.000
PRKCQ	478	6	1.26%	101	1	0.99%	0.79 [0.02-6.6]	1.000	1.000
PRPF8	478	6	1.26%	101	1	0.99%	0.79 [0.02-6.6]	1.000	1.000
QSER1	478	6	1.26%	101	1	0.99%	0.79 [0.02-6.6]	1.000	1.000
RBMX	478	6	1.26%	101	1	0.99%	0.79 [0.02-6.6]	1.000	1.000
REV3L	478	6	1.26%	101	1	0.99%	0.79 [0.02-6.6]	1.000	1.000
RIMS2	478	6	1.26%	101	1	0.99%	0.79 [0.02-6.6]	1.000	1.000
SCN1A	478	6	1.26%	101	1	0.99%	0.79 [0.02-6.6]	1.000	1.000
sept-10	478	6	1.26%	101	1	0.99%	0.79 [0.02-6.6]	1.000	1.000
SRCAP	478	6	1.26%	101	1	0.99%	0.79 [0.02-6.6]	1.000	1.000
TEP1	478	6	1.26%	101	1	0.99%	0.79 [0.02-6.6]	1.000	1.000
TRAPPC8	478	6	1.26%	101	1	0.99%	0.79 [0.02-6.6]	1.000	1.000
UNC13C	478	6	1.26%	101	1	0.99%	0.79 [0.02-6.6]	1.000	1.000
UNC79	478	6	1.26%	101	1	0.99%	0.79 [0.02-6.6]	1.000	1.000
WDR72	478	6	1.26%	101	1	0.99%	0.79 [0.02-6.6]	1.000	1.000
ZNF592	478	6	1.26%	101	1	0.99%	0.79 [0.02-6.6]	1.000	1.000
MGA	478	10	2.09%	101	0	0.00%	0 [0-2.11]	0.223	1.000
ABCA1	478	5	1.05%	101	1	0.99%	0.95 [0.02-8.59]	1.000	1.000
ABCA11P	478	5	1.05%	101	1	0.99%	0.95 [0.02-8.59]	1.000	1.000
ACTL6B	478	5	1.05%	101	1	0.99%	0.95 [0.02-8.59]	1.000	1.000
ADAMTS12	478	5	1.05%	101	1	0.99%	0.95 [0.02-8.59]	1.000	1.000
AGAP7	478	5	1.05%	101	1	0.99%	0.95 [0.02-8.59]	1.000	1.000
AKAP11	478	5	1.05%	101	1	0.99%	0.95 [0.02-8.59]	1.000	1.000
AKAP6	478	5	1.05%	101	1	0.99%	0.95 [0.02-8.59]	1.000	1.000
AKR1CL1	478	5	1.05%	101	1	0.99%	0.95 [0.02-8.59]	1.000	1.000
ALMS1	478	5	1.05%	101	1	0.99%	0.95 [0.02-8.59]	1.000	1.000
ASPM	478	5	1.05%	101	1	0.99%	0.95 [0.02-8.59]	1.000	1.000
ATN1	478	5	1.05%	101	1	0.99%	0.95 [0.02-8.59]	1.000	1.000
ATXN2	478	5	1.05%	101	1	0.99%	0.95 [0.02-8.59]	1.000	1.000
BCORL1	478	5	1.05%	101	1	0.99%	0.95 [0.02-8.59]	1.000	1.000
BIRC6	478	5	1.05%	101	1	0.99%	0.95 [0.02-8.59]	1.000	1.000
BMPR2	478	5	1.05%	101	1	0.99%	0.95 [0.02-8.59]	1.000	1.000
BRD8	478	5	1.05%	101	1	0.99%	0.95 [0.02-8.59]	1.000	1.000
BRWD3	478	5	1.05%	101	1	0.99%	0.95 [0.02-8.59]	1.000	1.000
C1orf173	478	5	1.05%	101	1	0.99%	0.95 [0.02-8.59]	1.000	1.000
CACNA1G	478	5	1.05%	101	1	0.99%	0.95 [0.02-8.59]	1.000	1.000
CDH20	478	5	1.05%	101	1	0.99%	0.95 [0.02-8.59]	1.000	1.000
CDK5RAP2	478	5	1.05%	101	1	0.99%	0.95 [0.02-8.59]	1.000	1.000
CELP	478	5	1.05%	101	1	0.99%	0.95 [0.02-8.59]	1.000	1.000
CELSR3	478	5	1.05%	101	1	0.99%	0.95 [0.02-8.59]	1.000	1.000
CEP170	478	5	1.05%	101	1	0.99%	0.95 [0.02-8.59]	1.000	1.000
CHD3	478	5	1.05%	101	1	0.99%	0.95 [0.02-8.59]	1.000	1.000
CILP	478	5	1.05%	101	1	0.99%	0.95 [0.02-8.59]	1.000	1.000
CLEC16A	478	5	1.05%	101	1	0.99%	0.95 [0.02-8.59]	1.000	1.000
CRNKL1	478	5	1.05%	101	1	0.99%	0.95 [0.02-8.59]	1.000	1.000
CROCC	478	5	1.05%	101	1	0.99%	0.95 [0.02-8.59]	1.000	1.000
DENND2C	478	5	1.05%	101	1	0.99%	0.95 [0.02-8.59]	1.000	1.000
DLGAP4	478	5	1.05%	101	1	0.99%	0.95 [0.02-8.59]	1.000	1.000
DMXL1	478	5	1.05%	101	1	0.99%	0.95 [0.02-8.59]	1.000	1.000
DOCK3	478	5	1.05%	101	1	0.99%	0.95 [0.02-8.59]	1.000	1.000
DOCK8	478	5	1.05%	101	1	0.99%	0.95 [0.02-8.59]	1.000	1.000
DPP10	478	5	1.05%	101	1	0.99%	0.95 [0.02-8.59]	1.000	1.000
DPP4	478	5	1.05%	101	1	0.99%	0.95 [0.02-8.59]	1.000	1.000
DSPP	478	5	1.05%	101	1	0.99%	0.95 [0.02-8.59]	1.000	1.000
F5	478	5	1.05%	101	1	0.99%	0.95 [0.02-8.59]	1.000	1.000
FAM135B	478	5	1.05%	101	1	0.99%	0.95 [0.02-8.59]	1.000	1.000
FAM171A1	478	5	1.05%	101	1	0.99%	0.95 [0.02-8.59]	1.000	1.000
FAM208B	478	5	1.05%	101	1	0.99%	0.95 [0.02-8.59]	1.000	1.000
FBXW7	478	5	1.05%	101	1	0.99%	0.95 [0.02-8.59]	1.000	1.000
FREM1	478	5	1.05%	101	1	0.99%	0.95 [0.02-8.59]	1.000	1.000
GCN1L1	478	5	1.05%	101	1	0.99%	0.95 [0.02-8.59]	1.000	1.000
GOLGA6L6	478	5	1.05%	101	1	0.99%	0.95 [0.02-8.59]	1.000	1.000
GOLGB1	478	5	1.05%	101	1	0.99%	0.95 [0.02-8.59]	1.000	1.000
IGSF1	478	5	1.05%	101	1	0.99%	0.95 [0.02-8.59]	1.000	1.000
IMPG1	478	5	1.05%	101	1	0.99%	0.95 [0.02-8.59]	1.000	1.000
IQSEC2	478	5	1.05%	101	1	0.99%	0.95 [0.02-8.59]	1.000	1.000
ITIH6	478	5	1.05%	101	1	0.99%	0.95 [0.02-8.59]	1.000	1.000
KAT6B	478	5	1.05%	101	1	0.99%	0.95 [0.02-8.59]	1.000	1.000
KIF14	478	5	1.05%	101	1	0.99%	0.95 [0.02-8.59]	1.000	1.000
LINC00200	478	5	1.05%	101	1	0.99%	0.95 [0.02-8.59]	1.000	1.000
MAMLD1	478	5	1.05%	101	1	0.99%	0.95 [0.02-8.59]	1.000	1.000
MGAM	478	5	1.05%	101	1	0.99%	0.95 [0.02-8.59]	1.000	1.000
MLL2	478	5	1.05%	101	1	0.99%	0.95 [0.02-8.59]	1.000	1.000
NUP214	478	5	1.05%	101	1	0.99%	0.95 [0.02-8.59]	1.000	1.000
NYNRIN	478	5	1.05%	101	1	0.99%	0.95 [0.02-8.59]	1.000	1.000
PCDH15	478	5	1.05%	101	1	0.99%	0.95 [0.02-8.59]	1.000	1.000
PDGFRB	478	5	1.05%	101	1	0.99%	0.95 [0.02-8.59]	1.000	1.000
PITPNM1	478	5	1.05%	101	1	0.99%	0.95 [0.02-8.59]	1.000	1.000
PKD1L1	478	5	1.05%	101	1	0.99%	0.95 [0.02-8.59]	1.000	1.000
PKD1L3	478	5	1.05%	101	1	0.99%	0.95 [0.02-8.59]	1.000	1.000
PLA2G4D	478	5	1.05%	101	1	0.99%	0.95 [0.02-8.59]	1.000	1.000
PLXNA1	478	5	1.05%	101	1	0.99%	0.95 [0.02-8.59]	1.000	1.000
POLE	478	5	1.05%	101	1	0.99%	0.95 [0.02-8.59]	1.000	1.000
PTPRB	478	5	1.05%	101	1	0.99%	0.95 [0.02-8.59]	1.000	1.000
RBM19	478	5	1.05%	101	1	0.99%	0.95 [0.02-8.59]	1.000	1.000
RGPD4	478	5	1.05%	101	1	0.99%	0.95 [0.02-8.59]	1.000	1.000
ROBO1	478	5	1.05%	101	1	0.99%	0.95 [0.02-8.59]	1.000	1.000
ROS1	478	5	1.05%	101	1	0.99%	0.95 [0.02-8.59]	1.000	1.000
SCN11A	478	5	1.05%	101	1	0.99%	0.95 [0.02-8.59]	1.000	1.000
SEC24B	478	5	1.05%	101	1	0.99%	0.95 [0.02-8.59]	1.000	1.000
SEMA5A	478	5	1.05%	101	1	0.99%	0.95 [0.02-8.59]	1.000	1.000
SMARCC2	478	5	1.05%	101	1	0.99%	0.95 [0.02-8.59]	1.000	1.000
SMG1	478	5	1.05%	101	1	0.99%	0.95 [0.02-8.59]	1.000	1.000
SOGA1	478	5	1.05%	101	1	0.99%	0.95 [0.02-8.59]	1.000	1.000
STAG1	478	5	1.05%	101	1	0.99%	0.95 [0.02-8.59]	1.000	1.000
STAG2	478	5	1.05%	101	1	0.99%	0.95 [0.02-8.59]	1.000	1.000
SYCP1	478	5	1.05%	101	1	0.99%	0.95 [0.02-8.59]	1.000	1.000
TAB3	478	5	1.05%	101	1	0.99%	0.95 [0.02-8.59]	1.000	1.000
TACC2	478	5	1.05%	101	1	0.99%	0.95 [0.02-8.59]	1.000	1.000
TIAM2	478	5	1.05%	101	1	0.99%	0.95 [0.02-8.59]	1.000	1.000
TPTE	478	5	1.05%	101	1	0.99%	0.95 [0.02-8.59]	1.000	1.000
TRPC4	478	5	1.05%	101	1	0.99%	0.95 [0.02-8.59]	1.000	1.000
UBE4B	478	5	1.05%	101	1	0.99%	0.95 [0.02-8.59]	1.000	1.000
UMODL1	478	5	1.05%	101	1	0.99%	0.95 [0.02-8.59]	1.000	1.000
UNC5D	478	5	1.05%	101	1	0.99%	0.95 [0.02-8.59]	1.000	1.000
UTP20	478	5	1.05%	101	1	0.99%	0.95 [0.02-8.59]	1.000	1.000
VWA3A	478	5	1.05%	101	1	0.99%	0.95 [0.02-8.59]	1.000	1.000
VWA3B	478	5	1.05%	101	1	0.99%	0.95 [0.02-8.59]	1.000	1.000

Symbol	Luminal			Basal			Basal vs. Luminal		
	N tested	N altered	Fq alteration	N tested	N altered	Fq alteration	OR [95%CI]	p-value*	q-value
WDR44	478	5	1.05%	101	1	0.99%	0.95 [0.02-8.59]	1.000	1.000
XIRP1	478	5	1.05%	101	1	0.99%	0.95 [0.02-8.59]	1.000	1.000
ZCCHC6	478	5	1.05%	101	1	0.99%	0.95 [0.02-8.59]	1.000	1.000
ZFPM2	478	5	1.05%	101	1	0.99%	0.95 [0.02-8.59]	1.000	1.000
ZMYM2	478	5	1.05%	101	1	0.99%	0.95 [0.02-8.59]	1.000	1.000
ZMYM3	478	5	1.05%	101	1	0.99%	0.95 [0.02-8.59]	1.000	1.000
ZNF362	478	5	1.05%	101	1	0.99%	0.95 [0.02-8.59]	1.000	1.000
ZNF425	478	5	1.05%	101	1	0.99%	0.95 [0.02-8.59]	1.000	1.000
ZNF462	478	5	1.05%	101	1	0.99%	0.95 [0.02-8.59]	1.000	1.000
ZNF540	478	5	1.05%	101	1	0.99%	0.95 [0.02-8.59]	1.000	1.000
ZP4	478	5	1.05%	101	1	0.99%	0.95 [0.02-8.59]	1.000	1.000
ZZEF1	478	5	1.05%	101	1	0.99%	0.95 [0.02-8.59]	1.000	1.000
CD163L1	478	9	1.88%	101	0	0.00%	0 [0-2.4]	0.371	1.000
CDK12	478	9	1.88%	101	0	0.00%	0 [0-2.4]	0.371	1.000
GPS2	478	9	1.88%	101	0	0.00%	0 [0-2.4]	0.371	1.000
MALAT1	478	9	1.88%	101	0	0.00%	0 [0-2.4]	0.371	1.000
ODZ2	478	9	1.88%	101	0	0.00%	0 [0-2.4]	0.371	1.000
PRKDC	478	9	1.88%	101	0	0.00%	0 [0-2.4]	0.371	1.000
ABCA12	478	4	0.84%	101	1	0.99%	1.18 [0.02-12.14]	1.000	1.000
ABCB7	478	4	0.84%	101	1	0.99%	1.18 [0.02-12.14]	1.000	1.000
ABCC3	478	4	0.84%	101	1	0.99%	1.18 [0.02-12.14]	1.000	1.000
ADAMTS13	478	4	0.84%	101	1	0.99%	1.18 [0.02-12.14]	1.000	1.000
ADAMTS19	478	4	0.84%	101	1	0.99%	1.18 [0.02-12.14]	1.000	1.000
ADCY5	478	4	0.84%	101	1	0.99%	1.18 [0.02-12.14]	1.000	1.000
ADCY9	478	4	0.84%	101	1	0.99%	1.18 [0.02-12.14]	1.000	1.000
ADNP	478	4	0.84%	101	1	0.99%	1.18 [0.02-12.14]	1.000	1.000
ALMS1P	478	4	0.84%	101	1	0.99%	1.18 [0.02-12.14]	1.000	1.000
ANKRD30B	478	4	0.84%	101	1	0.99%	1.18 [0.02-12.14]	1.000	1.000
ANPEP	478	4	0.84%	101	1	0.99%	1.18 [0.02-12.14]	1.000	1.000
AP4M1	478	4	0.84%	101	1	0.99%	1.18 [0.02-12.14]	1.000	1.000
ARHGAP21	478	4	0.84%	101	1	0.99%	1.18 [0.02-12.14]	1.000	1.000
ARHGAP25	478	4	0.84%	101	1	0.99%	1.18 [0.02-12.14]	1.000	1.000
ARHGAP29	478	4	0.84%	101	1	0.99%	1.18 [0.02-12.14]	1.000	1.000
ARHGAP35	478	4	0.84%	101	1	0.99%	1.18 [0.02-12.14]	1.000	1.000
ATAD2	478	4	0.84%	101	1	0.99%	1.18 [0.02-12.14]	1.000	1.000
ATP11C	478	4	0.84%	101	1	0.99%	1.18 [0.02-12.14]	1.000	1.000
ATP8B3	478	4	0.84%	101	1	0.99%	1.18 [0.02-12.14]	1.000	1.000
ATP9A	478	4	0.84%	101	1	0.99%	1.18 [0.02-12.14]	1.000	1.000
BACH2	478	4	0.84%	101	1	0.99%	1.18 [0.02-12.14]	1.000	1.000
BNC1	478	4	0.84%	101	1	0.99%	1.18 [0.02-12.14]	1.000	1.000
BRCA2	478	4	0.84%	101	1	0.99%	1.18 [0.02-12.14]	1.000	1.000
C17orf97	478	4	0.84%	101	1	0.99%	1.18 [0.02-12.14]	1.000	1.000
C1orf101	478	4	0.84%	101	1	0.99%	1.18 [0.02-12.14]	1.000	1.000
C1orf222	478	4	0.84%	101	1	0.99%	1.18 [0.02-12.14]	1.000	1.000
C6orf118	478	4	0.84%	101	1	0.99%	1.18 [0.02-12.14]	1.000	1.000
CALHM2	478	4	0.84%	101	1	0.99%	1.18 [0.02-12.14]	1.000	1.000
CAMSAP2	478	4	0.84%	101	1	0.99%	1.18 [0.02-12.14]	1.000	1.000
CAPN2	478	4	0.84%	101	1	0.99%	1.18 [0.02-12.14]	1.000	1.000
CASP8AP2	478	4	0.84%	101	1	0.99%	1.18 [0.02-12.14]	1.000	1.000
CDC73	478	4	0.84%	101	1	0.99%	1.18 [0.02-12.14]	1.000	1.000
CEP128	478	4	0.84%	101	1	0.99%	1.18 [0.02-12.14]	1.000	1.000
CEP192	478	4	0.84%	101	1	0.99%	1.18 [0.02-12.14]	1.000	1.000
CEP250	478	4	0.84%	101	1	0.99%	1.18 [0.02-12.14]	1.000	1.000
CFH	478	4	0.84%	101	1	0.99%	1.18 [0.02-12.14]	1.000	1.000
CFTR	478	4	0.84%	101	1	0.99%	1.18 [0.02-12.14]	1.000	1.000
CHI3L1	478	4	0.84%	101	1	0.99%	1.18 [0.02-12.14]	1.000	1.000
CLEC18B	478	4	0.84%	101	1	0.99%	1.18 [0.02-12.14]	1.000	1.000
CNTN1	478	4	0.84%	101	1	0.99%	1.18 [0.02-12.14]	1.000	1.000
CNTN6	478	4	0.84%	101	1	0.99%	1.18 [0.02-12.14]	1.000	1.000
COL16A1	478	4	0.84%	101	1	0.99%	1.18 [0.02-12.14]	1.000	1.000
COL17A1	478	4	0.84%	101	1	0.99%	1.18 [0.02-12.14]	1.000	1.000
COL1A2	478	4	0.84%	101	1	0.99%	1.18 [0.02-12.14]	1.000	1.000
COL7A1	478	4	0.84%	101	1	0.99%	1.18 [0.02-12.14]	1.000	1.000
CPT1C	478	4	0.84%	101	1	0.99%	1.18 [0.02-12.14]	1.000	1.000
CRYBG3	478	4	0.84%	101	1	0.99%	1.18 [0.02-12.14]	1.000	1.000
CYP11B1	478	4	0.84%	101	1	0.99%	1.18 [0.02-12.14]	1.000	1.000
DENND2D	478	4	0.84%	101	1	0.99%	1.18 [0.02-12.14]	1.000	1.000
DENND3	478	4	0.84%	101	1	0.99%	1.18 [0.02-12.14]	1.000	1.000
DNAH14	478	4	0.84%	101	1	0.99%	1.18 [0.02-12.14]	1.000	1.000
DTX2P1-UPK3BP1-	478	4	0.84%	101	1	0.99%	1.18 [0.02-12.14]	1.000	1.000
EBF1	478	4	0.84%	101	1	0.99%	1.18 [0.02-12.14]	1.000	1.000
EFHA2	478	4	0.84%	101	1	0.99%	1.18 [0.02-12.14]	1.000	1.000
EHBP1	478	4	0.84%	101	1	0.99%	1.18 [0.02-12.14]	1.000	1.000
EMC1	478	4	0.84%	101	1	0.99%	1.18 [0.02-12.14]	1.000	1.000
EMILIN2	478	4	0.84%	101	1	0.99%	1.18 [0.02-12.14]	1.000	1.000
EMR2	478	4	0.84%	101	1	0.99%	1.18 [0.02-12.14]	1.000	1.000
EPB41L2	478	4	0.84%	101	1	0.99%	1.18 [0.02-12.14]	1.000	1.000
ESPL1	478	4	0.84%	101	1	0.99%	1.18 [0.02-12.14]	1.000	1.000
FAM13C	478	4	0.84%	101	1	0.99%	1.18 [0.02-12.14]	1.000	1.000
FAM22G	478	4	0.84%	101	1	0.99%	1.18 [0.02-12.14]	1.000	1.000
FAM47A	478	4	0.84%	101	1	0.99%	1.18 [0.02-12.14]	1.000	1.000
FBXO18	478	4	0.84%	101	1	0.99%	1.18 [0.02-12.14]	1.000	1.000
FCHO1	478	4	0.84%	101	1	0.99%	1.18 [0.02-12.14]	1.000	1.000
FGFR2	478	4	0.84%	101	1	0.99%	1.18 [0.02-12.14]	1.000	1.000
FHDC1	478	4	0.84%	101	1	0.99%	1.18 [0.02-12.14]	1.000	1.000
FHOD3	478	4	0.84%	101	1	0.99%	1.18 [0.02-12.14]	1.000	1.000
FRMPD2	478	4	0.84%	101	1	0.99%	1.18 [0.02-12.14]	1.000	1.000
FUBP3	478	4	0.84%	101	1	0.99%	1.18 [0.02-12.14]	1.000	1.000
GAP43	478	4	0.84%	101	1	0.99%	1.18 [0.02-12.14]	1.000	1.000
GARNL3	478	4	0.84%	101	1	0.99%	1.18 [0.02-12.14]	1.000	1.000
GLDC	478	4	0.84%	101	1	0.99%	1.18 [0.02-12.14]	1.000	1.000
GPR144	478	4	0.84%	101	1	0.99%	1.18 [0.02-12.14]	1.000	1.000
GPSM2	478	4	0.84%	101	1	0.99%	1.18 [0.02-12.14]	1.000	1.000
GRIA4	478	4	0.84%	101	1	0.99%	1.18 [0.02-12.14]	1.000	1.000
GRIK2	478	4	0.84%	101	1	0.99%	1.18 [0.02-12.14]	1.000	1.000
GRIN3A	478	4	0.84%	101	1	0.99%	1.18 [0.02-12.14]	1.000	1.000
GRM3	478	4	0.84%	101	1	0.99%	1.18 [0.02-12.14]	1.000	1.000
GTF3C1	478	4	0.84%	101	1	0.99%	1.18 [0.02-12.14]	1.000	1.000
HERC2P4	478	4	0.84%	101	1	0.99%	1.18 [0.02-12.14]	1.000	1.000
HKDC1	478	4	0.84%	101	1	0.99%	1.18 [0.02-12.14]	1.000	1.000
HLA-B	478	4	0.84%	101	1	0.99%	1.18 [0.02-12.14]	1.000	1.000
HLA-DRB1	478	4	0.84%	101	1	0.99%	1.18 [0.02-12.14]	1.000	1.000
HLA-DRB5	478	4	0.84%	101	1	0.99%	1.18 [0.02-12.14]	1.000	1.000
HSPD1	478	4	0.84%	101	1	0.99%	1.18 [0.02-12.14]	1.000	1.000
IGFN1	478	4	0.84%	101	1	0.99%	1.18 [0.02-12.14]	1.000	1.000
ILF3	478	4	0.84%	101	1	0.99%	1.18 [0.02-12.14]	1.000	1.000
INADL	478	4	0.84%	101	1	0.99%	1.18 [0.02-12.14]	1.000	1.000
INPP4B	478	4	0.84%	101	1	0.99%	1.18 [0.02-12.14]	1.000	1.000
IREB2	478	4	0.84%	101	1	0.99%	1.18 [0.02-12.14]	1.000	1.000
ITPKB	478	4	0.84%	101	1	0.99%	1.18 [0.02-12.14]	1.000	1.000
KCND2	478	4	0.84%	101	1	0.99%	1.18 [0.02-12.14]	1.000	1.000
KIAA0146	478	4	0.84%	101	1	0.99%	1.18 [0.02-12.14]	1.000	1.000
KIAA0195	478	4	0.84%	101	1	0.99%	1.18 [0.02-12.14]	1.000	1.000
KIAA0319L	478	4	0.84%	101	1	0.99%	1.18 [0.02-12.14]	1.000	1.000
KIAA1239	478	4	0.84%	101	1	0.99%	1.18 [0.02-12.14]	1.000	1.000
KIF15	478	4	0.84%	101	1	0.99%	1.18 [0.02-12.14]	1.000	1.000
KIF27	478	4	0.84%	101	1	0.99%	1.18 [0.02-12.14]	1.000	1.000

Symbol	Luminal			Basal			Basal vs. Luminal		
	N tested	N altered	Fq alteration	N tested	N altered	Fq alteration	OR [95%CI]	p-value*	q-value
KIF2B	478	4	0.84%	101	1	0.99%	1.18 [0.02-12.14]	1.000	1.000
LAMA5	478	4	0.84%	101	1	0.99%	1.18 [0.02-12.14]	1.000	1.000
LDB2	478	4	0.84%	101	1	0.99%	1.18 [0.02-12.14]	1.000	1.000
LEKR1	478	4	0.84%	101	1	0.99%	1.18 [0.02-12.14]	1.000	1.000
LRPPRC	478	4	0.84%	101	1	0.99%	1.18 [0.02-12.14]	1.000	1.000
LRRC7	478	4	0.84%	101	1	0.99%	1.18 [0.02-12.14]	1.000	1.000
LRRC8C	478	4	0.84%	101	1	0.99%	1.18 [0.02-12.14]	1.000	1.000
LUC7L2	478	4	0.84%	101	1	0.99%	1.18 [0.02-12.14]	1.000	1.000
MAST4	478	4	0.84%	101	1	0.99%	1.18 [0.02-12.14]	1.000	1.000
MCF2L	478	4	0.84%	101	1	0.99%	1.18 [0.02-12.14]	1.000	1.000
MCM3AP	478	4	0.84%	101	1	0.99%	1.18 [0.02-12.14]	1.000	1.000
MED12L	478	4	0.84%	101	1	0.99%	1.18 [0.02-12.14]	1.000	1.000
MED14	478	4	0.84%	101	1	0.99%	1.18 [0.02-12.14]	1.000	1.000
MFSD4	478	4	0.84%	101	1	0.99%	1.18 [0.02-12.14]	1.000	1.000
MID1	478	4	0.84%	101	1	0.99%	1.18 [0.02-12.14]	1.000	1.000
MLPH	478	4	0.84%	101	1	0.99%	1.18 [0.02-12.14]	1.000	1.000
MYO16	478	4	0.84%	101	1	0.99%	1.18 [0.02-12.14]	1.000	1.000
MYO6	478	4	0.84%	101	1	0.99%	1.18 [0.02-12.14]	1.000	1.000
MYO7A	478	4	0.84%	101	1	0.99%	1.18 [0.02-12.14]	1.000	1.000
MYO9B	478	4	0.84%	101	1	0.99%	1.18 [0.02-12.14]	1.000	1.000
NAV2	478	4	0.84%	101	1	0.99%	1.18 [0.02-12.14]	1.000	1.000
NBEAL1	478	4	0.84%	101	1	0.99%	1.18 [0.02-12.14]	1.000	1.000
NCKAP1	478	4	0.84%	101	1	0.99%	1.18 [0.02-12.14]	1.000	1.000
NCKAP1L	478	4	0.84%	101	1	0.99%	1.18 [0.02-12.14]	1.000	1.000
NCOR2	478	4	0.84%	101	1	0.99%	1.18 [0.02-12.14]	1.000	1.000
NEK1	478	4	0.84%	101	1	0.99%	1.18 [0.02-12.14]	1.000	1.000
NEK8	478	4	0.84%	101	1	0.99%	1.18 [0.02-12.14]	1.000	1.000
NES	478	4	0.84%	101	1	0.99%	1.18 [0.02-12.14]	1.000	1.000
NID1	478	4	0.84%	101	1	0.99%	1.18 [0.02-12.14]	1.000	1.000
NLGN4X	478	4	0.84%	101	1	0.99%	1.18 [0.02-12.14]	1.000	1.000
NLRP1	478	4	0.84%	101	1	0.99%	1.18 [0.02-12.14]	1.000	1.000
NLRP7	478	4	0.84%	101	1	0.99%	1.18 [0.02-12.14]	1.000	1.000
NOS2	478	4	0.84%	101	1	0.99%	1.18 [0.02-12.14]	1.000	1.000
NRAP	478	4	0.84%	101	1	0.99%	1.18 [0.02-12.14]	1.000	1.000
NUP98	478	4	0.84%	101	1	0.99%	1.18 [0.02-12.14]	1.000	1.000
OR11H1	478	4	0.84%	101	1	0.99%	1.18 [0.02-12.14]	1.000	1.000
OR2W5	478	4	0.84%	101	1	0.99%	1.18 [0.02-12.14]	1.000	1.000
OSBPL8	478	4	0.84%	101	1	0.99%	1.18 [0.02-12.14]	1.000	1.000
PCDHA2	478	4	0.84%	101	1	0.99%	1.18 [0.02-12.14]	1.000	1.000
PCDHA3	478	4	0.84%	101	1	0.99%	1.18 [0.02-12.14]	1.000	1.000
PCDHA5	478	4	0.84%	101	1	0.99%	1.18 [0.02-12.14]	1.000	1.000
PCDHA6	478	4	0.84%	101	1	0.99%	1.18 [0.02-12.14]	1.000	1.000
PCDHAC2	478	4	0.84%	101	1	0.99%	1.18 [0.02-12.14]	1.000	1.000
PCDHGA1	478	4	0.84%	101	1	0.99%	1.18 [0.02-12.14]	1.000	1.000
PDZRN3	478	4	0.84%	101	1	0.99%	1.18 [0.02-12.14]	1.000	1.000
PEAK1	478	4	0.84%	101	1	0.99%	1.18 [0.02-12.14]	1.000	1.000
PHF12	478	4	0.84%	101	1	0.99%	1.18 [0.02-12.14]	1.000	1.000
PHRF1	478	4	0.84%	101	1	0.99%	1.18 [0.02-12.14]	1.000	1.000
PIK3R4	478	4	0.84%	101	1	0.99%	1.18 [0.02-12.14]	1.000	1.000
PLD5	478	4	0.84%	101	1	0.99%	1.18 [0.02-12.14]	1.000	1.000
PLEKHH1	478	4	0.84%	101	1	0.99%	1.18 [0.02-12.14]	1.000	1.000
PLK2	478	4	0.84%	101	1	0.99%	1.18 [0.02-12.14]	1.000	1.000
POLQ	478	4	0.84%	101	1	0.99%	1.18 [0.02-12.14]	1.000	1.000
POM121	478	4	0.84%	101	1	0.99%	1.18 [0.02-12.14]	1.000	1.000
POTEE	478	4	0.84%	101	1	0.99%	1.18 [0.02-12.14]	1.000	1.000
PPARG	478	4	0.84%	101	1	0.99%	1.18 [0.02-12.14]	1.000	1.000
PPP1R1A	478	4	0.84%	101	1	0.99%	1.18 [0.02-12.14]	1.000	1.000
PRICKLE2	478	4	0.84%	101	1	0.99%	1.18 [0.02-12.14]	1.000	1.000
PRKD2	478	4	0.84%	101	1	0.99%	1.18 [0.02-12.14]	1.000	1.000
PROX1	478	4	0.84%	101	1	0.99%	1.18 [0.02-12.14]	1.000	1.000
PTHLH	478	4	0.84%	101	1	0.99%	1.18 [0.02-12.14]	1.000	1.000
RAD54B	478	4	0.84%	101	1	0.99%	1.18 [0.02-12.14]	1.000	1.000
RASAL2	478	4	0.84%	101	1	0.99%	1.18 [0.02-12.14]	1.000	1.000
RBM20	478	4	0.84%	101	1	0.99%	1.18 [0.02-12.14]	1.000	1.000
RBP3	478	4	0.84%	101	1	0.99%	1.18 [0.02-12.14]	1.000	1.000
RFX6	478	4	0.84%	101	1	0.99%	1.18 [0.02-12.14]	1.000	1.000
RHBDF2	478	4	0.84%	101	1	0.99%	1.18 [0.02-12.14]	1.000	1.000
RPS6KA1	478	4	0.84%	101	1	0.99%	1.18 [0.02-12.14]	1.000	1.000
RTN4	478	4	0.84%	101	1	0.99%	1.18 [0.02-12.14]	1.000	1.000
SCN7A	478	4	0.84%	101	1	0.99%	1.18 [0.02-12.14]	1.000	1.000
SDHAP2	478	4	0.84%	101	1	0.99%	1.18 [0.02-12.14]	1.000	1.000
SEMA6A	478	4	0.84%	101	1	0.99%	1.18 [0.02-12.14]	1.000	1.000
SETDB1	478	4	0.84%	101	1	0.99%	1.18 [0.02-12.14]	1.000	1.000
SGSM1	478	4	0.84%	101	1	0.99%	1.18 [0.02-12.14]	1.000	1.000
SH3PXD2A	478	4	0.84%	101	1	0.99%	1.18 [0.02-12.14]	1.000	1.000
SHANK1	478	4	0.84%	101	1	0.99%	1.18 [0.02-12.14]	1.000	1.000
SIRPG	478	4	0.84%	101	1	0.99%	1.18 [0.02-12.14]	1.000	1.000
SLC13A3	478	4	0.84%	101	1	0.99%	1.18 [0.02-12.14]	1.000	1.000
SLC24A4	478	4	0.84%	101	1	0.99%	1.18 [0.02-12.14]	1.000	1.000
SLC4A2	478	4	0.84%	101	1	0.99%	1.18 [0.02-12.14]	1.000	1.000
SLCO1B3	478	4	0.84%	101	1	0.99%	1.18 [0.02-12.14]	1.000	1.000
SLITRK4	478	4	0.84%	101	1	0.99%	1.18 [0.02-12.14]	1.000	1.000
SMARCA1	478	4	0.84%	101	1	0.99%	1.18 [0.02-12.14]	1.000	1.000
SNRK	478	4	0.84%	101	1	0.99%	1.18 [0.02-12.14]	1.000	1.000
SORCS3	478	4	0.84%	101	1	0.99%	1.18 [0.02-12.14]	1.000	1.000
SPAG5	478	4	0.84%	101	1	0.99%	1.18 [0.02-12.14]	1.000	1.000
SRGAP2	478	4	0.84%	101	1	0.99%	1.18 [0.02-12.14]	1.000	1.000
STAB2	478	4	0.84%	101	1	0.99%	1.18 [0.02-12.14]	1.000	1.000
SUZ12	478	4	0.84%	101	1	0.99%	1.18 [0.02-12.14]	1.000	1.000
SZT2	478	4	0.84%	101	1	0.99%	1.18 [0.02-12.14]	1.000	1.000
TCEB3	478	4	0.84%	101	1	0.99%	1.18 [0.02-12.14]	1.000	1.000
TDRD6	478	4	0.84%	101	1	0.99%	1.18 [0.02-12.14]	1.000	1.000
TECTA	478	4	0.84%	101	1	0.99%	1.18 [0.02-12.14]	1.000	1.000
TES	478	4	0.84%	101	1	0.99%	1.18 [0.02-12.14]	1.000	1.000
THADA	478	4	0.84%	101	1	0.99%	1.18 [0.02-12.14]	1.000	1.000
THBS1	478	4	0.84%	101	1	0.99%	1.18 [0.02-12.14]	1.000	1.000
THEM5	478	4	0.84%	101	1	0.99%	1.18 [0.02-12.14]	1.000	1.000
TLL2	478	4	0.84%	101	1	0.99%	1.18 [0.02-12.14]	1.000	1.000
TOPBP1	478	4	0.84%	101	1	0.99%	1.18 [0.02-12.14]	1.000	1.000
TPTE2	478	4	0.84%	101	1	0.99%	1.18 [0.02-12.14]	1.000	1.000
TRIO	478	4	0.84%	101	1	0.99%	1.18 [0.02-12.14]	1.000	1.000
TTC21A	478	4	0.84%	101	1	0.99%	1.18 [0.02-12.14]	1.000	1.000
UBR1	478	4	0.84%	101	1	0.99%	1.18 [0.02-12.14]	1.000	1.000
UBR5	478	4	0.84%	101	1	0.99%	1.18 [0.02-12.14]	1.000	1.000
UNC80	478	4	0.84%	101	1	0.99%	1.18 [0.02-12.14]	1.000	1.000
URB2	478	4	0.84%	101	1	0.99%	1.18 [0.02-12.14]	1.000	1.000
USP25	478	4	0.84%	101	1	0.99%	1.18 [0.02-12.14]	1.000	1.000
VPS41	478	4	0.84%	101	1	0.99%	1.18 [0.02-12.14]	1.000	1.000
VWF	478	4	0.84%	101	1	0.99%	1.18 [0.02-12.14]	1.000	1.000
WDFY3	478	4	0.84%	101	1	0.99%	1.18 [0.02-12.14]	1.000	1.000
WRN	478	4	0.84%	101	1	0.99%	1.18 [0.02-12.14]	1.000	1.000
WWC1	478	4	0.84%	101	1	0.99%	1.18 [0.02-12.14]	1.000	1.000
ZC3H6	478	4	0.84%	101	1	0.99%	1.18 [0.02-12.14]	1.000	1.000
ZFC3H1	478	4	0.84%	101	1	0.99%	1.18 [0.02-12.14]	1.000	1.000
ZMYND8	478	4	0.84%	101	1	0.99%	1.18 [0.02-12.14]	1.000	1.000
ZNF142	478	4	0.84%	101	1	0.99%	1.18 [0.02-12.14]	1.000	1.000
ZNF41	478	4	0.84%	101	1	0.99%	1.18 [0.02-12.14]	1.000	1.000

Symbol	Luminal			Basal			Basal vs. Luminal		
	N tested	N altered	Fq alteration	N tested	N altered	Fq alteration	OR [95%CI]	p-value*	q-value
ZNF536	478	4	0.84%	101	1	0.99%	1.18 [0.02-12.14]	1.000	1.000
ZNF804B	478	4	0.84%	101	1	0.99%	1.18 [0.02-12.14]	1.000	1.000
ZNF90	478	4	0.84%	101	1	0.99%	1.18 [0.02-12.14]	1.000	1.000
ATG9B	478	8	1.67%	101	0	0.00%	0 [0-2.77]	0.362	1.000
CNTNAP3B	478	8	1.67%	101	0	0.00%	0 [0-2.77]	0.362	1.000
EPG5	478	8	1.67%	101	0	0.00%	0 [0-2.77]	0.362	1.000
HERC1	478	8	1.67%	101	0	0.00%	0 [0-2.77]	0.362	1.000
KIF1A	478	8	1.67%	101	0	0.00%	0 [0-2.77]	0.362	1.000
LRP4	478	8	1.67%	101	0	0.00%	0 [0-2.77]	0.362	1.000
MYH8	478	8	1.67%	101	0	0.00%	0 [0-2.77]	0.362	1.000
MYT1L	478	8	1.67%	101	0	0.00%	0 [0-2.77]	0.362	1.000
NRK	478	8	1.67%	101	0	0.00%	0 [0-2.77]	0.362	1.000
RP1L1	478	8	1.67%	101	0	0.00%	0 [0-2.77]	0.362	1.000
TBC1D3P5	478	8	1.67%	101	0	0.00%	0 [0-2.77]	0.362	1.000
ABCA7	478	7	1.46%	101	0	0.00%	0 [0-3.29]	0.612	1.000
C9orf43	478	7	1.46%	101	0	0.00%	0 [0-3.29]	0.612	1.000
CATSPER2	478	7	1.46%	101	0	0.00%	0 [0-3.29]	0.612	1.000
CDKN1B	478	7	1.46%	101	0	0.00%	0 [0-3.29]	0.612	1.000
CECR2	478	7	1.46%	101	0	0.00%	0 [0-3.29]	0.612	1.000
CENPE	478	7	1.46%	101	0	0.00%	0 [0-3.29]	0.612	1.000
CSMD3	478	7	1.46%	101	0	0.00%	0 [0-3.29]	0.612	1.000
DEPDC5	478	7	1.46%	101	0	0.00%	0 [0-3.29]	0.612	1.000
DNAH6	478	7	1.46%	101	0	0.00%	0 [0-3.29]	0.612	1.000
DOCK7	478	7	1.46%	101	0	0.00%	0 [0-3.29]	0.612	1.000
ENSG00000226232	478	7	1.46%	101	0	0.00%	0 [0-3.29]	0.612	1.000
FSIP2	478	7	1.46%	101	0	0.00%	0 [0-3.29]	0.612	1.000
JAK1	478	7	1.46%	101	0	0.00%	0 [0-3.29]	0.612	1.000
KIAA0430	478	7	1.46%	101	0	0.00%	0 [0-3.29]	0.612	1.000
MAP3K13	478	7	1.46%	101	0	0.00%	0 [0-3.29]	0.612	1.000
NAV1	478	7	1.46%	101	0	0.00%	0 [0-3.29]	0.612	1.000
NAV3	478	7	1.46%	101	0	0.00%	0 [0-3.29]	0.612	1.000
ODZ4	478	7	1.46%	101	0	0.00%	0 [0-3.29]	0.612	1.000
PKHD1	478	7	1.46%	101	0	0.00%	0 [0-3.29]	0.612	1.000
PRRC2C	478	7	1.46%	101	0	0.00%	0 [0-3.29]	0.612	1.000
PTCH1	478	7	1.46%	101	0	0.00%	0 [0-3.29]	0.612	1.000
RERE	478	7	1.46%	101	0	0.00%	0 [0-3.29]	0.612	1.000
RP1	478	7	1.46%	101	0	0.00%	0 [0-3.29]	0.612	1.000
SMCHD1	478	7	1.46%	101	0	0.00%	0 [0-3.29]	0.612	1.000
SPATA13	478	7	1.46%	101	0	0.00%	0 [0-3.29]	0.612	1.000
SPEG	478	7	1.46%	101	0	0.00%	0 [0-3.29]	0.612	1.000
SPHKAP	478	7	1.46%	101	0	0.00%	0 [0-3.29]	0.612	1.000
SUPT6H	478	7	1.46%	101	0	0.00%	0 [0-3.29]	0.612	1.000
TRIP11	478	7	1.46%	101	0	0.00%	0 [0-3.29]	0.612	1.000
TRPM3	478	7	1.46%	101	0	0.00%	0 [0-3.29]	0.612	1.000
VCAN	478	7	1.46%	101	0	0.00%	0 [0-3.29]	0.612	1.000
VPS13B	478	7	1.46%	101	0	0.00%	0 [0-3.29]	0.612	1.000
AFF2	478	6	1.26%	101	0	0.00%	0 [0-4.03]	0.597	1.000
ATAD2B	478	6	1.26%	101	0	0.00%	0 [0-4.03]	0.597	1.000
BTNL8	478	6	1.26%	101	0	0.00%	0 [0-4.03]	0.597	1.000
CACHD1	478	6	1.26%	101	0	0.00%	0 [0-4.03]	0.597	1.000
CDC42BPA	478	6	1.26%	101	0	0.00%	0 [0-4.03]	0.597	1.000
CNTNAP2	478	6	1.26%	101	0	0.00%	0 [0-4.03]	0.597	1.000
COL24A1	478	6	1.26%	101	0	0.00%	0 [0-4.03]	0.597	1.000
COL27A1	478	6	1.26%	101	0	0.00%	0 [0-4.03]	0.597	1.000
COL4A1	478	6	1.26%	101	0	0.00%	0 [0-4.03]	0.597	1.000
CR1	478	6	1.26%	101	0	0.00%	0 [0-4.03]	0.597	1.000
CRAT	478	6	1.26%	101	0	0.00%	0 [0-4.03]	0.597	1.000
CRIPAK	478	6	1.26%	101	0	0.00%	0 [0-4.03]	0.597	1.000
DDX11	478	6	1.26%	101	0	0.00%	0 [0-4.03]	0.597	1.000
EIF2C2	478	6	1.26%	101	0	0.00%	0 [0-4.03]	0.597	1.000
EPB41	478	6	1.26%	101	0	0.00%	0 [0-4.03]	0.597	1.000
ETV5	478	6	1.26%	101	0	0.00%	0 [0-4.03]	0.597	1.000
FAM179B	478	6	1.26%	101	0	0.00%	0 [0-4.03]	0.597	1.000
FLNB	478	6	1.26%	101	0	0.00%	0 [0-4.03]	0.597	1.000
FNDC1	478	6	1.26%	101	0	0.00%	0 [0-4.03]	0.597	1.000
GLI2	478	6	1.26%	101	0	0.00%	0 [0-4.03]	0.597	1.000
GON4L	478	6	1.26%	101	0	0.00%	0 [0-4.03]	0.597	1.000
GRHL2	478	6	1.26%	101	0	0.00%	0 [0-4.03]	0.597	1.000
HECW2	478	6	1.26%	101	0	0.00%	0 [0-4.03]	0.597	1.000
HERC6	478	6	1.26%	101	0	0.00%	0 [0-4.03]	0.597	1.000
HSPG2	478	6	1.26%	101	0	0.00%	0 [0-4.03]	0.597	1.000
KALRN	478	6	1.26%	101	0	0.00%	0 [0-4.03]	0.597	1.000
KDM5B	478	6	1.26%	101	0	0.00%	0 [0-4.03]	0.597	1.000
LAMB3	478	6	1.26%	101	0	0.00%	0 [0-4.03]	0.597	1.000
LETM1	478	6	1.26%	101	0	0.00%	0 [0-4.03]	0.597	1.000
MAP3K5	478	6	1.26%	101	0	0.00%	0 [0-4.03]	0.597	1.000
MAP4K1	478	6	1.26%	101	0	0.00%	0 [0-4.03]	0.597	1.000
MAST1	478	6	1.26%	101	0	0.00%	0 [0-4.03]	0.597	1.000
NALCN	478	6	1.26%	101	0	0.00%	0 [0-4.03]	0.597	1.000
NHS	478	6	1.26%	101	0	0.00%	0 [0-4.03]	0.597	1.000
NR3C2	478	6	1.26%	101	0	0.00%	0 [0-4.03]	0.597	1.000
OR2T33	478	6	1.26%	101	0	0.00%	0 [0-4.03]	0.597	1.000
OR6C76	478	6	1.26%	101	0	0.00%	0 [0-4.03]	0.597	1.000
PBX2P1	478	6	1.26%	101	0	0.00%	0 [0-4.03]	0.597	1.000
PCDH9	478	6	1.26%	101	0	0.00%	0 [0-4.03]	0.597	1.000
PHLDB2	478	6	1.26%	101	0	0.00%	0 [0-4.03]	0.597	1.000
PLEKHG1	478	6	1.26%	101	0	0.00%	0 [0-4.03]	0.597	1.000
POLR1A	478	6	1.26%	101	0	0.00%	0 [0-4.03]	0.597	1.000
PRRC2B	478	6	1.26%	101	0	0.00%	0 [0-4.03]	0.597	1.000
RPLP0P2	478	6	1.26%	101	0	0.00%	0 [0-4.03]	0.597	1.000
SBF2	478	6	1.26%	101	0	0.00%	0 [0-4.03]	0.597	1.000
SCN9A	478	6	1.26%	101	0	0.00%	0 [0-4.03]	0.597	1.000
SDK1	478	6	1.26%	101	0	0.00%	0 [0-4.03]	0.597	1.000
SETD2	478	6	1.26%	101	0	0.00%	0 [0-4.03]	0.597	1.000
THOC2	478	6	1.26%	101	0	0.00%	0 [0-4.03]	0.597	1.000
TTLL5	478	6	1.26%	101	0	0.00%	0 [0-4.03]	0.597	1.000
UBC	478	6	1.26%	101	0	0.00%	0 [0-4.03]	0.597	1.000
UBE4A	478	6	1.26%	101	0	0.00%	0 [0-4.03]	0.597	1.000
UGGT2	478	6	1.26%	101	0	0.00%	0 [0-4.03]	0.597	1.000
USH1C	478	6	1.26%	101	0	0.00%	0 [0-4.03]	0.597	1.000
VWDE	478	6	1.26%	101	0	0.00%	0 [0-4.03]	0.597	1.000
WDR6	478	6	1.26%	101	0	0.00%	0 [0-4.03]	0.597	1.000
WDR66	478	6	1.26%	101	0	0.00%	0 [0-4.03]	0.597	1.000
WNK1	478	6	1.26%	101	0	0.00%	0 [0-4.03]	0.597	1.000
YEATS2	478	6	1.26%	101	0	0.00%	0 [0-4.03]	0.597	1.000
ZFP64	478	6	1.26%	101	0	0.00%	0 [0-4.03]	0.597	1.000
ZNF407	478	6	1.26%	101	0	0.00%	0 [0-4.03]	0.597	1.000
ZNF814	478	6	1.26%	101	0	0.00%	0 [0-4.03]	0.597	1.000
ABCB11	478	5	1.05%	101	0	0.00%	0 [0-5.19]	0.593	1.000
ABR	478	5	1.05%	101	0	0.00%	0 [0-5.19]	0.593	1.000
ADAM29	478	5	1.05%	101	0	0.00%	0 [0-5.19]	0.593	1.000
AFF3	478	5	1.05%	101	0	0.00%	0 [0-5.19]	0.593	1.000
AIM1	478	5	1.05%	101	0	0.00%	0 [0-5.19]	0.593	1.000
ANK1	478	5	1.05%	101	0	0.00%	0 [0-5.19]	0.593	1.000
ANKRD11	478	5	1.05%	101	0	0.00%	0 [0-5.19]	0.593	1.000
ANKRD20A4	478	5	1.05%	101	0	0.00%	0 [0-5.19]	0.593	1.000
AP3B1	478	5	1.05%	101	0	0.00%	0 [0-5.19]	0.593	1.000

Symbol	Luminal			Basal			Basal vs. Luminal		
	N tested	N altered	Fq alteration	N tested	N altered	Fq alteration	OR [95%CI]	p-value*	q-value
APC	478	5	1.05%	101	0	0.00%	0 [0-5.19]	0.593	1.000
ARID1B	478	5	1.05%	101	0	0.00%	0 [0-5.19]	0.593	1.000
ASB10	478	5	1.05%	101	0	0.00%	0 [0-5.19]	0.593	1.000
ATP2A2	478	5	1.05%	101	0	0.00%	0 [0-5.19]	0.593	1.000
ATP2B3	478	5	1.05%	101	0	0.00%	0 [0-5.19]	0.593	1.000
ATP8B2	478	5	1.05%	101	0	0.00%	0 [0-5.19]	0.593	1.000
BCL9L	478	5	1.05%	101	0	0.00%	0 [0-5.19]	0.593	1.000
BRAP	478	5	1.05%	101	0	0.00%	0 [0-5.19]	0.593	1.000
C3	478	5	1.05%	101	0	0.00%	0 [0-5.19]	0.593	1.000
CASC4	478	5	1.05%	101	0	0.00%	0 [0-5.19]	0.593	1.000
CAST	478	5	1.05%	101	0	0.00%	0 [0-5.19]	0.593	1.000
CCDC144A	478	5	1.05%	101	0	0.00%	0 [0-5.19]	0.593	1.000
CCDC57	478	5	1.05%	101	0	0.00%	0 [0-5.19]	0.593	1.000
CCDC88A	478	5	1.05%	101	0	0.00%	0 [0-5.19]	0.593	1.000
CCDC88C	478	5	1.05%	101	0	0.00%	0 [0-5.19]	0.593	1.000
COBLL1	478	5	1.05%	101	0	0.00%	0 [0-5.19]	0.593	1.000
CYP2D7P1	478	5	1.05%	101	0	0.00%	0 [0-5.19]	0.593	1.000
DCAF6	478	5	1.05%	101	0	0.00%	0 [0-5.19]	0.593	1.000
DDR2	478	5	1.05%	101	0	0.00%	0 [0-5.19]	0.593	1.000
DENND1B	478	5	1.05%	101	0	0.00%	0 [0-5.19]	0.593	1.000
DNAJC11	478	5	1.05%	101	0	0.00%	0 [0-5.19]	0.593	1.000
DUOX1	478	5	1.05%	101	0	0.00%	0 [0-5.19]	0.593	1.000
EFCAB6	478	5	1.05%	101	0	0.00%	0 [0-5.19]	0.593	1.000
EFTUD2	478	5	1.05%	101	0	0.00%	0 [0-5.19]	0.593	1.000
EIF4G3	478	5	1.05%	101	0	0.00%	0 [0-5.19]	0.593	1.000
ELAC2	478	5	1.05%	101	0	0.00%	0 [0-5.19]	0.593	1.000
EML2	478	5	1.05%	101	0	0.00%	0 [0-5.19]	0.593	1.000
FAM86B2	478	5	1.05%	101	0	0.00%	0 [0-5.19]	0.593	1.000
FASN	478	5	1.05%	101	0	0.00%	0 [0-5.19]	0.593	1.000
FMN1	478	5	1.05%	101	0	0.00%	0 [0-5.19]	0.593	1.000
FN1	478	5	1.05%	101	0	0.00%	0 [0-5.19]	0.593	1.000
FOLH1	478	5	1.05%	101	0	0.00%	0 [0-5.19]	0.593	1.000
FRYL	478	5	1.05%	101	0	0.00%	0 [0-5.19]	0.593	1.000
GGT3P	478	5	1.05%	101	0	0.00%	0 [0-5.19]	0.593	1.000
GLB1L3	478	5	1.05%	101	0	0.00%	0 [0-5.19]	0.593	1.000
GPR116	478	5	1.05%	101	0	0.00%	0 [0-5.19]	0.593	1.000
GPRIN2	478	5	1.05%	101	0	0.00%	0 [0-5.19]	0.593	1.000
GUSBP11	478	5	1.05%	101	0	0.00%	0 [0-5.19]	0.593	1.000
GYG2	478	5	1.05%	101	0	0.00%	0 [0-5.19]	0.593	1.000
GYS1	478	5	1.05%	101	0	0.00%	0 [0-5.19]	0.593	1.000
HDLBP	478	5	1.05%	101	0	0.00%	0 [0-5.19]	0.593	1.000
HIVEP2	478	5	1.05%	101	0	0.00%	0 [0-5.19]	0.593	1.000
HLA-J	478	5	1.05%	101	0	0.00%	0 [0-5.19]	0.593	1.000
HNRNPR	478	5	1.05%	101	0	0.00%	0 [0-5.19]	0.593	1.000
IKBKAP	478	5	1.05%	101	0	0.00%	0 [0-5.19]	0.593	1.000
INTS8	478	5	1.05%	101	0	0.00%	0 [0-5.19]	0.593	1.000
ITGB2	478	5	1.05%	101	0	0.00%	0 [0-5.19]	0.593	1.000
KCNB2	478	5	1.05%	101	0	0.00%	0 [0-5.19]	0.593	1.000
KCTD3	478	5	1.05%	101	0	0.00%	0 [0-5.19]	0.593	1.000
KDM2A	478	5	1.05%	101	0	0.00%	0 [0-5.19]	0.593	1.000
KIAA2022	478	5	1.05%	101	0	0.00%	0 [0-5.19]	0.593	1.000
KIF4A	478	5	1.05%	101	0	0.00%	0 [0-5.19]	0.593	1.000
KIT	478	5	1.05%	101	0	0.00%	0 [0-5.19]	0.593	1.000
KNTC1	478	5	1.05%	101	0	0.00%	0 [0-5.19]	0.593	1.000
LAMA3	478	5	1.05%	101	0	0.00%	0 [0-5.19]	0.593	1.000
LIMA1	478	5	1.05%	101	0	0.00%	0 [0-5.19]	0.593	1.000
LPHN2	478	5	1.05%	101	0	0.00%	0 [0-5.19]	0.593	1.000
LRIG2	478	5	1.05%	101	0	0.00%	0 [0-5.19]	0.593	1.000
MAGEC3	478	5	1.05%	101	0	0.00%	0 [0-5.19]	0.593	1.000
MKI67	478	5	1.05%	101	0	0.00%	0 [0-5.19]	0.593	1.000
MYH2	478	5	1.05%	101	0	0.00%	0 [0-5.19]	0.593	1.000
MYO15A	478	5	1.05%	101	0	0.00%	0 [0-5.19]	0.593	1.000
MYO18B	478	5	1.05%	101	0	0.00%	0 [0-5.19]	0.593	1.000
MYO3A	478	5	1.05%	101	0	0.00%	0 [0-5.19]	0.593	1.000
NBEAL2	478	5	1.05%	101	0	0.00%	0 [0-5.19]	0.593	1.000
NEK5	478	5	1.05%	101	0	0.00%	0 [0-5.19]	0.593	1.000
NEURL4	478	5	1.05%	101	0	0.00%	0 [0-5.19]	0.593	1.000
NFATC1	478	5	1.05%	101	0	0.00%	0 [0-5.19]	0.593	1.000
NRP2	478	5	1.05%	101	0	0.00%	0 [0-5.19]	0.593	1.000
NUMA1	478	5	1.05%	101	0	0.00%	0 [0-5.19]	0.593	1.000
PANK4	478	5	1.05%	101	0	0.00%	0 [0-5.19]	0.593	1.000
PCDH1	478	5	1.05%	101	0	0.00%	0 [0-5.19]	0.593	1.000
PCDH7	478	5	1.05%	101	0	0.00%	0 [0-5.19]	0.593	1.000
PCDHA1	478	5	1.05%	101	0	0.00%	0 [0-5.19]	0.593	1.000
PCDHB11	478	5	1.05%	101	0	0.00%	0 [0-5.19]	0.593	1.000
PCDHGB7	478	5	1.05%	101	0	0.00%	0 [0-5.19]	0.593	1.000
PGAP1	478	5	1.05%	101	0	0.00%	0 [0-5.19]	0.593	1.000
PHF3	478	5	1.05%	101	0	0.00%	0 [0-5.19]	0.593	1.000
PIK3C2B	478	5	1.05%	101	0	0.00%	0 [0-5.19]	0.593	1.000
PLA2R1	478	5	1.05%	101	0	0.00%	0 [0-5.19]	0.593	1.000
PLCB2	478	5	1.05%	101	0	0.00%	0 [0-5.19]	0.593	1.000
POLR2B	478	5	1.05%	101	0	0.00%	0 [0-5.19]	0.593	1.000
PRAMEF4	478	5	1.05%	101	0	0.00%	0 [0-5.19]	0.593	1.000
PRKRIR	478	5	1.05%	101	0	0.00%	0 [0-5.19]	0.593	1.000
PTCHD2	478	5	1.05%	101	0	0.00%	0 [0-5.19]	0.593	1.000
PTPN13	478	5	1.05%	101	0	0.00%	0 [0-5.19]	0.593	1.000
PTPN14	478	5	1.05%	101	0	0.00%	0 [0-5.19]	0.593	1.000
PTPRE	478	5	1.05%	101	0	0.00%	0 [0-5.19]	0.593	1.000
PXDN	478	5	1.05%	101	0	0.00%	0 [0-5.19]	0.593	1.000
RARS	478	5	1.05%	101	0	0.00%	0 [0-5.19]	0.593	1.000
RB1CC1	478	5	1.05%	101	0	0.00%	0 [0-5.19]	0.593	1.000
RIF1	478	5	1.05%	101	0	0.00%	0 [0-5.19]	0.593	1.000
SBF1	478	5	1.05%	101	0	0.00%	0 [0-5.19]	0.593	1.000
SCN4A	478	5	1.05%	101	0	0.00%	0 [0-5.19]	0.593	1.000
SFMBT2	478	5	1.05%	101	0	0.00%	0 [0-5.19]	0.593	1.000
SHROOM2	478	5	1.05%	101	0	0.00%	0 [0-5.19]	0.593	1.000
SLC4A4	478	5	1.05%	101	0	0.00%	0 [0-5.19]	0.593	1.000
SMARCC1	478	5	1.05%	101	0	0.00%	0 [0-5.19]	0.593	1.000
SNRNP200	478	5	1.05%	101	0	0.00%	0 [0-5.19]	0.593	1.000
SORCS1	478	5	1.05%	101	0	0.00%	0 [0-5.19]	0.593	1.000
SSC5D	478	5	1.05%	101	0	0.00%	0 [0-5.19]	0.593	1.000
SUPT5H	478	5	1.05%	101	0	0.00%	0 [0-5.19]	0.593	1.000
SYNPO2	478	5	1.05%	101	0	0.00%	0 [0-5.19]	0.593	1.000
TBC1D4	478	5	1.05%	101	0	0.00%	0 [0-5.19]	0.593	1.000
TBC1D9B	478	5	1.05%	101	0	0.00%	0 [0-5.19]	0.593	1.000
TBCD	478	5	1.05%	101	0	0.00%	0 [0-5.19]	0.593	1.000
TET1	478	5	1.05%	101	0	0.00%	0 [0-5.19]	0.593	1.000
TMEM120B	478	5	1.05%	101	0	0.00%	0 [0-5.19]	0.593	1.000
TMEM63A	478	5	1.05%	101	0	0.00%	0 [0-5.19]	0.593	1.000
TNS4	478	5	1.05%	101	0	0.00%	0 [0-5.19]	0.593	1.000
TOX2	478	5	1.05%	101	0	0.00%	0 [0-5.19]	0.593	1.000
TPM2	478	5	1.05%	101	0	0.00%	0 [0-5.19]	0.593	1.000
TPP2	478	5	1.05%	101	0	0.00%	0 [0-5.19]	0.593	1.000
TRPC5	478	5	1.05%	101	0	0.00%	0 [0-5.19]	0.593	1.000
TRPM8	478	5	1.05%	101	0	0.00%	0 [0-5.19]	0.593	1.000
TRPV2	478	5	1.05%	101	0	0.00%	0 [0-5.19]	0.593	1.000
TTC3	478	5	1.05%	101	0	0.00%	0 [0-5.19]	0.593	1.000

Symbol	Luminal			Basal			Basal vs. Luminal		
	N tested	N altered	Fq alteration	N tested	N altered	Fq alteration	OR [95%CI]	p-value*	q-value
TTC7B	478	5	1.05%	101	0	0.00%	0 [0-5.19]	0.593	1.000
TUBBP5	478	5	1.05%	101	0	0.00%	0 [0-5.19]	0.593	1.000
TULP4	478	5	1.05%	101	0	0.00%	0 [0-5.19]	0.593	1.000
USP8	478	5	1.05%	101	0	0.00%	0 [0-5.19]	0.593	1.000
VPS52	478	5	1.05%	101	0	0.00%	0 [0-5.19]	0.593	1.000
WDR17	478	5	1.05%	101	0	0.00%	0 [0-5.19]	0.593	1.000
WHAMMP2	478	5	1.05%	101	0	0.00%	0 [0-5.19]	0.593	1.000
WSCD2	478	5	1.05%	101	0	0.00%	0 [0-5.19]	0.593	1.000
XDH	478	5	1.05%	101	0	0.00%	0 [0-5.19]	0.593	1.000
ZDHHC11B	478	5	1.05%	101	0	0.00%	0 [0-5.19]	0.593	1.000
ZFP36L1	478	5	1.05%	101	0	0.00%	0 [0-5.19]	0.593	1.000
ZNF397	478	5	1.05%	101	0	0.00%	0 [0-5.19]	0.593	1.000

*, logistic regression

Supplementary Table 5: List of 906 genes differentially expressed between the ER+/HER2- Luminal and Basal breast cancers (TCGA)

Symbol	Description	Cytoband	Entrez Gene ID	Basal vs. Luminal (log2-ratio)	t	p-value[1]	q-value	Associated to
PSAT1	phosphoserine aminotransferase 1	9q21.2	29968	2.31	14.19	5.81E-40	6.28E-38	up Basal vs. Luminal
MTHFD1L	methylenetetrahydrofolate dehydrogenase	6q25.1	25902	1.19	13.88	1.73E-38	1.63E-36	up Basal vs. Luminal
SMCO4	single-pass membrane protein with coiled-c	11q21	56935	1.21	13.85	2.22E-38	1.99E-36	up Basal vs. Luminal
HAPLN3	hyaluronan and proteoglycan link protein 3	15q26.1	145864	1.60	13.32	6.59E-36	4.33E-34	up Basal vs. Luminal
UPP1	uridine phosphorylase 1	7p12.3	7378	1.10	13.24	1.53E-35	9.51E-34	up Basal vs. Luminal
CHST2	carbohydrate sulfotransferase 2	3q24	9435	1.13	12.75	2.36E-33	1.19E-31	up Basal vs. Luminal
GLIPR2	GLI pathogenesis related 2	9p13.3	152007	1.21	12.69	4.22E-33	2.07E-31	up Basal vs. Luminal
FZD9	frizzled class receptor 9	7q11.23	8326	1.64	12.14	1.08E-30	4.25E-29	up Basal vs. Luminal
A2ML1	alpha-2-macroglobulin like 1	12p13.31	144568	2.53	12.09	1.80E-30	6.90E-29	up Basal vs. Luminal
SLC26A9	solute carrier family 26 member 9	1q32.1	115019	1.63	12.02	3.72E-30	1.33E-28	up Basal vs. Luminal
B3GNT7	UDP-GlcNAc:betaGal beta-1,3-N-acetylglu	2q37.1|2q37.1	93010	1.15	11.91	1.04E-29	3.33E-28	up Basal vs. Luminal
ACE2	angiotensin I converting enzyme 2	Xp22.2	59272	1.63	11.79	3.58E-29	1.10E-27	up Basal vs. Luminal
ART3	ADP-ribosyltransferase 3	4q21.1|4p15.1-p14	419	1.86	11.74	5.57E-29	1.69E-27	up Basal vs. Luminal
FSCN1	fascin actin-bundling protein 1	7p22.1	6624	1.04	11.72	6.68E-29	1.98E-27	up Basal vs. Luminal
RARRES1	retinoic acid receptor responder 1	3q25.32	5918	2.04	11.68	9.78E-29	2.72E-27	up Basal vs. Luminal
KCNG1	potassium voltage-gated channel modifier s	20q13.13	3755	2.17	11.56	3.25E-28	8.19E-27	up Basal vs. Luminal
PLTP	phospholipid transfer protein	20q13.12	5360	1.13	11.50	5.91E-28	1.42E-26	up Basal vs. Luminal
GSDMC	gasdermin C	8q24.21	56169	1.80	11.43	1.18E-27	2.72E-26	up Basal vs. Luminal
BATF3	basic leucine zipper ATF-like transcription f	1q32.3	55509	1.14	11.41	1.35E-27	3.06E-26	up Basal vs. Luminal
CDCA7	cell division cycle associated 7	2q31.1	83879	1.83	11.28	4.88E-27	1.06E-25	up Basal vs. Luminal
ITM2C	integral membrane protein 2C	2q37.1	81618	1.13	11.17	1.32E-26	2.68E-25	up Basal vs. Luminal
IGF2BP2	insulin like growth factor 2 mRNA binding p	3q27.2	10644	1.59	11.09	2.84E-26	5.56E-25	up Basal vs. Luminal
ARL4C	ADP ribosylation factor like GTPase 4C	2q37.1	10123	1.11	11.07	3.44E-26	6.66E-25	up Basal vs. Luminal
CSRP2	cysteine and glycine rich protein 2	12q21.2	1466	1.08	11.03	5.05E-26	9.52E-25	up Basal vs. Luminal
C1QL4	complement C1q like 4	12q13.12	338761	1.46	11.02	5.63E-26	1.05E-24	up Basal vs. Luminal
FOXC1	forkhead box C1	6p25.3	2296	1.44	10.99	7.19E-26	1.32E-24	up Basal vs. Luminal
FAM20A	FAM20A golgi associated secretory pathwa	17q24.2	54757	1.27	10.92	1.44E-25	2.50E-24	up Basal vs. Luminal
SLC15A1	solute carrier family 15 member 1	13q32.2-q32.3	6564	1.54	10.82	3.54E-25	5.69E-24	up Basal vs. Luminal
EN1	engrailed homeobox 1	2q14.2	2019	1.65	10.79	4.56E-25	7.26E-24	up Basal vs. Luminal
S100A8	S100 calcium binding protein A8	1q21.3	6279	2.41	10.76	5.84E-25	9.05E-24	up Basal vs. Luminal
CRABP1	cellular retinoic acid binding protein 1	15q25.1	1381	3.20	10.74	7.34E-25	1.11E-23	up Basal vs. Luminal
DLX5	distal-less homeobox 5	7q21.3	1749	1.33	10.72	8.65E-25	1.30E-23	up Basal vs. Luminal
INAVA	innate immunity activator	1q32.1	55765	2.19	10.68	1.31E-24	1.88E-23	up Basal vs. Luminal
GABBR2	gamma-aminobutyric acid type B receptor s	9q22.33	9568	1.61	10.63	2.07E-24	2.92E-23	up Basal vs. Luminal
ATP6V1C2	ATPase H+ transporting V1 subunit C2	2p25.1	245973	1.06	10.62	2.27E-24	3.18E-23	up Basal vs. Luminal
CBX2	chromobox 2	17q25.3	84733	1.56	10.59	2.84E-24	3.94E-23	up Basal vs. Luminal
VASN	vasorin	16p13.3	114990	1.01	10.57	3.49E-24	4.75E-23	up Basal vs. Luminal
TRPM8	transient receptor potential cation channel s	2q37.1	79054	1.34	10.57	3.50E-24	4.75E-23	up Basal vs. Luminal
CIB2	calcium and integrin binding family membe	15q25.1	10518	1.27	10.52	5.41E-24	7.18E-23	up Basal vs. Luminal
ULBP2	UL16 binding protein 2	6q25.1	80328	1.28	10.52	5.51E-24	7.26E-23	up Basal vs. Luminal
NXN	nucleoredoxin	17p13.3	64359	1.07	10.51	5.73E-24	7.52E-23	up Basal vs. Luminal
SRSF12	serine and arginine rich splicing factor 12	6q15	135295	1.20	10.48	7.83E-24	9.89E-23	up Basal vs. Luminal
EPHB6	EPH receptor B6	7q34	2051	1.56	10.45	9.85E-24	1.23E-22	up Basal vs. Luminal
PIWIL4	piwi like RNA-mediated gene silencing 4	11q21	143689	1.09	10.44	1.11E-23	1.36E-22	up Basal vs. Luminal
TNIP3	TNFAIP3 interacting protein 3	4q27	79931	1.31	10.43	1.26E-23	1.53E-22	up Basal vs. Luminal
P2RY6	pyrimidinergic receptor P2Y6	11q13.4	5031	1.12	10.40	1.63E-23	1.96E-22	up Basal vs. Luminal
TMEM171	transmembrane protein 171	5q13.2	134285	1.22	10.39	1.81E-23	2.16E-22	up Basal vs. Luminal
WNT6	Wnt family member 6	2q35	7475	1.60	10.38	1.84E-23	2.18E-22	up Basal vs. Luminal
APBA2	amyloid beta precursor protein binding fam	15q13.1	321	1.46	10.35	2.61E-23	3.08E-22	up Basal vs. Luminal
DDN	dendrin	12q13.12	23109	1.15	10.33	2.88E-23	3.37E-22	up Basal vs. Luminal
SLC30A3	solute carrier family 30 member 3	2p23.3	7781	1.08	10.31	3.43E-23	3.98E-22	up Basal vs. Luminal
ABTB2	ankyrin repeat and BTB domain containing	11p13	25841	1.26	10.29	4.26E-23	4.85E-22	up Basal vs. Luminal
CXCL5	C-X-C motif chemokine ligand 5	4q13.3	6374	1.58	10.27	5.05E-23	5.69E-22	up Basal vs. Luminal
IL22RA2	interleukin 22 receptor subunit alpha 2	6q23.3	116379	1.44	10.24	6.60E-23	7.22E-22	up Basal vs. Luminal
PLEKHF1	pleckstrin homology and FYVE domain con	19q12	79156	1.15	10.24	6.89E-23	7.52E-22	up Basal vs. Luminal
ADM	adrenomedullin	11p15.4	133	1.39	10.21	8.78E-23	9.36E-22	up Basal vs. Luminal
LINC00092	long intergenic non-protein coding RNA 92	9q22.32	100188953	1.28	10.18	1.15E-22	1.22E-21	up Basal vs. Luminal
CYP27C1	cytochrome P450 family 27 subfamily C me	2q14.3	339761	1.25	10.15	1.51E-22	1.56E-21	up Basal vs. Luminal
RASGEF1C	RasGEF domain family member 1C	5q35.3	255426	1.19	10.11	2.05E-22	2.09E-21	up Basal vs. Luminal
GAL	galanin and GMAP prepropeptide	11q13.2	51083	1.75	10.08	2.82E-22	2.84E-21	up Basal vs. Luminal
FAM171A1	family with sequence similarity 171 membe	10p13	221061	1.08	10.06	3.36E-22	3.36E-21	up Basal vs. Luminal
NFIL3	nuclear factor, interleukin 3 regulated	9q22.31	4783	1.08	10.05	3.51E-22	3.48E-21	up Basal vs. Luminal
LMO4	LIM domain only 4	1p22.3	8543	1.03	9.96	7.83E-22	7.30E-21	up Basal vs. Luminal
KCNN4	potassium calcium-activated channel subfa	19q13.31	3783	1.47	9.95	8.45E-22	7.82E-21	up Basal vs. Luminal
LDHB	lactate dehydrogenase B	12p12.1	3945	1.10	9.94	9.16E-22	8.42E-21	up Basal vs. Luminal
RUNX3	runt related transcription factor 3	1p36.11	864	1.20	9.91	1.19E-21	1.07E-20	up Basal vs. Luminal
S100A9	S100 calcium binding protein A9	1q21.3	6280	2.38	9.90	1.31E-21	1.17E-20	up Basal vs. Luminal
CHODL	chondrolectin	21q21.1	140578	1.42	9.86	1.94E-21	1.68E-20	up Basal vs. Luminal
B3GNT5	UDP-GlcNAc:betaGal beta-1,3-N-acetylglu	3q27.1	84002	1.04	9.85	2.06E-21	1.77E-20	up Basal vs. Luminal
GALNT12	polypeptide N-acetylgalactosaminyltransfer	9q22.33	79695	1.17	9.81	2.82E-21	2.39E-20	up Basal vs. Luminal
PNMA3	PNMA family member 3	Xq28	29944	1.51	9.79	3.30E-21	2.75E-20	up Basal vs. Luminal
MATN4	matrilin 4	20q13.12	8785	1.11	9.79	3.52E-21	2.93E-20	up Basal vs. Luminal
SH2D2A	SH2 domain containing 2A	1q23.1	9047	1.38	9.77	4.20E-21	3.45E-20	up Basal vs. Luminal
TMEM158	transmembrane protein 158 (gene/pseudog	3p21.31	25907	1.41	9.76	4.29E-21	3.51E-20	up Basal vs. Luminal
BCL2A1	BCL2 related protein A1	15q25.1	597	1.32	9.76	4.36E-21	3.55E-20	up Basal vs. Luminal
VGLL1	vestigial like family member 1	Xq26.3	51442	2.07	9.75	4.93E-21	3.98E-20	up Basal vs. Luminal
C1QL2	complement C1q like 2	2q14.2	165257	1.23	9.74	5.47E-21	4.34E-20	up Basal vs. Luminal
PI3	peptidase inhibitor 3	20q13.12	5266	2.09	9.71	7.00E-21	5.49E-20	up Basal vs. Luminal
IL1R2	interleukin 1 receptor type 2	2q11.2	7850	1.46	9.70	7.19E-21	5.60E-20	up Basal vs. Luminal
IL4I1	interleukin 4 induced 1	19q13.33	259307	1.26	9.68	8.85E-21	6.79E-20	up Basal vs. Luminal
B3GNT4	UDP-GlcNAc:betaGal beta-1,3-N-acetylglu	12q24.31	79369	1.16	9.67	9.44E-21	7.23E-20	up Basal vs. Luminal
ANKS6	ankyrin repeat and sterile alpha motif doma	9q22.33	203286	1.10	9.66	1.07E-20	8.18E-20	up Basal vs. Luminal
CT83	cancer/testis antigen 83	Xq23	203413	1.13	9.63	1.36E-20	1.02E-19	up Basal vs. Luminal
CCL18	C-C motif chemokine ligand 18	17q12	6362	2.02	9.62	1.46E-20	1.09E-19	up Basal vs. Luminal
CCL13	C-C motif chemokine ligand 13	17q12	6357	1.69	9.60	1.78E-20	1.31E-19	up Basal vs. Luminal
MFSD2A	major facilitator superfamily domain contair	1p34.2	84879	1.30	9.58	2.08E-20	1.52E-19	up Basal vs. Luminal
ST8SIA1	ST8 alpha-N-acetyl-neuraminide alpha-2,8	12p12.1	6489	1.31	9.57	2.34E-20	1.69E-19	up Basal vs. Luminal
JAK3	Janus kinase 3	19p13.11	3718	1.19	9.56	2.37E-20	1.70E-19	up Basal vs. Luminal
BCL11A	BAF chromatin remodeling complex subuni	2p16.1	53335	1.92	9.53	3.24E-20	2.27E-19	up Basal vs. Luminal
IL12RB2	interleukin 12 receptor subunit beta 2	1p31.3	3595	1.65	9.50	4.18E-20	2.89E-19	up Basal vs. Luminal
MARCO	macrophage receptor with collagenous stru	2q14.2	8685	2.13	9.49	4.55E-20	3.12E-19	up Basal vs. Luminal
LEMD1	LEM domain containing 1	1q32.1	93273	1.17	9.48	4.68E-20	3.19E-19	up Basal vs. Luminal
SLC2A5	solute carrier family 2 member 5	1p36.23	6518	1.29	9.46	5.76E-20	3.88E-19	up Basal vs. Luminal
CPA4	carboxypeptidase A4	7q32.2	51200	1.64	9.43	7.02E-20	4.67E-19	up Basal vs. Luminal
NDRG1	N-myc downstream regulated 1	8q24.22	10397	1.01	9.42	7.96E-20	5.27E-19	up Basal vs. Luminal
LILRA3	leukocyte immunoglobulin like receptor A3	19q13.4	11026	1.07	9.39	1.00E-19	6.49E-19	up Basal vs. Luminal
SBSN	suprabasin	19q13.12	374897	1.79	9.36	1.31E-19	8.38E-19	up Basal vs. Luminal
MT1X	metallothionein 1X	16q13	4501	1.25	9.34	1.56E-19	9.88E-19	up Basal vs. Luminal
SLC2A6	solute carrier family 2 member 6	9q34.2	11182	1.08	9.32	1.88E-19	1.18E-18	up Basal vs. Luminal
MYO1H	myosin IH	12q24.11	283446	1.05	9.32	1.90E-19	1.19E-18	up Basal vs. Luminal
LAG3	lymphocyte activating 3	12p13.31	3902	1.43	9.31	2.01E-19	1.25E-18	up Basal vs. Luminal
CD72	CD72 molecule	9p13.3	971	1.00	9.30	2.09E-19	1.30E-18	up Basal vs. Luminal
AIF1L	allograft inflammatory factor 1 like	9q34.12-q34.13	83543	1.25	9.30	2.11E-19	1.31E-18	up Basal vs. Luminal
COL11A2	collagen type XI alpha 2 chain	6p21.32	1302	1.28	9.29	2.38E-19	1.47E-18	up Basal vs. Luminal
FUOM	fucose mutarotase	10q26.3	282969	1.04	9.29	2.41E-19	1.49E-18	up Basal vs. Luminal
ACP7	acid phosphatase 7, tartrate resistant (puta	19q13.2	390928	1.06	9.28	2.53E-19	1.55E-18	up Basal vs. Luminal
TLR9	toll like receptor 9	3p21.2	54106	1.01	9.28	2.58E-19	1.58E-18	up Basal vs. Luminal
MID1	midline 1	Xp22.2	4281	1.20	9.28	2.63E-19	1.61E-18	up Basal vs. Luminal
GFRA3	GDNF family receptor alpha 3	5q31.2	2676	1.23	9.25	3.40E-19	2.04E-18	up Basal vs. Luminal
AMY1A	amylase alpha 1A (salivary)	1p21.1	276	1.45	9.24	3.53E-19	2.11E-18	up Basal vs. Luminal
PNOC	prepronociceptin	8p21.1	5368	1.43	9.23	4.00E-19	2.37E-18	up Basal vs. Luminal
LINC01554	long intergenic non-protein coding RNA 15	5q15	202299	1.15	9.21	4.37E-19	2.56E-18	up Basal vs. Luminal
KRT86	keratin 86	12q13.13	3892	1.53	9.20	4.96E-19	2.88E-18	up Basal vs. Luminal
RHCG	Rh family C glycoprotein	15q26.1	51458	1.69	9.20	5.00E-19	2.89E-18	up Basal vs. Luminal
RGMA	repulsive guidance molecule BMP co-recep	15q26.1	56963	1.33	9.19	5.31E-19	3.05E-18	up Basal vs. Luminal
RASAL1	RAS protein activator like 1	12q24.13	8437	1.87	9.16	6.69E-19	3.77E-18	up Basal vs. Luminal

Symbol	Description	Cytoband	Entrez Gene ID	Basal vs. Luminal (log2-ratio)	t	p-value[1]	q-value	Associated to
SOCS1	suppressor of cytokine signaling 1	16p13.13	8651	1.16	9.16	6.93E-19	3.90E-18	up Basal vs. Luminal
PFKP	phosphofructokinase, platelet	10p15.2	5214	1.11	9.15	7.30E-19	4.10E-18	up Basal vs. Luminal
IDO1	indoleamine 2,3-dioxygenase 1	8p11.21	3620	1.76	9.15	7.61E-19	4.27E-18	up Basal vs. Luminal
PRSS33	serine protease 33	16p13.3	260429	1.08	9.12	9.40E-19	5.20E-18	up Basal vs. Luminal
GBP1	guanylate binding protein 1	1p22.2	2633	1.10	9.12	9.80E-19	5.41E-18	up Basal vs. Luminal
NXPH4	neurexophilin 4	12q13.3	11247	1.46	9.11	1.01E-18	5.55E-18	up Basal vs. Luminal
SCRG1	stimulator of chondrogenesis 1	4q34.1	11341	1.67	9.11	1.03E-18	5.63E-18	up Basal vs. Luminal
FOSL1	FOS like 1, AP-1 transcription factor subuni	11q13.1	8061	1.15	9.11	1.06E-18	5.82E-18	up Basal vs. Luminal
SPHK1	sphingosine kinase 1	17q25.1	8877	1.01	9.08	1.28E-18	6.89E-18	up Basal vs. Luminal
PPP1R14C	protein phosphatase 1 regulatory inhibitor s	6q25.1	81706	1.79	9.08	1.29E-18	6.96E-18	up Basal vs. Luminal
ARHGEF4	Rho guanine nucleotide exchange factor 4	2q21.1	50649	1.31	9.06	1.53E-18	8.15E-18	up Basal vs. Luminal
KCNK5	potassium two pore domain channel subfar	6p21.2	8645	1.37	9.01	2.33E-18	1.22E-17	up Basal vs. Luminal
GZMB	granzyme B	14q12	3002	1.67	8.95	3.79E-18	1.95E-17	up Basal vs. Luminal
CARD9	caspase recruitment domain family membe	9q34.3	64170	1.18	8.94	4.06E-18	2.07E-17	up Basal vs. Luminal
TNNT2	troponin T2, cardiac type	1q32.1	7139	1.44	8.91	5.01E-18	2.52E-17	up Basal vs. Luminal
HORMAD1	HORMA domain containing 1	1q21.3	84072	1.36	8.88	6.49E-18	3.21E-17	up Basal vs. Luminal
LAD1	ladinin 1	1q32.1	3898	1.50	8.87	6.96E-18	3.42E-17	up Basal vs. Luminal
CLCN4	chloride voltage-gated channel 4	Xp22.2	1183	1.11	8.86	7.62E-18	3.73E-17	up Basal vs. Luminal
XIRP1	xin actin binding repeat containing 1	3p22.2	165904	1.54	8.85	8.57E-18	4.19E-17	up Basal vs. Luminal
PADI2	peptidyl arginine deiminase 2	1p36.13	11240	1.34	8.84	9.34E-18	4.54E-17	up Basal vs. Luminal
MPP6	membrane palmitoylated protein 6	7p15.3	51678	1.17	8.81	1.17E-17	5.54E-17	up Basal vs. Luminal
B3GAT1	beta-1,3-glucuronyltransferase 1	11q25	27087	1.58	8.80	1.25E-17	5.94E-17	up Basal vs. Luminal
MSLN	mesothelin	16p13.3	10232	1.99	8.78	1.42E-17	6.65E-17	up Basal vs. Luminal
WNT10B	Wnt family member 10B	12q13.12	7480	1.11	8.77	1.56E-17	7.25E-17	up Basal vs. Luminal
RFLNA	refilin A	12q24.31	144347	1.44	8.77	1.59E-17	7.40E-17	up Basal vs. Luminal
SLCO5A1	solute carrier organic anion transporter fam	8q13.3	81796	1.03	8.76	1.71E-17	7.89E-17	up Basal vs. Luminal
CDK6	cyclin dependent kinase 6	7q21.2	1021	1.02	8.73	2.15E-17	9.77E-17	up Basal vs. Luminal
IL32	interleukin 32	16p13.3	9235	1.06	8.72	2.44E-17	1.10E-16	up Basal vs. Luminal
HTR3A	5-hydroxytryptamine receptor 3A	11q23.2	3359	1.16	8.71	2.59E-17	1.16E-16	up Basal vs. Luminal
FABP7	fatty acid binding protein 7	6q22.31	2173	2.13	8.67	3.47E-17	1.52E-16	up Basal vs. Luminal
CD7	CD7 molecule	17q25.3	924	1.34	8.66	3.66E-17	1.60E-16	up Basal vs. Luminal
SOX8	SRY-box 8	16p13.3	30812	1.28	8.64	4.46E-17	1.91E-16	up Basal vs. Luminal
CRHR1	corticotropin releasing hormone receptor 1	17q21.31	1394	1.48	8.62	5.03E-17	2.14E-16	up Basal vs. Luminal
ADGRG6	adhesion G protein-coupled receptor G6	6q24.2	57211	1.53	8.62	5.04E-17	2.15E-16	up Basal vs. Luminal
SOX11	SRY-box 11	2p25.2	6664	1.39	8.62	5.26E-17	2.23E-16	up Basal vs. Luminal
DOK2	docking protein 2	8p21.3	9046	1.09	8.61	5.79E-17	2.43E-16	up Basal vs. Luminal
CCNE1	cyclin E1	19q12	898	1.01	8.60	5.96E-17	2.50E-16	up Basal vs. Luminal
COL4A4	collagen type IV alpha 4 chain	2q36.3	1286	1.18	8.59	6.55E-17	2.72E-16	up Basal vs. Luminal
IL2RA	interleukin 2 receptor subunit alpha	10p15.1	3559	1.34	8.57	7.71E-17	3.17E-16	up Basal vs. Luminal
ERICH5	glutamate rich 5	8q22.2	203111	1.34	8.55	8.98E-17	3.68E-16	up Basal vs. Luminal
FABP5	fatty acid binding protein 5	8q21.13	2171	1.18	8.55	9.07E-17	3.71E-16	up Basal vs. Luminal
ULBP1	UL16 binding protein 1	6q25.1	80329	1.28	8.54	9.70E-17	3.94E-16	up Basal vs. Luminal
GSTP1	glutathione S-transferase pi 1	11q13.2	2950	1.15	8.54	9.79E-17	3.97E-16	up Basal vs. Luminal
CALHM6	calcium homeostasis modulator family mer	6q22.1	441168	1.09	8.50	1.29E-16	5.19E-16	up Basal vs. Luminal
CSTA	cystatin A	3q21.1	1475	1.33	8.50	1.34E-16	5.38E-16	up Basal vs. Luminal
TGM1	transglutaminase 1	14q12	7051	1.06	8.50	1.36E-16	5.45E-16	up Basal vs. Luminal
CHAC1	ChaC glutathione specific gamma-glutamyl	15q15.1	79094	1.11	8.49	1.38E-16	5.50E-16	up Basal vs. Luminal
TNFSF12-TNFS	TNFSF12-TNFSF13 readthrough	17p13.1	407977	2.17	8.48	1.56E-16	6.19E-16	up Basal vs. Luminal
CXCL1	C-X-C motif chemokine ligand 1	4q13.3	2919	1.58	8.48	1.60E-16	6.34E-16	up Basal vs. Luminal
IVL	involucrin	1q21.3	3713	1.36	8.46	1.84E-16	7.25E-16	up Basal vs. Luminal
PCBP3	poly(rC) binding protein 3	21q22.3	54039	1.25	8.45	1.92E-16	7.59E-16	up Basal vs. Luminal
LCT	lactase	2q21.3	3938	1.07	8.44	2.15E-16	8.39E-16	up Basal vs. Luminal
CHI3L1	chitinase 3 like 1	1q32.1	1116	1.78	8.43	2.18E-16	8.51E-16	up Basal vs. Luminal
OPRK1	opioid receptor kappa 1	8q11.23	4986	1.14	8.40	2.75E-16	1.06E-15	up Basal vs. Luminal
SLC7A5	solute carrier family 7 member 5	16q24.2	8140	1.17	8.39	3.04E-16	1.16E-15	up Basal vs. Luminal
CXCL3	C-X-C motif chemokine ligand 3	4q13.3	2921	1.10	8.39	3.17E-16	1.21E-15	up Basal vs. Luminal
SLCO4A1	solute carrier organic anion transporter fam	20q13.33	28231	1.32	8.37	3.46E-16	1.31E-15	up Basal vs. Luminal
FUT3	fucosyltransferase 3 (Lewis blood group)	19p13.3	2525	1.92	8.37	3.64E-16	1.37E-15	up Basal vs. Luminal
ECEL1	endothelin converting enzyme like 1	2q37.1	9427	1.60	8.33	4.81E-16	1.78E-15	up Basal vs. Luminal
PRRX2	paired related homeobox 2	9q34.11	51450	1.00	8.32	5.09E-16	1.87E-15	up Basal vs. Luminal
STAC	SH3 and cysteine rich domain	3p22.3-p22.2	6769	1.23	8.29	6.39E-16	2.32E-15	up Basal vs. Luminal
DUSP9	dual specificity phosphatase 9	Xq28	1852	1.47	8.27	7.44E-16	2.67E-15	up Basal vs. Luminal
NPM2	nucleophosmin/nucleoplasmin 2	8p21.3	10361	1.17	8.27	7.56E-16	2.71E-15	up Basal vs. Luminal
PRF1	perforin 1	10q22.1	5551	1.22	8.27	7.89E-16	2.82E-15	up Basal vs. Luminal
ASS1	argininosuccinate synthase 1	9q34.11	445	1.33	8.25	9.10E-16	3.23E-15	up Basal vs. Luminal
ROPN1	rhophilin associated tail protein 1	3q21.1	54763	1.66	8.25	9.24E-16	3.27E-15	up Basal vs. Luminal
RAET1L	retinoic acid early transcript 1L	6q25.1	154064	1.02	8.23	1.08E-15	3.77E-15	up Basal vs. Luminal
HRCT1	histidine rich carboxyl terminus 1	9p13.3	646962	1.42	8.22	1.12E-15	3.89E-15	up Basal vs. Luminal
TNFRSF8	TNF receptor superfamily member 8	1p36.22	943	1.00	8.21	1.18E-15	4.09E-15	up Basal vs. Luminal
PHGDH	phosphoglycerate dehydrogenase	1p12	26227	1.17	8.21	1.25E-15	4.33E-15	up Basal vs. Luminal
SDCBP2	syndecan binding protein 2	20p13	27111	1.01	8.18	1.53E-15	5.24E-15	up Basal vs. Luminal
B3GNT3	UDP-GlcNAc:betaGal beta-1,3-N-acetylglu	19p13.11	10331	1.73	8.17	1.62E-15	5.51E-15	up Basal vs. Luminal
PBX4	PBX homeobox 4	19p13.11	80714	1.03	8.17	1.67E-15	5.67E-15	up Basal vs. Luminal
BMP2	bone morphogenetic protein 2	20p12.3	650	1.00	8.14	2.11E-15	7.07E-15	up Basal vs. Luminal
KYNU	kynureninase	2q22.2	8942	1.31	8.13	2.23E-15	7.46E-15	up Basal vs. Luminal
KRT83	keratin 83	12q13.13	3889	1.10	8.12	2.40E-15	7.96E-15	up Basal vs. Luminal
ACHE	acetylcholinesterase (Cartwright blood grou	7q22.1	43	1.07	8.12	2.42E-15	8.01E-15	up Basal vs. Luminal
SLC1A6	solute carrier family 1 member 6	19p13.12	6511	1.25	8.10	2.79E-15	9.15E-15	up Basal vs. Luminal
CLEC4E	C-type lectin domain family 4 member E	12p13.31	26253	1.33	8.07	3.45E-15	1.12E-14	up Basal vs. Luminal
SLC16A10	solute carrier family 16 member 10	6q21	117247	1.07	8.06	3.60E-15	1.16E-14	up Basal vs. Luminal
AIM2	absent in melanoma 2	1q23.1-q23.2	9447	1.28	8.06	3.65E-15	1.17E-14	up Basal vs. Luminal
FAM3D	family with sequence similarity 3 member D	3p14.2	131177	1.48	8.06	3.68E-15	1.18E-14	up Basal vs. Luminal
PTCHD1	patched domain containing 1	Xp22.11	139411	1.41	8.06	3.83E-15	1.22E-14	up Basal vs. Luminal
ABCA13	ATP binding cassette subfamily A member	7p12.3	154664	1.41	8.03	4.71E-15	1.48E-14	up Basal vs. Luminal
HLA-DOB	major histocompatibility complex, class II, D	6p21.32	3112	1.29	8.01	5.49E-15	1.70E-14	up Basal vs. Luminal
SMC1B	structural maintenance of chromosomes 1E	22q13.31	27127	1.45	8.00	5.98E-15	1.84E-14	up Basal vs. Luminal
CCL7	C-C motif chemokine ligand 7	17q12	6354	1.28	7.99	6.02E-15	1.85E-14	up Basal vs. Luminal
PDCD1	programmed cell death 1	2q37.3	5133	1.33	7.99	6.30E-15	1.93E-14	up Basal vs. Luminal
EGFL6	EGF like domain multiple 6	Xp22.2	25975	1.26	7.96	7.73E-15	2.33E-14	up Basal vs. Luminal
ZIC5	Zic family member 5	13q32.3	85416	1.29	7.93	9.55E-15	2.85E-14	up Basal vs. Luminal
LAMP3	lysosomal associated membrane protein 3	3q27.1	27074	1.30	7.92	1.02E-14	3.03E-14	up Basal vs. Luminal
L1CAM	L1 cell adhesion molecule	Xq28	3897	1.61	7.91	1.12E-14	3.29E-14	up Basal vs. Luminal
HSD17B2	hydroxysteroid 17-beta dehydrogenase 2	16q23.3	3294	1.81	7.88	1.41E-14	4.10E-14	up Basal vs. Luminal
PLA2G7	phospholipase A2 group VII	6p12.3	7941	1.21	7.87	1.45E-14	4.20E-14	up Basal vs. Luminal
ALDOC	aldolase, fructose-bisphosphate C	17q11.2	230	1.18	7.87	1.48E-14	4.27E-14	up Basal vs. Luminal
TPRXL	tetrapeptide repeat homeobox like	3p25.1	348825	1.26	7.86	1.57E-14	4.50E-14	up Basal vs. Luminal
PTX3	pentraxin 3	3q25.32	5806	1.42	7.85	1.78E-14	5.11E-14	up Basal vs. Luminal
ARL9	ADP ribosylation factor like GTPase 9	4q12	132946	1.09	7.83	2.05E-14	5.82E-14	up Basal vs. Luminal
HPDL	4-hydroxyphenylpyruvate dioxygenase like	1p34.1	84842	1.13	7.81	2.41E-14	6.71E-14	up Basal vs. Luminal
KRT16	keratin 16	17q21.2	3868	2.05	7.80	2.51E-14	6.99E-14	up Basal vs. Luminal
CTLA4	cytotoxic T-lymphocyte associated protein 4	2q33.2	1493	1.31	7.79	2.79E-14	7.70E-14	up Basal vs. Luminal
IFNG	interferon gamma	12q15	3458	1.11	7.75	3.62E-14	9.88E-14	up Basal vs. Luminal
ACY3	aminoacylase 3	11q13.2	91703	1.34	7.74	3.96E-14	1.07E-13	up Basal vs. Luminal
DNER	delta/notch like EGF repeat containing	2q36.3	92737	1.42	7.74	4.00E-14	1.08E-13	up Basal vs. Luminal
IL6	interleukin 6	7p15.3	3569	1.60	7.73	4.29E-14	1.15E-13	up Basal vs. Luminal
CX3CL1	C-X3-C motif chemokine ligand 1	16q21	6376	1.10	7.72	4.53E-14	1.21E-13	up Basal vs. Luminal
CASP14	caspase 14	19p13.12	23581	1.93	7.71	4.66E-14	1.24E-13	up Basal vs. Luminal
BARX1	BARX homeobox 1	9q22.32	56033	1.24	7.70	5.14E-14	1.36E-13	up Basal vs. Luminal
GLYATL2	glycine-N-acyltransferase like 2	11q12.1	219970	1.87	7.68	5.93E-14	1.55E-13	up Basal vs. Luminal
TRABD2A	TraB domain containing 2A	2p11.2	129293	1.08	7.67	6.31E-14	1.65E-13	up Basal vs. Luminal
DMRTA1	DMRT like family A1	9p21.3	63951	1.25	7.67	6.53E-14	1.70E-13	up Basal vs. Luminal
HK3	hexokinase 3	5q35.2	3101	1.21	7.66	7.08E-14	1.83E-13	up Basal vs. Luminal
NKX2-5	NK2 homeobox 5	5q35.1	1482	1.34	7.65	7.15E-14	1.85E-13	up Basal vs. Luminal
CCL5	C-C motif chemokine ligand 5	17q12	6352	1.19	7.65	7.41E-14	1.90E-13	up Basal vs. Luminal
CD8B	CD8b molecule	2p11.2	926	1.34	7.65	7.42E-14	1.90E-13	up Basal vs. Luminal
GABRE	gamma-aminobutyric acid type A receptor e	Xq28	2564	1.34	7.64	7.79E-14	1.99E-13	up Basal vs. Luminal
FSD1	fibronectin type III and SPRY domain conta	19p13.3	79187	1.01	7.64	7.79E-14	1.99E-13	up Basal vs. Luminal
IL12A	interleukin 12A	3q25.33	3592	1.09	7.63	8.46E-14	2.14E-13	up Basal vs. Luminal

Symbol	Description	Cytoband	Entrez Gene ID	Basal vs. Luminal (log2-ratio)	t	p-value[1]	q-value	Associated to
IL21R	interleukin 21 receptor	16p12.1	50615	1.14	7.63	8.57E-14	2.16E-13	up Basal vs. Luminal
ULBP3	UL16 binding protein 3	6q25.1	79465	1.03	7.62	8.86E-14	2.23E-13	up Basal vs. Luminal
NKG7	natural killer cell granule protein 7	19q13.41	4818	1.25	7.61	9.73E-14	2.43E-13	up Basal vs. Luminal
GPR55	G protein-coupled receptor 55	2q37.1	9290	1.05	7.60	1.02E-13	2.54E-13	up Basal vs. Luminal
CD38	CD38 molecule	4p15.32	952	1.47	7.60	1.06E-13	2.62E-13	up Basal vs. Luminal
CCL20	C-C motif chemokine ligand 20	2q36.3	6364	1.16	7.60	1.08E-13	2.67E-13	up Basal vs. Luminal
FOXD1	forkhead box D1	5q13.2	2297	1.48	7.59	1.12E-13	2.76E-13	up Basal vs. Luminal
KCNJ15	potassium voltage-gated channel subfamily	21q22.13-q22.2	3772	1.03	7.59	1.14E-13	2.82E-13	up Basal vs. Luminal
RHOV	ras homolog family member V	15q15.1	171177	1.05	7.58	1.22E-13	2.99E-13	up Basal vs. Luminal
PAX7	paired box 7	1p36.13	5081	1.72	7.58	1.23E-13	3.02E-13	up Basal vs. Luminal
MT1M	metallothionein 1M	16q13	4499	1.23	7.55	1.50E-13	3.62E-13	up Basal vs. Luminal
DKK1	dickkopf WNT signaling pathway inhibitor 1	10q21.1	22943	1.66	7.55	1.52E-13	3.66E-13	up Basal vs. Luminal
CD70	CD70 molecule	19p13.3	970	1.02	7.52	1.91E-13	4.52E-13	up Basal vs. Luminal
CHRM1	cholinergic receptor muscarinic 1	11q12.3	1128	1.56	7.51	1.97E-13	4.64E-13	up Basal vs. Luminal
PATL2	PAT1 homolog 2	15q21.1	197135	1.02	7.49	2.22E-13	5.17E-13	up Basal vs. Luminal
FUT7	fucosyltransferase 7	9q34.3	2529	1.01	7.49	2.25E-13	5.26E-13	up Basal vs. Luminal
RGS2	regulator of G protein signaling 2	1q31.2	5997	1.02	7.48	2.44E-13	5.65E-13	up Basal vs. Luminal
CRYAB	crystallin alpha B	11q23.1	1410	1.24	7.48	2.48E-13	5.73E-13	up Basal vs. Luminal
COL26A1	collagen type XXVI alpha 1 chain	7q22.1	136227	1.03	7.47	2.57E-13	5.92E-13	up Basal vs. Luminal
LIME1	Lck interacting transmembrane adaptor 1	20q13.33	54923	1.09	7.47	2.71E-13	6.21E-13	up Basal vs. Luminal
APOBEC3A	apolipoprotein B mRNA editing enzyme cat	22q13.1	200315	1.16	7.46	2.76E-13	6.32E-13	up Basal vs. Luminal
PRKCQ	protein kinase C theta	10p15.1	5588	1.20	7.43	3.46E-13	7.77E-13	up Basal vs. Luminal
RASGRF1	Ras protein specific guanine nucleotide rele	15q25.1	5923	1.13	7.41	4.06E-13	9.05E-13	up Basal vs. Luminal
ICAM5	intercellular adhesion molecule 5	19p13.2	7087	1.17	7.38	4.90E-13	1.08E-12	up Basal vs. Luminal
CCL8	C-C motif chemokine ligand 8	17q12	6355	1.22	7.38	4.91E-13	1.08E-12	up Basal vs. Luminal
CLIC3	chloride intracellular channel 3	9q34.3	9022	1.23	7.36	5.45E-13	1.19E-12	up Basal vs. Luminal
KLRD1	killer cell lectin like receptor D1	12p13.2	3824	1.05	7.34	6.41E-13	1.38E-12	up Basal vs. Luminal
AKR1B15	aldo-keto reductase family 1 member B15	7q33	441282	1.17	7.34	6.69E-13	1.43E-12	up Basal vs. Luminal
SLC15A2	solute carrier family 15 member 2	3q13.33	6565	1.18	7.33	6.74E-13	1.44E-12	up Basal vs. Luminal
MCTP2	multiple C2 and transmembrane domain co	15q26.2	55784	1.14	7.33	6.89E-13	1.47E-12	up Basal vs. Luminal
TMEM71	transmembrane protein 71	8q24.22	137835	1.00	7.33	7.16E-13	1.52E-12	up Basal vs. Luminal
CST7	cystatin F	20p11.21	8530	1.11	7.32	7.28E-13	1.55E-12	up Basal vs. Luminal
FGFR4	fibroblast growth factor receptor 4	5q35.2	2264	1.46	7.32	7.47E-13	1.59E-12	up Basal vs. Luminal
GJB3	gap junction protein beta 3	1p34.3	2707	1.40	7.32	7.65E-13	1.62E-12	up Basal vs. Luminal
SPIB	Spi-B transcription factor	19q13.33	6689	1.47	7.30	8.58E-13	1.80E-12	up Basal vs. Luminal
GNLY	granulysin	2p11.2	10578	1.24	7.30	8.78E-13	1.84E-12	up Basal vs. Luminal
IL18RAP	interleukin 18 receptor accessory protein	2q12.1	8807	1.02	7.29	9.22E-13	1.93E-12	up Basal vs. Luminal
GDF5	growth differentiation factor 5	20q11.22	8200	1.19	7.28	9.87E-13	2.05E-12	up Basal vs. Luminal
WNT10A	Wnt family member 10A	2q35	80326	1.07	7.28	9.97E-13	2.07E-12	up Basal vs. Luminal
MATK	megakaryocyte-associated tyrosine kinase	19p13.3	4145	1.20	7.28	9.99E-13	2.08E-12	up Basal vs. Luminal
ARNTL2	aryl hydrocarbon receptor nuclear transloca	12p11.23	56938	1.05	7.26	1.09E-12	2.26E-12	up Basal vs. Luminal
LCK	LCK proto-oncogene, Src family tyrosine ki	1p35.2	3932	1.24	7.24	1.31E-12	2.67E-12	up Basal vs. Luminal
LTA	lymphotoxin alpha	6p21.33	4049	1.11	7.23	1.42E-12	2.87E-12	up Basal vs. Luminal
PKP1	plakophilin 1	1q32.1	5317	1.79	7.21	1.55E-12	3.12E-12	up Basal vs. Luminal
TRPV6	transient receptor potential cation channel s	7q34	55503	1.69	7.18	1.87E-12	3.72E-12	up Basal vs. Luminal
CDHR1	cadherin related family member 1	10q23.1	92211	1.16	7.17	2.03E-12	4.02E-12	up Basal vs. Luminal
S1PR4	sphingosine-1-phosphate receptor 4	19p13.3	8698	1.10	7.17	2.12E-12	4.20E-12	up Basal vs. Luminal
NIPAL4	NIPA like domain containing 4	5q33.3	348938	1.02	7.16	2.25E-12	4.44E-12	up Basal vs. Luminal
ACAP1	ArfGAP with coiled-coil, ankyrin repeat and	17p13.1	9744	1.20	7.12	2.81E-12	5.45E-12	up Basal vs. Luminal
NCR3	natural cytotoxicity triggering receptor 3	6p21.33	259197	1.05	7.11	3.06E-12	5.88E-12	up Basal vs. Luminal
MAL	mal, T cell differentiation protein	2q11.1	4118	1.16	7.11	3.09E-12	5.94E-12	up Basal vs. Luminal
CXCR6	C-X-C motif chemokine receptor 6	3p21.31	10663	1.08	7.10	3.30E-12	6.30E-12	up Basal vs. Luminal
APCDD1L	APC down-regulated 1 like	20q13.32	164284	1.14	7.10	3.37E-12	6.42E-12	up Basal vs. Luminal
NCCRP1	non-specific cytotoxic cell receptor protein 1	19q13.2	342897	1.64	7.09	3.51E-12	6.67E-12	up Basal vs. Luminal
OXGR1	oxoglutarate receptor 1	13q32.1	27199	1.24	7.09	3.59E-12	6.82E-12	up Basal vs. Luminal
FGFBP1	fibroblast growth factor binding protein 1	4p15.32	9982	1.45	7.08	3.90E-12	7.35E-12	up Basal vs. Luminal
KRT6C	keratin 6C	12q13.13	286887	1.55	7.05	4.57E-12	8.49E-12	up Basal vs. Luminal
SPOCK2	SPARC (osteonectin), cwcv and kazal like d	10q22.1	9806	1.01	7.04	4.91E-12	9.10E-12	up Basal vs. Luminal
NRG2	neuregulin 2	5q31.2	9542	1.23	7.03	5.20E-12	9.55E-12	up Basal vs. Luminal
SLURP1	secreted LY6/PLAUR domain containing 1	8q24.3	57152	1.07	7.00	6.31E-12	1.14E-11	up Basal vs. Luminal
ALKAL2	ALK and LTK ligand 2	2p25.3	285016	1.08	7.00	6.64E-12	1.20E-11	up Basal vs. Luminal
GBP5	guanylate binding protein 5	1p22.2	115362	1.24	7.00	6.66E-12	1.20E-11	up Basal vs. Luminal
ANGPTL4	angiopoietin like 4	19p13.2	51129	1.27	6.99	6.81E-12	1.23E-11	up Basal vs. Luminal
PSTPIP1	proline-serine-threonine phosphatase intera	15q24.3	9051	1.06	6.97	8.05E-12	1.43E-11	up Basal vs. Luminal
CD79B	CD79b molecule	17q23.3	974	1.17	6.96	8.20E-12	1.46E-11	up Basal vs. Luminal
CR1	complement C3b/C4b receptor 1 (Knops bl	1q32.2	1378	1.28	6.95	8.95E-12	1.58E-11	up Basal vs. Luminal
ZBED2	zinc finger BED-type containing 2	3q13.13	79413	1.20	6.95	9.00E-12	1.59E-11	up Basal vs. Luminal
CD6	CD6 molecule	11q12.2	923	1.08	6.94	9.70E-12	1.70E-11	up Basal vs. Luminal
SLC22A3	solute carrier family 22 member 3	6q25.3	6581	1.19	6.94	9.86E-12	1.73E-11	up Basal vs. Luminal
SYCE1L	synaptonemal complex central element pro	16q23.1	100130958	1.14	6.93	1.02E-11	1.78E-11	up Basal vs. Luminal
ADTRP	androgen dependent TFPI regulating prote	6p24.1	84830	1.11	6.93	1.04E-11	1.82E-11	up Basal vs. Luminal
ROPN1B	rhophilin associated tail protein 1B	3q21.2	152015	1.38	6.93	1.05E-11	1.83E-11	up Basal vs. Luminal
PTPRCAP	protein tyrosine phosphatase receptor type	11q13.2	5790	1.16	6.92	1.08E-11	1.87E-11	up Basal vs. Luminal
TBX21	T-box 21	17q21.32	30009	1.09	6.92	1.09E-11	1.89E-11	up Basal vs. Luminal
IRX6	iroquois homeobox 6	16q12.2	79190	1.12	6.91	1.15E-11	1.98E-11	up Basal vs. Luminal
IL2RG	interleukin 2 receptor subunit gamma	Xq13.1	3561	1.06	6.91	1.17E-11	2.03E-11	up Basal vs. Luminal
PLCH1	phospholipase C eta 1	3q25.31	23007	1.66	6.91	1.20E-11	2.06E-11	up Basal vs. Luminal
CDH3	cadherin 3	16q22.1	1001	1.22	6.85	1.68E-11	2.84E-11	up Basal vs. Luminal
IL34	interleukin 34	16q22.1	146433	1.01	6.83	2.01E-11	3.37E-11	up Basal vs. Luminal
GZMM	granzyme M	19p13.3	3004	1.23	6.81	2.20E-11	3.65E-11	up Basal vs. Luminal
XCL2	X-C motif chemokine ligand 2	1q24.2	6846	1.02	6.80	2.46E-11	4.05E-11	up Basal vs. Luminal
COLCA1	colorectal cancer associated 1	11q23.1	399948	1.21	6.80	2.47E-11	4.07E-11	up Basal vs. Luminal
COL4A3	collagen type IV alpha 3 chain	2q36.3	1285	1.03	6.79	2.58E-11	4.23E-11	up Basal vs. Luminal
PCP4L1	Purkinje cell protein 4 like 1	1q23.3	654790	1.19	6.78	2.68E-11	4.39E-11	up Basal vs. Luminal
SLC4A11	solute carrier family 4 member 11	20p13	83959	1.04	6.78	2.74E-11	4.48E-11	up Basal vs. Luminal
LY6D	lymphocyte antigen 6 family member D	8q24.3	8581	1.49	6.77	3.00E-11	4.88E-11	up Basal vs. Luminal
LIPG	lipase G, endothelial type	18q21.1	9388	1.06	6.73	3.65E-11	5.82E-11	up Basal vs. Luminal
CD3D	CD3d molecule	11q23.3	915	1.24	6.73	3.66E-11	5.83E-11	up Basal vs. Luminal
ICOS	inducible T cell costimulator	2q33.2	29851	1.19	6.73	3.82E-11	6.06E-11	up Basal vs. Luminal
TOX	thymocyte selection associated high mobilit	8q12.1	9760	1.06	6.69	4.88E-11	7.64E-11	up Basal vs. Luminal
FCHO1	FCH domain only 1	19p13.11	23149	1.03	6.69	4.98E-11	7.77E-11	up Basal vs. Luminal
LYZ	lysozyme	12q15	4069	1.04	6.69	5.01E-11	7.81E-11	up Basal vs. Luminal
LHX2	LIM homeobox 2	9q33.3	9355	1.05	6.68	5.23E-11	8.10E-11	up Basal vs. Luminal
KRT6A	keratin 6A	12q13.13	3853	1.89	6.67	5.53E-11	8.53E-11	up Basal vs. Luminal
VSTM2L	V-set and transmembrane domain containi	20q11.23	128434	1.44	6.65	6.44E-11	9.81E-11	up Basal vs. Luminal
LY6K	lymphocyte antigen 6 family member K	8q24.3	54742	1.45	6.63	7.19E-11	1.09E-10	up Basal vs. Luminal
CXCL10	C-X-C motif chemokine ligand 10	4q21.1	3627	1.13	6.62	7.51E-11	1.14E-10	up Basal vs. Luminal
SLAMF1	signaling lymphocytic activation molecule fa	1q23.3	6504	1.14	6.62	7.72E-11	1.17E-10	up Basal vs. Luminal
SCEL	sciellin	13q22.3	8796	1.02	6.62	7.76E-11	1.17E-10	up Basal vs. Luminal
MMP9	matrix metallopeptidase 9	20q13.12	4318	1.26	6.60	8.37E-11	1.26E-10	up Basal vs. Luminal
AQP9	aquaporin 9	15q21.3	366	1.41	6.60	8.87E-11	1.32E-10	up Basal vs. Luminal
C5orf46	chromosome 5 open reading frame 46	5q32	389336	1.14	6.59	8.89E-11	1.33E-10	up Basal vs. Luminal
FBN3	fibrillin 3	19p13.2	84467	1.13	6.57	1.04E-10	1.54E-10	up Basal vs. Luminal
PSCA	prostate stem cell antigen	8q24.3	8000	1.72	6.55	1.18E-10	1.72E-10	up Basal vs. Luminal
CHRDL2	chordin like 2	11q13.4	25884	1.23	6.54	1.22E-10	1.78E-10	up Basal vs. Luminal
DEFB1	defensin beta 1	8p23.1	1672	1.38	6.53	1.30E-10	1.89E-10	up Basal vs. Luminal
BMP7	bone morphogenetic protein 7	20q13.31	655	1.33	6.53	1.31E-10	1.91E-10	up Basal vs. Luminal
ALPK2	alpha kinase 2	18q21.31-q21.32	115701	1.00	6.50	1.59E-10	2.28E-10	up Basal vs. Luminal
ATP10B	ATPase phospholipid transporting 10B (put	5q34	23120	1.02	6.50	1.62E-10	2.33E-10	up Basal vs. Luminal
TRPM6	transient receptor potential cation channel s	9q21.13	140803	1.02	6.49	1.70E-10	2.43E-10	up Basal vs. Luminal
TDO2	tryptophan 2,3-dioxygenase	4q32.1	6999	1.08	6.48	1.78E-10	2.54E-10	up Basal vs. Luminal
GPR12	G protein-coupled receptor 12	13q12.13	2835	1.11	6.47	1.92E-10	2.73E-10	up Basal vs. Luminal
KRT81	keratin 81	12q13.13	3887	1.65	6.46	2.06E-10	2.91E-10	up Basal vs. Luminal
ADGRF1	adhesion G protein-coupled receptor F1	6p12.3|6	266977	1.33	6.46	2.10E-10	2.96E-10	up Basal vs. Luminal
CALB2	calbindin 2	16q22.2	794	1.22	6.46	2.12E-10	2.98E-10	up Basal vs. Luminal
EMILIN3	elastin microfibril interfacer 3	20q12	90187	1.15	6.45	2.13E-10	3.00E-10	up Basal vs. Luminal
CA9	carbonic anhydrase 9	9p13.3	768	1.70	6.44	2.39E-10	3.33E-10	up Basal vs. Luminal
CD247	CD247 molecule	1q24.2	919	1.04	6.43	2.42E-10	3.37E-10	up Basal vs. Luminal

Symbol	Description	Cytoband	Entrez Gene ID	Basal vs. Luminal (log2-ratio)	t	p-value[1]	q-value	Associated to
TTYH1	tweety family member 1	19q13.42	57348	1.22	6.42	2.57E-10	3.57E-10	up Basal vs. Luminal
GJB5	gap junction protein beta 5	1p34.3	2709	1.23	6.42	2.60E-10	3.60E-10	up Basal vs. Luminal
ZIC1	Zic family member 1	3q24	7545	1.45	6.40	2.98E-10	4.08E-10	up Basal vs. Luminal
SLPI	secretory leukocyte peptidase inhibitor	20q13.12	6590	1.48	6.40	2.99E-10	4.10E-10	up Basal vs. Luminal
POU2AF1	POU class 2 homeobox associating factor 1	11q23.1	5450	1.38	6.40	3.00E-10	4.11E-10	up Basal vs. Luminal
CD27	CD27 molecule	12p13.31	939	1.13	6.40	3.08E-10	4.20E-10	up Basal vs. Luminal
C6orf15	chromosome 6 open reading frame 15	6p21.33	29113	1.04	6.39	3.13E-10	4.27E-10	up Basal vs. Luminal
SIRPG	signal regulatory protein gamma	20p13	55423	1.15	6.36	3.82E-10	5.13E-10	up Basal vs. Luminal
RIPK4	receptor interacting serine/threonine kinase	21q22.3	54101	1.03	6.36	3.90E-10	5.23E-10	up Basal vs. Luminal
MSL3P1	MSL complex subunit 3 pseudogene 1	2q37.1	151507	1.06	6.35	4.14E-10	5.53E-10	up Basal vs. Luminal
VSNL1	visinin like 1	2p24.2	7447	1.02	6.31	5.05E-10	6.65E-10	up Basal vs. Luminal
ELF5	E74 like ETS transcription factor 5	11p13	2001	1.78	6.30	5.41E-10	7.08E-10	up Basal vs. Luminal
KLF5	Kruppel like factor 5	13q22.1	688	1.01	6.29	5.86E-10	7.62E-10	up Basal vs. Luminal
KIF19	kinesin family member 19	17q25.1	124602	1.05	6.29	5.92E-10	7.69E-10	up Basal vs. Luminal
GZMA	granzyme A	5q11.2	3001	1.08	6.28	6.21E-10	8.06E-10	up Basal vs. Luminal
IRF4	interferon regulatory factor 4	6p25.3	3662	1.16	6.26	7.23E-10	9.30E-10	up Basal vs. Luminal
SLC6A14	solute carrier family 6 member 14	Xq23	11254	1.63	6.25	7.33E-10	9.41E-10	up Basal vs. Luminal
VPREB3	V-set pre-B cell surrogate light chain 3	22q11.23|22q11	29802	1.04	6.25	7.41E-10	9.50E-10	up Basal vs. Luminal
SIT1	signaling threshold regulating transmembra	9p13.3	27240	1.08	6.24	7.87E-10	1.01E-09	up Basal vs. Luminal
SPINK5	serine peptidase inhibitor, Kazal type 5	5q32	11005	1.18	6.23	8.24E-10	1.05E-09	up Basal vs. Luminal
KLHDC7B	kelch domain containing 7B	22q13.33	113730	1.11	6.22	9.20E-10	1.16E-09	up Basal vs. Luminal
CXCL11	C-X-C motif chemokine ligand 11	4q21.1	6373	1.17	6.19	1.07E-09	1.34E-09	up Basal vs. Luminal
LGALS2	galectin 2	22q13.1	3957	1.19	6.17	1.17E-09	1.45E-09	up Basal vs. Luminal
BBOX1	gamma-butyrobetaine hydroxylase 1	11p14.2	8424	1.58	6.16	1.31E-09	1.61E-09	up Basal vs. Luminal
ZAP70	zeta chain of T cell receptor associated pro	2q11.2	7535	1.11	6.15	1.33E-09	1.63E-09	up Basal vs. Luminal
SLAMF6	SLAM family member 6	1q23.2-q23.3	114836	1.09	6.15	1.33E-09	1.63E-09	up Basal vs. Luminal
FCN1	ficolin 1	9q34.3	2219	1.19	6.15	1.37E-09	1.67E-09	up Basal vs. Luminal
CXCR3	C-X-C motif chemokine receptor 3	Xq13.1	2833	1.04	6.14	1.47E-09	1.79E-09	up Basal vs. Luminal
ADAMDEC1	ADAM like decysin 1	8p21.2	27299	1.31	6.13	1.54E-09	1.87E-09	up Basal vs. Luminal
UGT8	UDP glycosyltransferase 8	4q26	7368	1.13	6.10	1.88E-09	2.24E-09	up Basal vs. Luminal
JSRP1	junctional sarcoplasmic reticulum protein 1	19p13.3	126306	1.13	6.09	1.90E-09	2.26E-09	up Basal vs. Luminal
TMEM40	transmembrane protein 40	3p25.2	55287	1.38	6.08	2.02E-09	2.39E-09	up Basal vs. Luminal
GPR171	G protein-coupled receptor 171	3q25.1	29909	1.02	6.08	2.09E-09	2.47E-09	up Basal vs. Luminal
CALML5	calmodulin like 5	10p15.1	51806	1.96	6.06	2.29E-09	2.68E-09	up Basal vs. Luminal
TIGIT	T cell immunoreceptor with Ig and ITIM dor	3q13.31	201633	1.13	6.05	2.51E-09	2.93E-09	up Basal vs. Luminal
PYHIN1	pyrin and HIN domain family member 1	1q23.1	149628	1.07	6.02	2.87E-09	3.31E-09	up Basal vs. Luminal
CYP39A1	cytochrome P450 family 39 subfamily A me	6p12.3	51302	1.06	6.01	3.04E-09	3.51E-09	up Basal vs. Luminal
UBASH3A	ubiquitin associated and SH3 domain conta	21q22.3	53347	1.05	5.99	3.42E-09	3.91E-09	up Basal vs. Luminal
GSTA1	glutathione S-transferase alpha 1	6p12.2	2938	1.30	5.99	3.49E-09	3.97E-09	up Basal vs. Luminal
CD3E	CD3e molecule	11q23.3	916	1.10	5.96	4.05E-09	4.55E-09	up Basal vs. Luminal
COL22A1	collagen type XXII alpha 1 chain	8q24.23-q24.3	169044	1.03	5.96	4.14E-09	4.64E-09	up Basal vs. Luminal
SMOC1	SPARC related modular calcium binding 1	14q24.2	64093	1.27	5.96	4.24E-09	4.75E-09	up Basal vs. Luminal
FDCSP	follicular dendritic cell secreted protein	4q13.3|4q13	260436	1.87	5.94	4.74E-09	5.25E-09	up Basal vs. Luminal
FERMT1	fermitin family member 1	20p12.3	55612	1.18	5.93	4.85E-09	5.37E-09	up Basal vs. Luminal
PLA2G2D	phospholipase A2 group IID	1p36.12	26279	1.39	5.92	5.24E-09	5.77E-09	up Basal vs. Luminal
ATP13A5	ATPase 13A5	3q29	344905	1.40	5.90	5.97E-09	6.50E-09	up Basal vs. Luminal
S100B	S100 calcium binding protein B	21q22.3	6285	1.21	5.90	5.98E-09	6.51E-09	up Basal vs. Luminal
FCRL5	Fc receptor like 5	1q23.1	83416	1.33	5.87	7.08E-09	7.61E-09	up Basal vs. Luminal
PLA2G2A	phospholipase A2 group IIA	1p36.13	5320	1.63	5.83	8.57E-09	9.09E-09	up Basal vs. Luminal
CXCL2	C-X-C motif chemokine ligand 2	4q13.3	2920	1.17	5.83	8.79E-09	9.30E-09	up Basal vs. Luminal
BCL11B	BAF chromatin remodeling complex subuni	14q32.2	64919	1.00	5.82	9.35E-09	9.85E-09	up Basal vs. Luminal
S100A7	S100 calcium binding protein A7	1q21.3	6278	2.18	5.80	1.04E-08	1.09E-08	up Basal vs. Luminal
HR	HR lysine demethylase and nuclear recepto	8p21.3	55806	1.02	5.80	1.07E-08	1.12E-08	up Basal vs. Luminal
ZBP1	Z-DNA binding protein 1	20q13.31	81030	1.00	5.79	1.12E-08	1.16E-08	up Basal vs. Luminal
CD19	CD19 molecule	16p11.2	930	1.29	5.75	1.35E-08	1.38E-08	up Basal vs. Luminal
LTB	lymphotoxin beta	6p21.33	4050	1.18	5.75	1.36E-08	1.39E-08	up Basal vs. Luminal
CSAG3	CSAG family member 3	Xq28	389903	1.07	5.74	1.44E-08	1.46E-08	up Basal vs. Luminal
LY9	lymphocyte antigen 9	1q23.3	4063	1.03	5.72	1.66E-08	1.66E-08	up Basal vs. Luminal
PTGDS	prostaglandin D2 synthase	9q34.3	5730	1.15	5.71	1.75E-08	1.75E-08	up Basal vs. Luminal
S100A7A	S100 calcium binding protein A7A	1q21.3	338324	1.41	5.69	1.96E-08	1.94E-08	up Basal vs. Luminal
DPYSL5	dihydropyrimidinase like 5	2p23.3	56896	1.05	5.68	2.04E-08	2.01E-08	up Basal vs. Luminal
MMP1	matrix metallopeptidase 1	11q22.2	4312	1.48	5.66	2.25E-08	2.20E-08	up Basal vs. Luminal
KIF1A	kinesin family member 1A	2q37.3	547	1.54	5.61	2.94E-08	2.83E-08	up Basal vs. Luminal
CXCL8	C-X-C motif chemokine ligand 8	4q13.3	3576	1.03	5.61	3.08E-08	2.95E-08	up Basal vs. Luminal
PLAAT1	phospholipase A and acyltransferase 1	3q29	57110	1.10	5.60	3.16E-08	3.02E-08	up Basal vs. Luminal
ACTL8	actin like 8	1p36.13	81569	1.21	5.58	3.58E-08	3.39E-08	up Basal vs. Luminal
TRIM29	tripartite motif containing 29	11q23.3	23650	1.46	5.58	3.64E-08	3.44E-08	up Basal vs. Luminal
HOXB13	homeobox B13	17q21.32	10481	1.84	5.55	4.10E-08	3.84E-08	up Basal vs. Luminal
FOLR1	folate receptor 1	11q13.4	2348	1.49	5.54	4.39E-08	4.09E-08	up Basal vs. Luminal
LBP	lipopolysaccharide binding protein	20q11.23	3929	1.44	5.54	4.45E-08	4.14E-08	up Basal vs. Luminal
EDN2	endothelin 2	1p34.2	1907	1.03	5.54	4.45E-08	4.15E-08	up Basal vs. Luminal
PROM1	prominin 1	4p15.32	8842	1.65	5.53	4.57E-08	4.24E-08	up Basal vs. Luminal
PRAME	preferentially expressed antigen in melanor	22q11.22	23532	2.03	5.48	6.00E-08	5.48E-08	up Basal vs. Luminal
COL9A3	collagen type IX alpha 3 chain	20q13.33	1299	1.07	5.47	6.36E-08	5.79E-08	up Basal vs. Luminal
TCL1A	T cell leukemia/lymphoma 1A	14q32.13	8115	1.18	5.47	6.41E-08	5.83E-08	up Basal vs. Luminal
SH2D1A	SH2 domain containing 1A	Xq25	4068	1.01	5.46	6.97E-08	6.30E-08	up Basal vs. Luminal
FCRLA	Fc receptor like A	1q23.3	84824	1.04	5.41	8.98E-08	7.98E-08	up Basal vs. Luminal
SNORC	secondary ossification center associated re	2q37.1	389084	1.05	5.38	1.03E-07	9.10E-08	up Basal vs. Luminal
MMP7	matrix metallopeptidase 7	11q22.2	4316	1.16	5.36	1.14E-07	1.00E-07	up Basal vs. Luminal
LCN2	lipocalin 2	9q34.11	3934	1.42	5.33	1.33E-07	1.15E-07	up Basal vs. Luminal
ADAM6	ADAM metallopeptidase domain 6 (pseudo	14q32.33	8755	1.24	5.31	1.53E-07	1.31E-07	up Basal vs. Luminal
KRT4	keratin 4	12q13.13	3851	1.15	5.27	1.91E-07	1.60E-07	up Basal vs. Luminal
PCSK1N	proprotein convertase subtilisin/kexin type 1	Xp11.23	27344	1.24	5.26	2.01E-07	1.68E-07	up Basal vs. Luminal
TNFRSF17	TNF receptor superfamily member 17	16p13.13	608	1.07	5.23	2.31E-07	1.91E-07	up Basal vs. Luminal
ATP6V0A4	ATPase H+ transporting V0 subunit a4	7q34	50617	1.38	5.21	2.57E-07	2.11E-07	up Basal vs. Luminal
MMP12	matrix metallopeptidase 12	11q22.2	4321	1.25	5.20	2.73E-07	2.23E-07	up Basal vs. Luminal
WT1-AS	WT1 antisense RNA	11p13	51352	1.07	5.19	2.82E-07	2.30E-07	up Basal vs. Luminal
CD79A	CD79a molecule	19q13.2	973	1.22	5.15	3.39E-07	2.73E-07	up Basal vs. Luminal
KREMEN2	kringle containing transmembrane protein 2	16p13.3	79412	1.02	5.15	3.46E-07	2.78E-07	up Basal vs. Luminal
PCOLCE2	procollagen C-endopeptidase enhancer 2	3q23	26577	1.23	5.14	3.71E-07	2.97E-07	up Basal vs. Luminal
AOC1	amine oxidase copper containing 1	7q36.1	26	1.04	5.13	3.77E-07	3.02E-07	up Basal vs. Luminal
UBD	ubiquitin D	6p22.1	10537	1.13	5.12	4.05E-07	3.23E-07	up Basal vs. Luminal
FCER2	Fc fragment of IgE receptor II	19p13.2	2208	1.01	5.10	4.42E-07	3.50E-07	up Basal vs. Luminal
MAPK4	mitogen-activated protein kinase 4	18q21.1-q21.2	5596	1.11	5.08	4.84E-07	3.80E-07	up Basal vs. Luminal
MAGEA6	MAGE family member A6	Xq28	4105	1.24	5.06	5.46E-07	4.24E-07	up Basal vs. Luminal
FAM30A	family with sequence similarity 30 member	14q32.33	9834	1.04	5.02	6.61E-07	5.06E-07	up Basal vs. Luminal
MIA	MIA SH3 domain containing	19q13.2	8190	1.26	5.01	7.10E-07	5.41E-07	up Basal vs. Luminal
FCRL3	Fc receptor like 3	1q23.1	115352	1.01	4.98	8.37E-07	6.29E-07	up Basal vs. Luminal
CXCL9	C-X-C motif chemokine ligand 9	4q21.1	4283	1.10	4.97	8.74E-07	6.55E-07	up Basal vs. Luminal
KLK6	kallikrein related peptidase 6	19q13.41	5653	1.24	4.88	1.33E-06	9.70E-07	up Basal vs. Luminal
CAPN6	calpain 6	Xq23	827	1.21	4.86	1.47E-06	1.07E-06	up Basal vs. Luminal
DNAH11	dynein axonemal heavy chain 11	7p15.3	8701	1.00	4.77	2.27E-06	1.59E-06	up Basal vs. Luminal
MT1G	metallothionein 1G	16q13	4495	1.07	4.77	2.32E-06	1.63E-06	up Basal vs. Luminal
CLCA2	chloride channel accessory 2	1p22.3	9635	1.43	4.77	2.32E-06	1.63E-06	up Basal vs. Luminal
GZMK	granzyme K	5q11.2	3003	1.01	4.74	2.57E-06	1.79E-06	up Basal vs. Luminal
KLK8	kallikrein related peptidase 8	19q13.41	11202	1.06	4.71	3.00E-06	2.07E-06	up Basal vs. Luminal
ONECUT2	one cut homeobox 2	18q21.31	9480	1.12	4.69	3.41E-06	2.34E-06	up Basal vs. Luminal
FAM83A	family with sequence similarity 83 member	8q24.13	84985	1.06	4.67	3.59E-06	2.45E-06	up Basal vs. Luminal
SERPINB5	serpin family B member 5	18q21.33	5268	1.23	4.65	4.02E-06	2.72E-06	up Basal vs. Luminal
DCD	dermcidin	12q13.2	117159	1.46	4.64	4.20E-06	2.83E-06	up Basal vs. Luminal
CXCL13	C-X-C motif chemokine ligand 13	4q21.1	10563	1.37	4.53	7.13E-06	4.63E-06	up Basal vs. Luminal
CR2	complement C3d receptor 2	1q32.2	1380	1.18	4.48	8.80E-06	5.61E-06	up Basal vs. Luminal
CRYM	crystallin mu	16p12.2	1428	1.07	4.47	9.15E-06	5.82E-06	up Basal vs. Luminal
KCNK1	potassium two pore domain channel subfar	1q42.2	3775	1.02	4.43	1.11E-05	6.94E-06	up Basal vs. Luminal
ZIC2	Zic family member 2	13q32.3	7546	1.14	4.36	1.51E-05	9.24E-06	up Basal vs. Luminal
SAA1	serum amyloid A1	11p15.1	6288	1.14	4.35	1.56E-05	9.57E-06	up Basal vs. Luminal
WT1	WT1 transcription factor	11p13	7490	1.15	4.28	2.17E-05	1.30E-05	up Basal vs. Luminal
GABRP	gamma-aminobutyric acid type A receptor p	5q35.1	2568	1.33	4.27	2.26E-05	1.35E-05	up Basal vs. Luminal

Symbol	Description	Cytoband	Entrez Gene ID	Basal vs. Luminal (log2-ratio)	t	p-value[1]	q-value	Associated to
AQP5	aquaporin 5	12q13.12	362	1.15	4.26	2.31E-05	1.38E-05	up Basal vs. Luminal
HOXC11	homeobox C11	12q13.13	3227	1.21	4.24	2.59E-05	1.53E-05	up Basal vs. Luminal
PPP1R1A	protein phosphatase 1 regulatory inhibitor s	12q13.2	5502	1.06	4.23	2.71E-05	1.59E-05	up Basal vs. Luminal
MUC16	mucin 16, cell surface associated	19p13.2	94025	1.30	4.20	3.09E-05	1.79E-05	up Basal vs. Luminal
CP	ceruloplasmin	3q24-q25.1	1356	1.31	4.14	3.90E-05	2.23E-05	up Basal vs. Luminal
BRINP3	BMP/retinoic acid inducible neural specific 3	1q31.1	339479	1.28	4.13	4.19E-05	2.37E-05	up Basal vs. Luminal
MUC15	mucin 15, cell surface associated	11p14.2	143662	1.15	4.00	7.14E-05	3.87E-05	up Basal vs. Luminal
KRT6B	keratin 6B	12q13.13	3854	1.28	3.97	7.85E-05	4.21E-05	up Basal vs. Luminal
HOTAIR	HOX transcript antisense RNA	12q13.13	100124700	1.15	3.95	8.70E-05	4.63E-05	up Basal vs. Luminal
WIF1	WNT inhibitory factor 1	12q14.3	11197	1.09	3.91	1.02E-04	5.36E-05	up Basal vs. Luminal
MAB21L4	mab-21 like 4	2q37.3	79919	1.06	3.86	1.27E-04	6.56E-05	up Basal vs. Luminal
HOXC10	homeobox C10	12q13.13	3226	1.03	3.77	1.80E-04	9.04E-05	up Basal vs. Luminal
KRT5	keratin 5	12q13.13	3852	1.32	3.75	1.89E-04	9.47E-05	up Basal vs. Luminal
STAC2	SH3 and cysteine rich domain 2	17q12	342667	1.22	3.62	3.13E-04	1.51E-04	up Basal vs. Luminal
KRT17	keratin 17	17q21.2	3872	1.11	3.52	4.56E-04	2.14E-04	up Basal vs. Luminal
SLC34A2	solute carrier family 34 member 2	4p15.2	10568	1.06	3.35	8.42E-04	3.75E-04	up Basal vs. Luminal
PIGR	polymeric immunoglobulin receptor	1q32.1	5284	1.03	3.22	1.37E-03	5.85E-04	up Basal vs. Luminal
MUCL1	mucin like 1	12q13.2	118430	1.25	3.15	1.71E-03	7.19E-04	up Basal vs. Luminal
SCGB2A2	secretoglobin family 2A member 2	11q12.3	4250	-1.08	-2.34	1.96E-02	6.70E-03	down Basal vs. Luminal
UGT2B11	UDP glucuronosyltransferase family 2 mem	4q13.2	10720	-1.03	-2.69	7.43E-03	2.77E-03	down Basal vs. Luminal
SPAG6	sperm associated antigen 6	10p12.2	9576	-1.03	-3.09	2.05E-03	8.50E-04	down Basal vs. Luminal
CST1	cystatin SN	20p11.21	1469	-1.04	-3.20	1.45E-03	6.18E-04	down Basal vs. Luminal
CYP2A6	cytochrome P450 family 2 subfamily A mer	19q13.2	1548	-1.21	-3.22	1.37E-03	5.84E-04	down Basal vs. Luminal
SLC30A8	solute carrier family 30 member 8	8q24.11	169026	-1.28	-3.31	9.98E-04	4.38E-04	down Basal vs. Luminal
CEACAM5	carcinoembryonic antigen related cell adhe	19q13.2	1048	-1.09	-3.41	6.91E-04	3.13E-04	down Basal vs. Luminal
VSTM2A	V-set and transmembrane domain containi	7p11.2	222008	-1.38	-3.51	4.72E-04	2.21E-04	down Basal vs. Luminal
LRRC31	leucine rich repeat containing 31	3q26.2	79782	-1.07	-3.53	4.44E-04	2.09E-04	down Basal vs. Luminal
ROBO2	roundabout guidance receptor 2	3p12.3	6092	-1.04	-3.68	2.53E-04	1.24E-04	down Basal vs. Luminal
CYP2A7	cytochrome P450 family 2 subfamily A mer	19q13.2	1549	-1.12	-3.72	2.21E-04	1.09E-04	down Basal vs. Luminal
DLX2	distal-less homeobox 2	2q31.1	1746	-1.03	-3.73	2.11E-04	1.05E-04	down Basal vs. Luminal
PIP	prolactin induced protein	7q34	5304	-1.48	-3.83	1.41E-04	7.23E-05	down Basal vs. Luminal
ASCL1	achaete-scute family bHLH transcription fa	12q23.2	429	-1.27	-3.85	1.30E-04	6.71E-05	down Basal vs. Luminal
WDR72	WD repeat domain 72	15q21.3	256764	-1.08	-3.86	1.25E-04	6.48E-05	down Basal vs. Luminal
IGSF1	immunoglobulin superfamily member 1	Xq26.1	3547	-1.25	-4.03	6.34E-05	3.47E-05	down Basal vs. Luminal
GRIA1	glutamate ionotropic receptor AMPA type s	5q33.2	2890	-1.05	-4.07	5.31E-05	2.95E-05	down Basal vs. Luminal
CARTPT	CART prepropeptide	5q13.2	9607	-1.51	-4.14	3.95E-05	2.25E-05	down Basal vs. Luminal
SLC1A1	solute carrier family 1 member 1	9p24.2	6505	-1.06	-4.15	3.73E-05	2.13E-05	down Basal vs. Luminal
PCDHA12	protocadherin alpha 12	5q31.3	56137	-1.09	-4.25	2.45E-05	1.45E-05	down Basal vs. Luminal
SLC7A4	solute carrier family 7 member 4	22q11.21	6545	-1.26	-4.34	1.65E-05	1.01E-05	down Basal vs. Luminal
IGDCC3	immunoglobulin superfamily DCC subclass	15q22.31	9543	-1.14	-4.34	1.63E-05	9.92E-06	down Basal vs. Luminal
GRM4	glutamate metabotropic receptor 4	6p21.31	2914	-1.07	-4.39	1.31E-05	8.14E-06	down Basal vs. Luminal
ZNF385B	zinc finger protein 385B	2q31.2-q31.3	151126	-1.16	-4.47	9.13E-06	5.80E-06	down Basal vs. Luminal
CAPSL	calcyphosine like	5p13.2	133690	-1.02	-4.48	9.02E-06	5.74E-06	down Basal vs. Luminal
CLIC6	chloride intracellular channel 6	21q22.12	54102	-1.45	-4.50	8.22E-06	5.27E-06	down Basal vs. Luminal
PLAAT5	phospholipase A and acyltransferase 5	11q12.3	117245	-1.04	-4.51	7.80E-06	5.03E-06	down Basal vs. Luminal
ATRNL1	attractin like 1	10q25.3	26033	-1.23	-4.64	4.23E-06	2.85E-06	down Basal vs. Luminal
CYP4F11	cytochrome P450 family 4 subfamily F mer	19p13.12	57834	-1.03	-4.67	3.59E-06	2.45E-06	down Basal vs. Luminal
DIO1	iodothyronine deiodinase 1	1p32.3	1733	-1.39	-4.77	2.31E-06	1.62E-06	down Basal vs. Luminal
GPM6A	glycoprotein M6A	4q34.2	2823	-1.13	-4.78	2.13E-06	1.50E-06	down Basal vs. Luminal
BRINP2	BMP/retinoic acid inducible neural specific 2	1q25.2	57795	-1.36	-4.79	2.04E-06	1.45E-06	down Basal vs. Luminal
SPAG17	sperm associated antigen 17	1p12	200162	-1.27	-4.84	1.65E-06	1.19E-06	down Basal vs. Luminal
CFAP221	cilia and flagella associated protein 221	2q14.2	200373	-1.01	-4.86	1.45E-06	1.05E-06	down Basal vs. Luminal
ABCC11	ATP binding cassette subfamily C member	16q12.1	85320	-1.33	-4.90	1.23E-06	9.02E-07	down Basal vs. Luminal
CPB1	carboxypeptidase B1	3q24	1360	-2.49	-4.93	1.07E-06	7.91E-07	down Basal vs. Luminal
SYT1	synaptotagmin 1	12q21.2	6857	-1.32	-4.94	9.84E-07	7.31E-07	down Basal vs. Luminal
PWWP3B	PWWP domain containing 3B	Xq22.3	139221	-1.03	-4.95	9.35E-07	6.97E-07	down Basal vs. Luminal
CXADRP3	CXADR pseudogene 3	18p11.21	440224	-1.31	-5.00	7.48E-07	5.68E-07	down Basal vs. Luminal
PAH	phenylalanine hydroxylase	12q23.2	5053	-1.19	-5.02	6.59E-07	5.05E-07	down Basal vs. Luminal
RP1	RP1 axonemal microtubule associated	8q11.23-q12.1	6101	-1.08	-5.04	5.95E-07	4.59E-07	down Basal vs. Luminal
KCNC2	potassium voltage-gated channel subfamily	12q21.1	3747	-1.84	-5.05	5.83E-07	4.51E-07	down Basal vs. Luminal
GLRA3	glycine receptor alpha 3	4q34.1	8001	-1.57	-5.07	5.21E-07	4.06E-07	down Basal vs. Luminal
ATP1A2	ATPase Na+/K+ transporting subunit alpha	1q23.2	477	-1.03	-5.09	4.60E-07	3.62E-07	down Basal vs. Luminal
NLRP5	NLR family pyrin domain containing 5	19q13.43	126206	-1.28	-5.10	4.51E-07	3.56E-07	down Basal vs. Luminal
NELL2	neural EGFL like 2	12q12	4753	-1.25	-5.11	4.32E-07	3.41E-07	down Basal vs. Luminal
FAM184B	family with sequence similarity 184 membe	4p15.32-p15.31	27146	-1.17	-5.13	3.94E-07	3.14E-07	down Basal vs. Luminal
SNORD116-4	small nucleolar RNA, C/D box 116-4	15q11.2	100033416	-1.03	-5.16	3.33E-07	2.68E-07	down Basal vs. Luminal
KLHDC7A	kelch domain containing 7A	1p36.13	127707	-1.48	-5.26	1.91E-07	1.61E-07	down Basal vs. Luminal
HEPACAM2	HEPACAM family member 2	7q21.2	253012	-1.37	-5.29	1.70E-07	1.44E-07	down Basal vs. Luminal
ARMC3	armadillo repeat containing 3	10p12.2	219681	-1.11	-5.29	1.68E-07	1.43E-07	down Basal vs. Luminal
CYP4F22	cytochrome P450 family 4 subfamily F mer	19p13.12	126410	-1.45	-5.37	1.12E-07	9.87E-08	down Basal vs. Luminal
BPIFB1	BPI fold containing family B member 1	20q11.21	92747	-1.49	-5.45	7.27E-08	6.55E-08	down Basal vs. Luminal
ENTPD8	ectonucleoside triphosphate diphosphohydr	9q34.3	377841	-1.03	-5.45	7.18E-08	6.48E-08	down Basal vs. Luminal
PYY	peptide YY	17q21.31	5697	-1.18	-5.46	6.92E-08	6.26E-08	down Basal vs. Luminal
UGT2B15	UDP glucuronosyltransferase family 2 mem	4q13.2	7366	-1.38	-5.48	6.24E-08	5.69E-08	down Basal vs. Luminal
IL20	interleukin 20	1q32.1	50604	-1.45	-5.48	6.08E-08	5.55E-08	down Basal vs. Luminal
SCN7A	sodium voltage-gated channel alpha subun	2q24.3	6332	-1.15	-5.49	5.92E-08	5.41E-08	down Basal vs. Luminal
MMP10	matrix metallopeptidase 10	11q22.2	4319	-1.30	-5.49	5.87E-08	5.37E-08	down Basal vs. Luminal
CDHR3	cadherin related family member 3	7q22.3	222256	-1.12	-5.49	5.70E-08	5.23E-08	down Basal vs. Luminal
PLA2G10	phospholipase A2 group X	16p13.12	8399	-1.05	-5.52	5.04E-08	4.65E-08	down Basal vs. Luminal
FLT3	fms related tyrosine kinase 3	13q12.2	2322	-1.32	-5.54	4.48E-08	4.17E-08	down Basal vs. Luminal
TRH	thyrotropin releasing hormone	3q22.1	7200	-1.60	-5.58	3.54E-08	3.36E-08	down Basal vs. Luminal
ABCC13	ATP binding cassette subfamily C member	21q11.2	150000	-1.69	-5.62	2.86E-08	2.76E-08	down Basal vs. Luminal
ACOX2	acyl-CoA oxidase 2	3p14.3	8309	-1.01	-5.66	2.29E-08	2.24E-08	down Basal vs. Luminal
MCCD1	mitochondrial coiled-coil domain 1	6p21.33	401250	-1.27	-5.67	2.11E-08	2.07E-08	down Basal vs. Luminal
CABCOCO1	ciliary associated calcium binding coiled-co	10q21.2	219621	-1.06	-5.69	1.96E-08	1.94E-08	down Basal vs. Luminal
FAM135B	family with sequence similarity 135 membe	8q24.23	51059	-1.25	-5.69	1.90E-08	1.88E-08	down Basal vs. Luminal
SYNPO2L	synaptopodin 2 like	10q22.2	79933	-1.54	-5.75	1.41E-08	1.43E-08	down Basal vs. Luminal
NPY5R	neuropeptide Y receptor Y5	4q32.2	4889	-1.54	-5.75	1.40E-08	1.42E-08	down Basal vs. Luminal
NRIP3	nuclear receptor interacting protein 3	11p15.4	56675	-1.25	-5.80	1.06E-08	1.11E-08	down Basal vs. Luminal
NEURL1	neuralized E3 ubiquitin protein ligase 1	10q24.33	9148	-1.25	-5.82	9.12E-09	9.63E-09	down Basal vs. Luminal
DCLK1	doublecortin like kinase 1	13q13.3	9201	-1.08	-5.84	8.11E-09	8.64E-09	down Basal vs. Luminal
CST5	cystatin D	20p11.21	1473	-1.91	-5.85	7.96E-09	8.49E-09	down Basal vs. Luminal
TMEM145	transmembrane protein 145	19q13.2	284339	-1.21	-5.85	7.70E-09	8.23E-09	down Basal vs. Luminal
CCDC65	coiled-coil domain containing 65	12q13.12	85478	-1.01	-5.86	7.41E-09	7.93E-09	down Basal vs. Luminal
SYT13	synaptotagmin 13	11p11.2	57586	-2.01	-5.87	6.93E-09	7.46E-09	down Basal vs. Luminal
FGF10	fibroblast growth factor 10	5p12	2255	-1.52	-5.89	6.24E-09	6.78E-09	down Basal vs. Luminal
FLRT3	fibronectin leucine rich transmembrane pro	20p12.1	23767	-1.21	-5.90	6.04E-09	6.57E-09	down Basal vs. Luminal
CACNG4	calcium voltage-gated channel auxiliary sul	17q24.2	27092	-1.21	-5.91	5.60E-09	6.12E-09	down Basal vs. Luminal
PPP1R3C	protein phosphatase 1 regulatory subunit 3(	10q23.32	5507	-1.04	-5.92	5.14E-09	5.66E-09	down Basal vs. Luminal
TSPAN1	tetraspanin 1	1p34.1	10103	-1.10	-5.94	4.70E-09	5.22E-09	down Basal vs. Luminal
CACNA1F	calcium voltage-gated channel subunit alph	Xp11.23	778	-1.09	-5.96	4.16E-09	4.66E-09	down Basal vs. Luminal
F7	coagulation factor VII	13q34	2155	-1.29	-5.97	3.98E-09	4.48E-09	down Basal vs. Luminal
WDR17	WD repeat domain 17	4q34.2	116966	-1.28	-6.00	3.33E-09	3.81E-09	down Basal vs. Luminal
CAPN9	calpain 9	1q42.2	10753	-1.25	-6.01	3.08E-09	3.54E-09	down Basal vs. Luminal
MYT1	myelin transcription factor 1	20q13.33	4661	-1.52	-6.02	2.95E-09	3.40E-09	down Basal vs. Luminal
LRP2	LDL receptor related protein 2	2q31.1	4036	-1.84	-6.07	2.13E-09	2.52E-09	down Basal vs. Luminal
AZU1	azurocidin 1	19p13.3	566	-1.17	-6.13	1.52E-09	1.85E-09	down Basal vs. Luminal
DHRS2	dehydrogenase/reductase 2	14q11.2	10202	-2.15	-6.15	1.40E-09	1.71E-09	down Basal vs. Luminal
LAMA3	laminin subunit alpha 3	18q11.2	3909	-1.06	-6.15	1.34E-09	1.64E-09	down Basal vs. Luminal
GRIA2	glutamate ionotropic receptor AMPA type s	4q32.1	2891	-2.36	-6.16	1.26E-09	1.56E-09	down Basal vs. Luminal
EEF1A2	eukaryotic translation elongation factor 1 al	20q13.33	1917	-1.54	-6.17	1.19E-09	1.48E-09	down Basal vs. Luminal
PRKAA2	protein kinase AMP-activated catalytic subu	1p32.2	5563	-1.29	-6.17	1.19E-09	1.47E-09	down Basal vs. Luminal
PSD3	pleckstrin and Sec7 domain containing 3	8p22	23362	-1.12	-6.21	9.46E-10	1.19E-09	down Basal vs. Luminal
PDZK1P1	PDZ domain containing 1 pseudogene 1	1q21.2	100034743	-1.29	-6.23	8.47E-10	1.08E-09	down Basal vs. Luminal
CCDC160	coiled-coil domain containing 160	Xq26.2	347475	-1.01	-6.24	7.79E-10	9.97E-10	down Basal vs. Luminal
HPX	hemopexin	11p15.4	3263	-1.37	-6.30	5.48E-10	7.17E-10	down Basal vs. Luminal
IGSF9B	immunoglobulin superfamily member 9B	11q25	22997	-1.05	-6.31	5.10E-10	6.71E-10	down Basal vs. Luminal
GSTM3	glutathione S-transferase mu 3	1p13.3	2947	-1.04	-6.33	4.61E-10	6.11E-10	down Basal vs. Luminal

Symbol	Description	Cytoband	Entrez Gene ID	Basal vs. Luminal (log2-ratio)	t	p-value[1]	q-value	Associated to
SEZ6L	seizure related 6 homolog like	22q12.1	23544	-1.60	-6.34	4.35E-10	5.80E-10	down Basal vs. Luminal
PPP4R4	protein phosphatase 4 regulatory subunit 4	14q32.12-q32.13	57718	-1.68	-6.35	4.08E-10	5.45E-10	down Basal vs. Luminal
BCAS1	breast carcinoma amplified sequence 1	20q13.2	8537	-1.31	-6.37	3.68E-10	4.96E-10	down Basal vs. Luminal
PLAT	plasminogen activator, tissue type	8p11.21	5327	-1.01	-6.46	2.01E-10	2.85E-10	down Basal vs. Luminal
RGL3	ral guanine nucleotide dissociation stimulat	19p13.2	57139	-1.06	-6.49	1.71E-10	2.44E-10	down Basal vs. Luminal
NBPF6	NBPF member 6	1p13.3	653149	-1.89	-6.49	1.70E-10	2.43E-10	down Basal vs. Luminal
NBPF4	NBPF member 4	1p13.3	148545	-1.91	-6.53	1.31E-10	1.90E-10	down Basal vs. Luminal
PEX5L	peroxisomal biogenesis factor 5 like	3q26.33	51555	-1.46	-6.55	1.17E-10	1.71E-10	down Basal vs. Luminal
TPSG1	tryptase gamma 1	16p13.3	25823	-1.65	-6.62	7.71E-11	1.17E-10	down Basal vs. Luminal
CYP2B6	cytochrome P450 family 2 subfamily B mer	19q13.2	1555	-1.16	-6.62	7.61E-11	1.15E-10	down Basal vs. Luminal
IGFALS	insulin like growth factor binding protein aci	16p13.3	3483	-1.29	-6.66	5.95E-11	9.12E-11	down Basal vs. Luminal
GRIK4	glutamate ionotropic receptor kainate type s	11q23.3	2900	-1.25	-6.66	5.93E-11	9.10E-11	down Basal vs. Luminal
GP2	glycoprotein 2	16p12.3	2813	-1.94	-6.68	5.20E-11	8.07E-11	down Basal vs. Luminal
KCNE4	potassium voltage-gated channel subfamily	2q36.1	23704	-1.38	-6.68	5.19E-11	8.06E-11	down Basal vs. Luminal
ACMSD	aminocarboxymuconate semialdehyde dec	2q21.3	130013	-1.52	-6.69	4.88E-11	7.64E-11	down Basal vs. Luminal
AKR7A3	aldo-keto reductase family 7 member A3	1p36.13	22977	-1.45	-6.72	3.97E-11	6.29E-11	down Basal vs. Luminal
CFAP46	cilia and flagella associated protein 46	10q26.3	54777	-1.22	-6.76	3.10E-11	5.02E-11	down Basal vs. Luminal
LRG1	leucine rich alpha-2-glycoprotein 1	19p13.3	116844	-1.59	-6.79	2.50E-11	4.12E-11	down Basal vs. Luminal
SLC7A2	solute carrier family 7 member 2	8p22	6542	-1.63	-6.81	2.30E-11	3.81E-11	down Basal vs. Luminal
NUP62CL	nucleoporin 62 C-terminal like	Xq22.3	54830	-1.01	-6.89	1.34E-11	2.29E-11	down Basal vs. Luminal
CCDC196	coiled-coil domain containing 196	14q23.3	440184	-1.34	-6.90	1.22E-11	2.10E-11	down Basal vs. Luminal
REEP6	receptor accessory protein 6	19p13.3	92840	-1.05	-6.92	1.07E-11	1.87E-11	down Basal vs. Luminal
MYRIP	myosin VIIA and Rab interacting protein	3p22.1	25924	-1.06	-6.93	9.97E-12	1.75E-11	down Basal vs. Luminal
DPY19L2P4	DPY19L2 pseudogene 4	7q21.13	442523	-1.61	-6.95	8.72E-12	1.54E-11	down Basal vs. Luminal
LYPD6B	LY6/PLAUR domain containing 6B	2q23.2	130576	-1.16	-6.97	7.75E-12	1.38E-11	down Basal vs. Luminal
MISP	mitotic spindle positioning	19p13.3	126353	-1.14	-7.00	6.31E-12	1.14E-11	down Basal vs. Luminal
STK32B	serine/threonine kinase 32B	4p16.2	55351	-1.31	-7.04	5.10E-12	9.40E-12	down Basal vs. Luminal
TRIM58	tripartite motif containing 58	1q44	25893	-1.45	-7.04	5.07E-12	9.37E-12	down Basal vs. Luminal
NOVA1	NOVA alternative splicing regulator 1	14q12	4857	-1.57	-7.07	4.07E-12	7.65E-12	down Basal vs. Luminal
MS4A8	membrane spanning 4-domains A8	11q12.2	83661	-1.73	-7.08	3.84E-12	7.24E-12	down Basal vs. Luminal
MYCBPAP	MYCBP associated protein	17q21.33	84073	-1.02	-7.08	3.72E-12	7.03E-12	down Basal vs. Luminal
FAM57B	family with sequence similarity 57 member	16p11.2	83723	-1.25	-7.10	3.41E-12	6.48E-12	down Basal vs. Luminal
DEFB132	defensin beta 132	20p13	400830	-1.23	-7.15	2.36E-12	4.63E-12	down Basal vs. Luminal
LOC285593	uncharacterized LOC285593	5q35.2	285593	-1.18	-7.16	2.28E-12	4.48E-12	down Basal vs. Luminal
CADM2	cell adhesion molecule 2	3p12.1	253559	-1.29	-7.17	2.02E-12	4.00E-12	down Basal vs. Luminal
NXPH3	neurexophilin 3	17q21.33	11248	-1.04	-7.18	1.98E-12	3.94E-12	down Basal vs. Luminal
DCDC2	doublecortin domain containing 2	6p22.3	51473	-1.69	-7.19	1.77E-12	3.55E-12	down Basal vs. Luminal
REEP1	receptor accessory protein 1	2p11.2	65055	-1.11	-7.20	1.71E-12	3.42E-12	down Basal vs. Luminal
SLITRK6	SLIT and NTRK like family member 6	13q31.1	84189	-2.20	-7.22	1.49E-12	3.01E-12	down Basal vs. Luminal
SORCS1	sortilin related VPS10 domain containing re	10q25.1	114815	-2.12	-7.23	1.40E-12	2.84E-12	down Basal vs. Luminal
TMC3	transmembrane channel like 3	15q25.1	342125	-1.60	-7.24	1.30E-12	2.65E-12	down Basal vs. Luminal
PHYHD1	phytanoyl-CoA dioxygenase domain contai	9q34.11	254295	-1.40	-7.24	1.30E-12	2.64E-12	down Basal vs. Luminal
LINC00160	long intergenic non-protein coding RNA 16(	21q22.12	54064	-1.07	-7.25	1.22E-12	2.50E-12	down Basal vs. Luminal
AGTR1	angiotensin II receptor type 1	3q24	185	-1.96	-7.29	9.16E-13	1.92E-12	down Basal vs. Luminal
CNTD2	cyclin N-terminal domain containing 2	19q13.2	79935	-1.31	-7.29	9.07E-13	1.90E-12	down Basal vs. Luminal
TUBA3C	tubulin alpha 3c	13q12.11	7278	-1.37	-7.31	8.01E-13	1.69E-12	down Basal vs. Luminal
WNK4	WNK lysine deficient protein kinase 4	17q21.2	65266	-1.76	-7.32	7.22E-13	1.54E-12	down Basal vs. Luminal
SPEF1	sperm flagellar 1	20p13	25876	-1.16	-7.35	6.12E-13	1.32E-12	down Basal vs. Luminal
SERPINA6	serpin family A member 6	14q32.13	866	-2.99	-7.37	5.20E-13	1.14E-12	down Basal vs. Luminal
LINC00472	long intergenic non-protein coding RNA 47	6q13	79940	-1.22	-7.40	4.37E-13	9.70E-13	down Basal vs. Luminal
CLGN	calmegin	4q31.1	1047	-1.52	-7.40	4.19E-13	9.33E-13	down Basal vs. Luminal
MYO18B	myosin XVIIIB	22q12.1	84700	-1.27	-7.41	3.93E-13	8.79E-13	down Basal vs. Luminal
FRMPD2	FERM and PDZ domain containing 2	10q11.22	143162	-1.13	-7.43	3.37E-13	7.59E-13	down Basal vs. Luminal
LRGUK	leucine rich repeats and guanylate kinase c	7q33	136332	-1.01	-7.44	3.19E-13	7.24E-13	down Basal vs. Luminal
CA14	carbonic anhydrase 14	1q21.2	23632	-1.11	-7.47	2.65E-13	6.09E-13	down Basal vs. Luminal
SLC6A4	solute carrier family 6 member 4	17q11.2	6532	-2.41	-7.48	2.39E-13	5.55E-13	down Basal vs. Luminal
COX6C	cytochrome c oxidase subunit 6C	8q22.2	1345	-1.09	-7.50	2.08E-13	4.88E-13	down Basal vs. Luminal
CST2	cystatin SA	20p11.21	1470	-1.58	-7.55	1.45E-13	3.53E-13	down Basal vs. Luminal
SPTBN4	spectrin beta, non-erythrocytic 4	19q13.2	57731	-1.14	-7.56	1.36E-13	3.33E-13	down Basal vs. Luminal
SNORD116-28	small nucleolar RNA, C/D box 116-28	15q11.2	100033820	-1.11	-7.60	1.02E-13	2.54E-13	down Basal vs. Luminal
CHST8	carbohydrate sulfotransferase 8	19q13.11	64377	-1.94	-7.62	9.27E-14	2.33E-13	down Basal vs. Luminal
FGD3	FYVE, RhoGEF and PH domain containing	9q22.31	89846	-1.09	-7.62	9.00E-14	2.27E-13	down Basal vs. Luminal
RBM11	RNA binding motif protein 11	21q11.2	54033	-1.13	-7.65	7.31E-14	1.88E-13	down Basal vs. Luminal
ARFGEF3	ARFGEF family member 3	6q23.3-q24.1	57221	-1.00	-7.65	7.29E-14	1.88E-13	down Basal vs. Luminal
ANKRD30B	ankyrin repeat domain 30B	18p11.21	374860	-2.01	-7.69	5.41E-14	1.42E-13	down Basal vs. Luminal
RET	ret proto-oncogene	10q11.21	5979	-1.62	-7.71	4.77E-14	1.27E-13	down Basal vs. Luminal
ABLIM3	actin binding LIM protein family member 3	5q32	22885	-1.02	-7.73	4.29E-14	1.15E-13	down Basal vs. Luminal
AZGP1	alpha-2-glycoprotein 1, zinc-binding	7q22.1	563	-1.34	-7.73	4.22E-14	1.14E-13	down Basal vs. Luminal
PHGR1	proline, histidine and glycine rich 1	15q15.1	644844	-1.96	-7.79	2.72E-14	7.53E-14	down Basal vs. Luminal
KCNJ3	potassium voltage-gated channel subfamily	2q24.1	3760	-3.02	-7.85	1.80E-14	5.15E-14	down Basal vs. Luminal
FBXO15	F-box protein 15	18q22.3	201456	-1.07	-7.87	1.51E-14	4.34E-14	down Basal vs. Luminal
C9orf152	chromosome 9 open reading frame 152	9q31.3	401546	-1.16	-7.92	1.08E-14	3.19E-14	down Basal vs. Luminal
GIPR	gastric inhibitory polypeptide receptor	19q13.32	2696	-1.16	-7.97	7.28E-15	2.21E-14	down Basal vs. Luminal
CCDC74A	coiled-coil domain containing 74A	2q21.1	90557	-1.33	-7.99	6.42E-15	1.96E-14	down Basal vs. Luminal
PIEZO2	piezo type mechanosensitive ion channel c	18p11.22-p11.21	63895	-1.50	-8.04	4.47E-15	1.41E-14	down Basal vs. Luminal
FGFR3	fibroblast growth factor receptor 3	4p16.3	2261	-1.53	-8.04	4.38E-15	1.38E-14	down Basal vs. Luminal
NOSTRIN	nitric oxide synthase trafficking	2q24.3	115677	-1.05	-8.06	3.81E-15	1.22E-14	down Basal vs. Luminal
GPR139	G protein-coupled receptor 139	16p12.3	124274	-1.41	-8.07	3.46E-15	1.12E-14	down Basal vs. Luminal
PLEKHD1	pleckstrin homology and coiled-coil domai	14q24.1	400224	-1.53	-8.07	3.39E-15	1.10E-14	down Basal vs. Luminal
GRPR	gastrin releasing peptide receptor	Xp22.2	2925	-2.14	-8.08	3.21E-15	1.05E-14	down Basal vs. Luminal
SPATA46	spermatogenesis associated 46	1q23.3	284680	-1.01	-8.08	3.14E-15	1.03E-14	down Basal vs. Luminal
SRMS	src-related kinase lacking C-terminal regul	20q13.33	6725	-1.03	-8.14	1.99E-15	6.70E-15	down Basal vs. Luminal
SLC1A2	solute carrier family 1 member 2	11p13	6506	-1.41	-8.15	1.86E-15	6.28E-15	down Basal vs. Luminal
SERPINA5	serpin family A member 5	14q32.13	5104	-1.99	-8.21	1.17E-15	4.06E-15	down Basal vs. Luminal
FAM234B	family with sequence similarity 234 membe	12p13.1	57613	-1.15	-8.24	9.75E-16	3.44E-15	down Basal vs. Luminal
EGOT	eosinophil granule ontogeny transcript	3p26.1	100126791	-1.30	-8.24	9.36E-16	3.31E-15	down Basal vs. Luminal
BMPR1B	bone morphogenetic protein receptor type	4q22.3	658	-2.74	-8.25	9.21E-16	3.26E-15	down Basal vs. Luminal
RSPH1	radial spoke head component 1	21q22.3	89765	-1.05	-8.26	8.08E-16	2.88E-15	down Basal vs. Luminal
KRT37	keratin 37	17q21.2	8688	-1.31	-8.27	7.77E-16	2.78E-15	down Basal vs. Luminal
RPS6KA6	ribosomal protein S6 kinase A6	Xq21.1	27330	-1.39	-8.27	7.43E-16	2.67E-15	down Basal vs. Luminal
COL4A5	collagen type IV alpha 5 chain	Xq22.3	1287	-1.19	-8.29	6.82E-16	2.47E-15	down Basal vs. Luminal
EWSAT1	Ewing sarcoma associated transcript 1	15q23	283673	-1.56	-8.30	6.06E-16	2.21E-15	down Basal vs. Luminal
KLHL11	kelch like family member 11	17q21.2	55175	-1.08	-8.33	5.06E-16	1.87E-15	down Basal vs. Luminal
TFF1	trefoil factor 1	21q22.3	7031	-2.64	-8.36	3.85E-16	1.45E-15	down Basal vs. Luminal
SPATA6L	spermatogenesis associated 6 like	9p24.2-p24.1	55064	-1.19	-8.39	3.06E-16	1.17E-15	down Basal vs. Luminal
PMAIP1	phorbol-12-myristate-13-acetate-induced p	18q21.32	5366	-1.05	-8.44	2.11E-16	8.28E-16	down Basal vs. Luminal
MED12L	mediator complex subunit 12 like	3q25.1	116931	-1.31	-8.45	1.90E-16	7.48E-16	down Basal vs. Luminal
INSYN2A	inhibitory synaptic factor 2A	10q26.2	642938	-2.22	-8.48	1.58E-16	6.27E-16	down Basal vs. Luminal
C1orf226	chromosome 1 open reading frame 226	1q23.3	400793	-1.06	-8.50	1.28E-16	5.15E-16	down Basal vs. Luminal
PLIN5	perilipin 5	19p13.3	440503	-1.75	-8.54	9.64E-17	3.93E-16	down Basal vs. Luminal
FANK1	fibronectin type III and ankyrin repeat doma	10q26.2	92565	-1.00	-8.54	9.56E-17	3.90E-16	down Basal vs. Luminal
TMEM232	transmembrane protein 232	5q22.1	642987	-1.29	-8.57	7.92E-17	3.26E-16	down Basal vs. Luminal
CAPN13	calpain 13	2p23.1	92291	-1.30	-8.58	7.01E-17	2.91E-16	down Basal vs. Luminal
PTGER3	prostaglandin E receptor 3	1p31.1	5733	-1.47	-8.65	3.98E-17	1.72E-16	down Basal vs. Luminal
NBEA	neurobeachin	13q13.3	26960	-1.03	-8.68	3.22E-17	1.42E-16	down Basal vs. Luminal
CYP2B7P	cytochrome P450 family 2 subfamily B mer	19q13.2	1556	-2.82	-8.69	2.95E-17	1.31E-16	down Basal vs. Luminal
PPM1J	protein phosphatase, Mg2+/Mn2+ depende	1p13.2	333926	-1.06	-8.72	2.32E-17	1.05E-16	down Basal vs. Luminal
ACER2	alkaline ceramidase 2	9p22.1	340485	-1.08	-8.73	2.23E-17	1.01E-16	down Basal vs. Luminal
HIST4H4	histone cluster 4 H4	12p12.3	121504	-1.11	-8.76	1.73E-17	7.96E-17	down Basal vs. Luminal
ZNF703	zinc finger protein 703	8p11.23	80139	-1.13	-8.76	1.68E-17	7.78E-17	down Basal vs. Luminal
SRARP	steroid receptor associated and regulated p	1p36.13	149563	-2.33	-8.76	1.66E-17	7.67E-17	down Basal vs. Luminal
ZG16B	zymogen granule protein 16B	16p13.3	124220	-1.01	-8.79	1.38E-17	6.51E-17	down Basal vs. Luminal
PLPPR3	phospholipid phosphatase related 3	19p13.3	79948	-1.99	-8.80	1.28E-17	6.05E-17	down Basal vs. Luminal
GLIPR1L2	GLIPR1 like 2	12q21.2	144321	-1.02	-8.81	1.15E-17	5.47E-17	down Basal vs. Luminal
WDR31	WD repeat domain 31	9q32	114987	-1.03	-8.83	9.97E-18	4.82E-17	down Basal vs. Luminal
TTC36	tetratricopeptide repeat domain 36	11q23.3	143941	-1.72	-8.88	6.64E-18	3.27E-17	down Basal vs. Luminal
KIF5C	kinesin family member 5C	2q23.1-q23.2	3800	-1.47	-8.88	6.55E-18	3.23E-17	down Basal vs. Luminal
ZNF214	zinc finger protein 214	11p15.4	7761	-1.18	-8.92	4.85E-18	2.44E-17	down Basal vs. Luminal

Symbol	Description	Cytoband	Entrez Gene ID	Basal vs. Luminal (log2-ratio)	t	p-value[1]	q-value	Associated to
GRIK3	glutamate ionotropic receptor kainate type s	1p34.3	2899	-1.89	-8.97	3.09E-18	1.60E-17	down Basal vs. Luminal
PCLO	piccolo presynaptic cytomatrix protein	7q21.11	27445	-1.04	-8.99	2.66E-18	1.39E-17	down Basal vs. Luminal
ARNT2	aryl hydrocarbon receptor nuclear transloca	15q25.1	9915	-1.29	-9.00	2.48E-18	1.30E-17	down Basal vs. Luminal
KCNH1	potassium voltage-gated channel subfamily	1q32.2	3756	-1.74	-9.02	2.21E-18	1.16E-17	down Basal vs. Luminal
ST8SIA6	ST8 alpha-N-acetyl-neuraminide alpha-2,8	10p12.33	338596	-1.70	-9.02	2.12E-18	1.12E-17	down Basal vs. Luminal
SEMA3B	semaphorin 3B	3p21.31	7869	-1.43	-9.04	1.84E-18	9.77E-18	down Basal vs. Luminal
ADGRB2	adhesion G protein-coupled receptor B2	1p35.2	576	-1.24	-9.05	1.66E-18	8.84E-18	down Basal vs. Luminal
SEC14L2	SEC14 like lipid binding 2	22q12.2	23541	-1.49	-9.11	9.91E-19	5.46E-18	down Basal vs. Luminal
SCUBE1	signal peptide, CUB domain and EGF like d	22q13.2	80274	-1.91	-9.13	8.59E-19	4.78E-18	down Basal vs. Luminal
C1orf21	chromosome 1 open reading frame 21	1q25.3	81563	-1.00	-9.14	8.20E-19	4.58E-18	down Basal vs. Luminal
KSR2	kinase suppressor of ras 2	12q24.22-q24.23	283455	-1.19	-9.19	5.51E-19	3.15E-18	down Basal vs. Luminal
VAV3	vav guanine nucleotide exchange factor 3	1p13.3	10451	-1.01	-9.19	5.18E-19	2.99E-18	down Basal vs. Luminal
POLN	DNA polymerase nu	4p16.3	353497	-1.05	-9.20	4.81E-19	2.80E-18	down Basal vs. Luminal
RAB30	RAB30, member RAS oncogene family	11q14.1	27314	-1.21	-9.20	4.74E-19	2.77E-18	down Basal vs. Luminal
DYNLRB2	dynein light chain roadblock-type 2	16q23.2	83657	-1.18	-9.22	4.25E-19	2.50E-18	down Basal vs. Luminal
PCP2	Purkinje cell protein 2	19p13.2	126006	-1.38	-9.25	3.14E-19	1.90E-18	down Basal vs. Luminal
TOX3	TOX high mobility group box family membe	16q12.1	27324	-1.75	-9.32	1.90E-19	1.19E-18	down Basal vs. Luminal
SPDEF	SAM pointed domain containing ETS transc	6p21.31	25803	-1.23	-9.32	1.88E-19	1.18E-18	down Basal vs. Luminal
TFF3	trefoil factor 3	21q22.3	7033	-2.25	-9.35	1.47E-19	9.35E-19	down Basal vs. Luminal
FMN1	formin 1	15q13.3	342184	-1.35	-9.35	1.37E-19	8.75E-19	down Basal vs. Luminal
CST9	cystatin 9	20p11.21	128822	-3.10	-9.36	1.26E-19	8.13E-19	down Basal vs. Luminal
C16orf71	chromosome 16 open reading frame 71	16p13.3	146562	-1.09	-9.40	9.34E-20	6.08E-19	down Basal vs. Luminal
DOK7	docking protein 7	4p16.3	285489	-1.87	-9.43	7.11E-20	4.72E-19	down Basal vs. Luminal
NOS1AP	nitric oxide synthase 1 adaptor protein	1q23.3	9722	-1.39	-9.44	6.87E-20	4.59E-19	down Basal vs. Luminal
SERPINA3	serpin family A member 3	14q32.13	12	-1.63	-9.44	6.66E-20	4.46E-19	down Basal vs. Luminal
MAG	myelin associated glycoprotein	19q13.12	4099	-1.66	-9.46	5.75E-20	3.88E-19	down Basal vs. Luminal
MRPS30	mitochondrial ribosomal protein S30	5p12	10884	-1.33	-9.49	4.49E-20	3.09E-19	down Basal vs. Luminal
RIMS4	regulating synaptic membrane exocytosis 4	20q13.12	140730	-2.29	-9.49	4.38E-20	3.02E-19	down Basal vs. Luminal
ANKRD30A	ankyrin repeat domain 30A	10p11.21	91074	-3.04	-9.50	3.92E-20	2.72E-19	down Basal vs. Luminal
TUBA3D	tubulin alpha 3d	2q21.1	113457	-2.41	-9.52	3.53E-20	2.46E-19	down Basal vs. Luminal
MICALCL	MICAL C-terminal like	11p15.3	84953	-1.30	-9.52	3.36E-20	2.35E-19	down Basal vs. Luminal
EVL	Enah/Vasp-like	14q32.2	51466	-1.03	-9.55	2.61E-20	1.86E-19	down Basal vs. Luminal
AGBL2	ATP/GTP binding protein like 2	11p11.2	79841	-1.09	-9.56	2.50E-20	1.79E-19	down Basal vs. Luminal
CFAP70	cilia and flagella associated protein 70	10q22.2	118491	-1.14	-9.57	2.17E-20	1.58E-19	down Basal vs. Luminal
RLN2	relaxin 2	9p24.1	6019	-1.59	-9.59	1.95E-20	1.43E-19	down Basal vs. Luminal
CACNA2D2	calcium voltage-gated channel auxiliary sub	3p21.31	9254	-1.44	-9.61	1.63E-20	1.21E-19	down Basal vs. Luminal
SCNN1A	sodium channel epithelial 1 alpha subunit	12p13.31	6337	-1.09	-9.63	1.36E-20	1.02E-19	down Basal vs. Luminal
SGK3	serum/glucocorticoid regulated kinase fami	8q13.1	23678	-1.19	-9.64	1.22E-20	9.25E-20	down Basal vs. Luminal
LOC148696	uncharacterized LOC148696	1q32.2	148696	-1.23	-9.68	8.57E-21	6.59E-20	down Basal vs. Luminal
CAPN8	calpain 8	1q41	388743	-2.18	-9.73	5.56E-21	4.39E-20	down Basal vs. Luminal
PGLYRP2	peptidoglycan recognition protein 2	19p13.12	114770	-2.38	-9.74	5.33E-21	4.24E-20	down Basal vs. Luminal
GASK1B	golgi associated kinase 1B	4q32.1	51313	-1.15	-9.74	5.06E-21	4.05E-20	down Basal vs. Luminal
CFAP69	cilia and flagella associated protein 69	7q21.13	79846	-1.07	-9.75	4.80E-21	3.88E-20	down Basal vs. Luminal
CCDC158	coiled-coil domain containing 158	4q21.1	339965	-1.57	-9.77	3.94E-21	3.25E-20	down Basal vs. Luminal
KIAA1324	KIAA1324	1p13.3	57535	-1.06	-9.79	3.34E-21	2.78E-20	down Basal vs. Luminal
PCSK6	proprotein convertase subtilisin/kexin type 6	15q26.3	5046	-1.37	-9.81	2.92E-21	2.47E-20	down Basal vs. Luminal
ELOVL2	ELOVL fatty acid elongase 2	6p24.2	54898	-2.43	-9.82	2.61E-21	2.23E-20	down Basal vs. Luminal
MAATS1	MYCBP associated and testis expressed 1	3q13.33	89876	-1.51	-9.86	1.94E-21	1.68E-20	down Basal vs. Luminal
RETREG1	reticulophagy regulator 1	5p15.1	54463	-1.18	-9.86	1.81E-21	1.58E-20	down Basal vs. Luminal
VWA2	von Willebrand factor A domain containing	10q25.3	340706	-1.95	-9.87	1.79E-21	1.57E-20	down Basal vs. Luminal
CCDC74B	coiled-coil domain containing 74B	2q21.1	91409	-1.48	-9.87	1.73E-21	1.53E-20	down Basal vs. Luminal
RAB19	RAB19, member RAS oncogene family	7q34	401409	-1.09	-9.88	1.53E-21	1.36E-20	down Basal vs. Luminal
ZNF281	zinc finger protein 281	1q32.1	23528	-1.09	-9.92	1.11E-21	9.98E-21	down Basal vs. Luminal
TMPRSS6	transmembrane serine protease 6	22q12.3	164656	-2.31	-9.92	1.09E-21	9.89E-21	down Basal vs. Luminal
ARMT1	acidic residue methyltransferase 1	6q25.1	79624	-1.19	-9.93	1.06E-21	9.60E-21	down Basal vs. Luminal
NPNT	nephronectin	4q24	255743	-1.41	-9.93	1.04E-21	9.47E-21	down Basal vs. Luminal
GLRB	glycine receptor beta	4q32.1	2743	-1.56	-9.99	6.24E-22	5.90E-21	down Basal vs. Luminal
CHAD	chondroadherin	17q21.33	1101	-2.42	-9.99	5.80E-22	5.51E-21	down Basal vs. Luminal
GREB1L	GREB1 like retinoic acid receptor coactivato	18q11.1-q11.2	80000	-1.90	-10.00	5.69E-22	5.43E-21	down Basal vs. Luminal
RASEF	RAS and EF-hand domain containing	9q21.32	158158	-1.55	-10.02	4.60E-22	4.45E-21	down Basal vs. Luminal
WDR93	WD repeat domain 93	15q26.1	56964	-1.14	-10.03	4.10E-22	4.00E-21	down Basal vs. Luminal
SYTL5	synaptotagmin like 5	Xp11.4	94122	-2.15	-10.12	1.98E-22	2.03E-21	down Basal vs. Luminal
ADAMTS15	ADAM metallopeptidase with thrombospond	11q24.3	170689	-1.94	-10.15	1.43E-22	1.49E-21	down Basal vs. Luminal
DNAH5	dynein axonemal heavy chain 5	5p15.2	1767	-1.63	-10.16	1.39E-22	1.45E-21	down Basal vs. Luminal
TUBA3E	tubulin alpha 3e	2q21.1	112714	-2.23	-10.16	1.32E-22	1.39E-21	down Basal vs. Luminal
FAM47E	family with sequence similarity 47 member	4q21.1	100129583	-1.07	-10.19	1.07E-22	1.13E-21	down Basal vs. Luminal
CCND1	cyclin D1	11q13.3	595	-1.26	-10.21	8.75E-23	9.36E-22	down Basal vs. Luminal
LDLRAD3	low density lipoprotein receptor class A don	11p13	143458	-1.15	-10.23	7.60E-23	8.20E-22	down Basal vs. Luminal
SLC7A8	solute carrier family 7 member 8	14q11.2	23428	-1.12	-10.23	7.34E-23	7.94E-22	down Basal vs. Luminal
PRLR	prolactin receptor	5p13.2	5618	-1.08	-10.23	7.18E-23	7.81E-22	down Basal vs. Luminal
HCAR1	hydroxycarboxylic acid receptor 1	12q24.31	27198	-1.57	-10.25	6.30E-23	6.91E-22	down Basal vs. Luminal
DEGS2	delta 4-desaturase, sphingolipid 2	14q32.2	123099	-1.52	-10.26	5.53E-23	6.18E-22	down Basal vs. Luminal
DACH1	dachshund family transcription factor 1	13q21.33	1602	-1.82	-10.26	5.38E-23	6.05E-22	down Basal vs. Luminal
KIF12	kinesin family member 12	9q32	113220	-1.72	-10.30	4.06E-23	4.69E-22	down Basal vs. Luminal
CDON	cell adhesion associated, oncogene regula	11q24.2	50937	-1.05	-10.32	3.31E-23	3.85E-22	down Basal vs. Luminal
PDZK1	PDZ domain containing 1	1q21.1	5174	-2.78	-10.34	2.85E-23	3.35E-22	down Basal vs. Luminal
ABCC8	ATP binding cassette subfamily C member	11p15.1	6833	-2.33	-10.43	1.22E-23	1.49E-22	down Basal vs. Luminal
ERICH3	glutamate rich 3	1p31.1	127254	-2.50	-10.43	1.22E-23	1.49E-22	down Basal vs. Luminal
PKIB	cAMP-dependent protein kinase inhibitor be	6q22.31	5570	-1.51	-10.46	9.08E-24	1.14E-22	down Basal vs. Luminal
FMO5	flavin containing monooxygenase 5	1q21.1	2330	-1.65	-10.47	8.46E-24	1.07E-22	down Basal vs. Luminal
RBM24	RNA binding motif protein 24	6p22.3	221662	-2.47	-10.49	7.40E-24	9.48E-23	down Basal vs. Luminal
FYB2	FYN binding protein 2	1p32.2	199920	-2.09	-10.49	7.36E-24	9.46E-23	down Basal vs. Luminal
ASB16	ankyrin repeat and SOCS box containing 1(	17q21.31	92591	-1.16	-10.50	6.77E-24	8.73E-23	down Basal vs. Luminal
PTPRT	protein tyrosine phosphatase receptor type	20q12-q13.11	11122	-2.60	-10.53	4.98E-24	6.66E-23	down Basal vs. Luminal
KCNK15	potassium two pore domain channel subfar	20q13.12	60598	-1.77	-10.55	3.97E-24	5.34E-23	down Basal vs. Luminal
ZNF92	zinc finger protein 92	7q11.21	168374	-1.11	-10.63	2.08E-24	2.92E-23	down Basal vs. Luminal
DNAH7	dynein axonemal heavy chain 7	2q32.3	56171	-1.31	-10.67	1.35E-24	1.94E-23	down Basal vs. Luminal
DRC3	dynein regulatory complex subunit 3	17p11.2	83450	-1.44	-10.69	1.21E-24	1.76E-23	down Basal vs. Luminal
HPN	hepsin	19q13.11	3249	-1.56	-10.70	1.10E-24	1.62E-23	down Basal vs. Luminal
DNAAF1	dynein axonemal assembly factor 1	16q24.1	123872	-1.65	-10.72	9.12E-25	1.35E-23	down Basal vs. Luminal
CFAP43	cilia and flagella associated protein 43	10q25.1	80217	-1.44	-10.74	7.34E-25	1.11E-23	down Basal vs. Luminal
LYPD6	LY6/PLAUR domain containing 6	2q23.2	130574	-1.91	-10.77	5.70E-25	8.91E-24	down Basal vs. Luminal
CFAP61	cilia and flagella associated protein 61	20p11.23	26074	-1.46	-10.77	5.45E-25	8.57E-24	down Basal vs. Luminal
PRR15	proline rich 15	7p14.3	222171	-1.55	-10.84	2.86E-25	4.67E-24	down Basal vs. Luminal
MAST4	microtubule associated serine/threonine kir	5q12.3	375449	-1.27	-10.86	2.34E-25	3.84E-24	down Basal vs. Luminal
ELP2	elongator acetyltransferase complex subun	18q12.2	55250	-1.08	-10.87	2.13E-25	3.53E-24	down Basal vs. Luminal
REPS2	RALBP1 associated Eps domain containing	Xp22.2	9185	-1.43	-10.88	2.09E-25	3.48E-24	down Basal vs. Luminal
DCDC1	doublecortin domain containing 1	11p13	341019	-1.47	-10.89	1.77E-25	3.00E-24	down Basal vs. Luminal
NEK10	NIMA related kinase 10	3p24.1	152110	-2.45	-10.89	1.76E-25	2.99E-24	down Basal vs. Luminal
STC2	stanniocalcin 2	5q35.2	8614	-2.22	-10.90	1.69E-25	2.92E-24	down Basal vs. Luminal
ADCY1	adenylate cyclase 1	7p12.3	107	-2.15	-10.96	9.30E-26	1.67E-24	down Basal vs. Luminal
SLC44A4	solute carrier family 44 member 4	6p21.33	80736	-1.83	-11.00	6.44E-26	1.19E-24	down Basal vs. Luminal
SUSD3	sushi domain containing 3	9q22.31	203328	-1.91	-11.00	6.43E-26	1.19E-24	down Basal vs. Luminal
TTC39A	tetratricopeptide repeat domain 39A	1p32.3	22996	-1.18	-11.02	5.52E-26	1.03E-24	down Basal vs. Luminal

Symbol	Description	Cytoband	Entrez Gene ID	Basal vs. Luminal (log2-ratio)	t	p-value[1]	q-value	Associated to
SLC27A2	solute carrier family 27 member 2	15q21.2	11001	-2.28	-11.04	4.33E-26	8.25E-25	down Basal vs. Luminal
AK8	adenylate kinase 8	9q34.13	158067	-1.04	-11.10	2.52E-26	4.98E-25	down Basal vs. Luminal
LRRC46	leucine rich repeat containing 46	17q21.32	90506	-1.36	-11.12	2.24E-26	4.47E-25	down Basal vs. Luminal
CELSR2	cadherin EGF LAG seven-pass G-type rece	1p13.3	1952	-1.21	-11.16	1.45E-26	2.94E-25	down Basal vs. Luminal
NEK5	NIMA related kinase 5	13q14.3	341676	-1.68	-11.20	9.78E-27	2.01E-25	down Basal vs. Luminal
SYT9	synaptotagmin 9	11p15.4	143425	-2.78	-11.22	8.59E-27	1.77E-25	down Basal vs. Luminal
SFXN2	sideroflexin 2	10q24.32	118980	-1.02	-11.24	6.91E-27	1.44E-25	down Basal vs. Luminal
UGCG	UDP-glucose ceramide glucosyltransferase	9q31.3	7357	-1.52	-11.27	5.42E-27	1.16E-25	down Basal vs. Luminal
KCND3	potassium voltage-gated channel subfamily	1p13.2	3752	-2.17	-11.44	1.03E-27	2.40E-26	down Basal vs. Luminal
NME5	NME/NM23 family member 5	5q31.2	8382	-1.75	-11.49	6.59E-28	1.57E-26	down Basal vs. Luminal
FAM161B	FAM161 centrosomal protein B	14q24.3	145483	-1.10	-11.50	5.64E-28	1.36E-26	down Basal vs. Luminal
ERBB4	erb-b2 receptor tyrosine kinase 4	2q34	2066	-2.04	-11.52	4.88E-28	1.21E-26	down Basal vs. Luminal
ACOT4	acyl-CoA thioesterase 4	14q24.3	122970	-1.07	-11.57	2.82E-28	7.21E-27	down Basal vs. Luminal
ANXA9	annexin A9	1q21.3	8416	-1.49	-11.58	2.75E-28	7.11E-27	down Basal vs. Luminal
KAT6B	lysine acetyltransferase 6B	10q22.2	23522	-1.04	-11.61	1.98E-28	5.16E-27	down Basal vs. Luminal
SERPINA11	serpin family A member 11	14q32.13	256394	-2.75	-11.66	1.18E-28	3.15E-27	down Basal vs. Luminal
NRIP1	nuclear receptor interacting protein 1	21q11.2-q21.1	8204	-1.07	-11.67	1.09E-28	2.95E-27	down Basal vs. Luminal
ELOVL5	ELOVL fatty acid elongase 5	6p12.1	60481	-1.14	-11.68	9.88E-29	2.73E-27	down Basal vs. Luminal
CCDC40	coiled-coil domain containing 40	17q25.3	55036	-1.16	-11.71	7.83E-29	2.25E-27	down Basal vs. Luminal
TSPAN13	tetraspanin 13	7p21.1	27075	-1.01	-11.73	6.02E-29	1.81E-27	down Basal vs. Luminal
RALGPS2	Ral GEF with PH domain and SH3 binding	1q25.2	55103	-1.15	-11.82	2.69E-29	8.40E-28	down Basal vs. Luminal
NXNL2	nucleoredoxin like 2	9q22.1	158046	-2.06	-11.92	9.49E-30	3.06E-28	down Basal vs. Luminal
CFAP57	cilia and flagella associated protein 57	1p34.2	149465	-1.74	-11.93	8.38E-30	2.73E-28	down Basal vs. Luminal
BBOF1	basal body orientation factor 1	14q24.3	80127	-1.01	-11.98	5.26E-30	1.76E-28	down Basal vs. Luminal
INPP4B	inositol polyphosphate-4-phosphatase type	4q31.21	8821	-1.40	-11.99	4.95E-30	1.70E-28	down Basal vs. Luminal
SYTL4	synaptotagmin like 4	Xq22.1	94121	-1.07	-12.00	4.60E-30	1.60E-28	down Basal vs. Luminal
EFCAB6	EF-hand calcium binding domain 6	22q13.2-q13.31	64800	-1.25	-12.01	4.13E-30	1.46E-28	down Basal vs. Luminal
MCCC2	methylcrotonoyl-CoA carboxylase 2	5q13.2	64087	-1.02	-12.02	3.70E-30	1.33E-28	down Basal vs. Luminal
ITIH6	inter-alpha-trypsin inhibitor heavy chain fam	Xp11.22	347365	-1.96	-12.04	2.85E-30	1.05E-28	down Basal vs. Luminal
ANKS1B	ankyrin repeat and sterile alpha motif doma	12q23.1	56899	-1.25	-12.09	1.88E-30	7.12E-29	down Basal vs. Luminal
SALL2	spalt like transcription factor 2	14q11.2	6297	-1.10	-12.11	1.51E-30	5.85E-29	down Basal vs. Luminal
NKAIN1	sodium/potassium transporting ATPase inte	1p35.2	79570	-2.82	-12.14	1.09E-30	4.25E-29	down Basal vs. Luminal
NAT1	N-acetyltransferase 1	8p22	9	-2.52	-12.15	9.92E-31	3.96E-29	down Basal vs. Luminal
CASC1	cancer susceptibility 1	12p12.1	55259	-1.48	-12.18	7.58E-31	3.06E-29	down Basal vs. Luminal
DNALI1	dynein axonemal light intermediate chain 1	1p34.3	7802	-1.73	-12.22	4.99E-31	2.06E-29	down Basal vs. Luminal
LMX1B	LIM homeobox transcription factor 1 beta	9q33.3	4010	-1.18	-12.24	4.05E-31	1.69E-29	down Basal vs. Luminal
TMEM26	transmembrane protein 26	10q21.2	219623	-2.21	-12.25	3.82E-31	1.61E-29	down Basal vs. Luminal
GFRA1	GDNF family receptor alpha 1	10q25.3	2674	-2.82	-12.32	1.74E-31	7.63E-30	down Basal vs. Luminal
CMBL	carboxymethylenebutenolidase homolog	5p15.2	134147	-1.20	-12.34	1.52E-31	6.73E-30	down Basal vs. Luminal
ZMYND10	zinc finger MYND-type containing 10	3p21.31	51364	-1.76	-12.37	1.05E-31	4.73E-30	down Basal vs. Luminal
IL6ST	interleukin 6 signal transducer	5q11.2	3572	-2.30	-12.40	7.81E-32	3.59E-30	down Basal vs. Luminal
PREX1	phosphatidylinositol-3,4,5-trisphosphate de	20q13.13	57580	-1.38	-12.62	9.08E-33	4.39E-31	down Basal vs. Luminal
SYBU	syntabulin	8q23.2	55638	-1.40	-12.70	3.94E-33	1.96E-31	down Basal vs. Luminal
LONRF2	LON peptidase N-terminal domain and ring	2q11.2	164832	-1.95	-12.82	1.19E-33	6.18E-32	down Basal vs. Luminal
CACNA1D	calcium voltage-gated channel subunit alph	3p21.1	776	-1.85	-12.88	6.40E-34	3.41E-32	down Basal vs. Luminal
CLSTN2	calsyntenin 2	3q23	64084	-2.15	-12.99	2.04E-34	1.10E-32	down Basal vs. Luminal
NPY1R	neuropeptide Y receptor Y1	4q32.2	4886	-3.72	-13.03	1.34E-34	7.36E-33	down Basal vs. Luminal
KCNJ11	potassium voltage-gated channel subfamily	11p15.1	3767	-1.41	-13.06	9.75E-35	5.60E-33	down Basal vs. Luminal
UBXN10	UBX domain protein 10	1p36.12	127733	-1.71	-13.17	2.98E-35	1.79E-33	down Basal vs. Luminal
SPEF2	sperm flagellar 2	5p13.2	79925	-1.12	-13.19	2.39E-35	1.46E-33	down Basal vs. Luminal
AR	androgen receptor	Xq12	367	-2.30	-13.24	1.41E-35	8.96E-34	down Basal vs. Luminal
AGR2	anterior gradient 2, protein disulphide isome	7p21.1	10551	-2.61	-13.31	7.24E-36	4.67E-34	down Basal vs. Luminal
SLC16A6	solute carrier family 16 member 6	17q24.2	9120	-2.18	-13.32	6.50E-36	4.33E-34	down Basal vs. Luminal
ACADSB	acyl-CoA dehydrogenase short/branched c	10q26.13	36	-1.35	-13.33	5.54E-36	3.77E-34	down Basal vs. Luminal
MAGED2	MAGE family member D2	Xp11.21	10916	-1.16	-13.45	1.56E-36	1.11E-34	down Basal vs. Luminal
SIAH2	siah E3 ubiquitin protein ligase 2	3q25.1	6478	-1.20	-13.48	1.16E-36	8.67E-35	down Basal vs. Luminal
MLPH	melanophilin	2q37.3	79083	-1.60	-13.51	8.81E-37	6.75E-35	down Basal vs. Luminal
SLC19A2	solute carrier family 19 member 2	1q24.2	10560	-1.20	-13.54	6.15E-37	4.81E-35	down Basal vs. Luminal
ZNF396	zinc finger protein 396	18q12.2	252884	-1.04	-13.67	1.63E-37	1.30E-35	down Basal vs. Luminal
RGS22	regulator of G protein signaling 22	8q22.2	26166	-2.85	-13.72	9.31E-38	7.61E-36	down Basal vs. Luminal
TPRG1	tumor protein p63 regulated 1	3q28	285386	-2.96	-13.72	8.77E-38	7.32E-36	down Basal vs. Luminal
BCL2	BCL2 apoptosis regulator	18q21.33	596	-1.41	-13.74	7.52E-38	6.43E-36	down Basal vs. Luminal
ZNF587	zinc finger protein 587	19q13.43	84914	-1.17	-13.78	4.82E-38	4.22E-36	down Basal vs. Luminal
DNAJC12	DnaJ heat shock protein family (Hsp40) me	10q21.3	56521	-2.29	-13.87	1.82E-38	1.67E-36	down Basal vs. Luminal
SCUBE2	signal peptide, CUB domain and EGF like c	11p15.4	57758	-2.84	-13.88	1.72E-38	1.63E-36	down Basal vs. Luminal
ABAT	4-aminobutyrate aminotransferase	16p13.2	18	-1.84	-13.92	1.13E-38	1.13E-36	down Basal vs. Luminal
FSIP1	fibrous sheath interacting protein 1	15q14	161835	-2.60	-14.15	9.40E-40	9.60E-38	down Basal vs. Luminal
PARD6B	par-6 family cell polarity regulator beta	20q13.13	84612	-1.77	-14.18	6.23E-40	6.54E-38	down Basal vs. Luminal
GATA3	GATA binding protein 3	10p14	2625	-1.50	-14.20	5.17E-40	5.76E-38	down Basal vs. Luminal
IGF1R	insulin like growth factor 1 receptor	15q26.3	3480	-1.63	-14.54	1.24E-41	1.52E-39	down Basal vs. Luminal
SAMD15	sterile alpha motif domain containing 15	14q24.3	161394	-1.56	-14.72	1.77E-42	2.24E-40	down Basal vs. Luminal
RERG	RAS like estrogen regulated growth inhibito	12p12.3	85004	-1.55	-14.92	1.84E-43	2.41E-41	down Basal vs. Luminal
AFF3	AF4/FMR2 family member 3	2q11.2	3899	-2.42	-15.04	5.18E-44	7.62E-42	down Basal vs. Luminal
CA12	carbonic anhydrase 12	15q22.2	771	-1.84	-15.05	4.45E-44	6.82E-42	down Basal vs. Luminal
RABEP1	rabaptin, RAB GTPase binding effector pro	17p13.2	9135	-1.08	-15.22	6.28E-45	1.00E-42	down Basal vs. Luminal
GREB1	growth regulating estrogen receptor binding	2p25.1	9687	-2.19	-15.28	3.30E-45	5.52E-43	down Basal vs. Luminal
PGR	progesterone receptor	11q22.1	5241	-3.57	-15.30	2.61E-45	4.57E-43	down Basal vs. Luminal
CCDC170	coiled-coil domain containing 170	6q25.1	80129	-1.81	-15.30	2.58E-45	4.57E-43	down Basal vs. Luminal
ARSG	arylsulfatase G	17q24.2	22901	-1.47	-15.41	7.96E-46	1.54E-43	down Basal vs. Luminal
SLC39A6	solute carrier family 39 member 6	18q12.2	25800	-2.07	-15.45	4.65E-46	9.50E-44	down Basal vs. Luminal
NUDT12	nudix hydrolase 12	5q21.2	83594	-1.07	-15.47	3.93E-46	8.49E-44	down Basal vs. Luminal
FOXA1	forkhead box A1	14q21.1	3169	-2.17	-15.48	3.48E-46	8.00E-44	down Basal vs. Luminal
MINDY1	MINDY lysine 48 deubiquitinase 1	1q21.3	55793	-1.02	-15.48	3.37E-46	8.00E-44	down Basal vs. Luminal
CELSR1	cadherin EGF LAG seven-pass G-type rece	22q13.31	9620	-1.43	-15.57	1.27E-46	3.34E-44	down Basal vs. Luminal
FAM214A	family with sequence similarity 214 membe	15q21.2-q21.3	56204	-1.27	-15.62	7.07E-47	2.00E-44	down Basal vs. Luminal
MAPT	microtubule associated protein tau	17q21.31	4137	-2.45	-15.76	1.52E-47	4.64E-45	down Basal vs. Luminal
AGR3	anterior gradient 3, protein disulphide isome	7p21.1	155465	-3.23	-15.79	1.08E-47	3.60E-45	down Basal vs. Luminal
ZNF552	zinc finger protein 552	19q13.43	79818	-1.48	-16.06	4.75E-49	1.94E-46	down Basal vs. Luminal
XBP1	X-box binding protein 1	22q12.1|22q12	7494	-1.37	-16.19	9.83E-50	4.52E-47	down Basal vs. Luminal
C5AR2	complement component 5a receptor 2	19q13.32	27202	-1.81	-16.87	3.64E-53	1.91E-50	down Basal vs. Luminal
KDM4B	lysine demethylase 4B	19p13.3	23030	-1.28	-17.60	6.39E-57	3.91E-54	down Basal vs. Luminal
CT62	cancer/testis antigen 62	15q23	196993	-2.25	-17.66	3.25E-57	2.39E-54	down Basal vs. Luminal
TBC1D9	TBC1 domain family member 9	4q31.21	23158	-1.87	-17.77	8.95E-58	8.22E-55	down Basal vs. Luminal
ESR1	estrogen receptor 1	6q25.1-q25.2	2099	-2.46	-17.80	5.83E-58	7.14E-55	down Basal vs. Luminal
MYB	MYB proto-oncogene, transcription factor	6q23.3	4602	-1.70	-18.28	2.03E-60	3.73E-57	down Basal vs. Luminal
THSD4	thrombospondin type 1 domain containing 4	15q23	79875	-2.19	-19.23	1.89E-65	6.95E-62	down Basal vs. Luminal

[1], moderated t-test

Supplementary Table 6: Gene ontologies of the 906 genes differentially expressed between the ER+/HER2- Luminal and Basal breast cancers (TCGA)

GO:BP	Terms	up Basal vs. Luminal				down Basal vs. Luminal			
Terms ID		N	Genes	p-value	q-value	N	Genes	p-value	q-value
GO:0006955	immune response	48	CXCL5,NFIL3,IL1R2,CCL18,CCL13,	1.62E-22	1.06E-19				
GO:0006954	inflammatory response	42	CHST2,S100A8,TNIP3,CXCL5,GAL,	2.79E-17	9.14E-15				
GO:0030593	neutrophil chemotaxis	15	S100A8,S100A9,CCL18,CCL13,CX	1.48E-11	3.12E-09				
GO:0002690	positive regulation of leukocyte chemotaxis	9	CXCL5,CXCL1,CXCL3,IL6,CXCL10,	1.90E-11	3.12E-09				
GO:0006935	chemotaxis	19	CXCL5,CCL18,CCL13,FOSL1,CXC	2.79E-11	3.65E-09				
GO:0031124	mRNA 3-end processing	346	PSAT1,MTHFD1L,UPP1,CHST2,GL	1.75E-08	1.91E-06				
GO:0071347	cellular response to interleukin-1	12	CCL18,CCL13,CHI3L1,CCL7,IL6,C	2.28E-08	2.14E-06				
GO:0007267	cell-cell signaling	22	CXCL5,ADM,S100A9,CCL18,CCL1	6.15E-08	4.48E-06				
GO:0071346	cellular response to interferon-gamma	10	CCL18,CCL13,ASS1,CCL7,CX3CL1	7.87E-08	4.73E-06				
GO:0002548	monocyte chemotaxis	9	CCL18,CCL13,CCL7,IL6,CX3CL1,C	7.93E-08	4.73E-06				
GO:0071356	cellular response to tumor necrosis factor	13	CCL18,CCL13,CHI3L1,ASS1,CCL7,	1.84E-07	9.63E-06				
GO:0002009	morphogenesis of an epithelium	6	KRT16,MATK,KRT6A,CA9,RIPK4,S	1.91E-07	9.63E-06				
GO:0030855	epithelial cell differentiation	11	DLX5,RHCG,CRHR1,ALDOC,BARX	5.52E-07	2.58E-05				
GO:0071222	cellular response to lipopolysaccharide	13	TNIP3,GSTP1,OPRK1,ASS1,IFNG,I	7.90E-07	3.45E-05				
GO:0002250	adaptive immune response	13	JAK3,LILRA3,CD7,LAMP3,CTLA4,I	2.25E-06	9.23E-05				
GO:0008544	epidermis development	11	FABP5,KRT83,KRT16,CASP14,SC	3.08E-06	1.19E-04				
GO:0007165	signal transduction	53	CRABP1,CXCL5,ADM,S100A9,SH2	3.62E-06	1.32E-04				
GO:0030574	collagen catabolic process	10	COL11A2,COL4A4,COL26A1,COL4	4.58E-06	1.58E-04				
GO:0002544	chronic inflammatory response	4	S100A8,S100A9,CCL5,CXCL13	6.62E-06	2.07E-04				
GO:0010820	positive regulation of T cell chemotaxis	4	CCL5,XCL2,S100A7,CXCL13	6.62E-06	2.07E-04				
GO:0030101	natural killer cell activation	4	ULBP2,ULBP1,IL21R,ULBP3	1.11E-05	3.31E-04				
GO:0019835	cytolysis	5	GZMB,PRF1,GZMM,LYZ,GZMA	1.26E-05	3.60E-04				
GO:0007166	cell surface receptor signaling pathway	20	IL12RB2,MARCO,LAG3,CRHR1,DO	2.39E-05	6.54E-04				
GO:0016266	O-glycan processing	8	B3GNT7,B3GNT5,GALNT12,B3GN	2.98E-05	7.82E-04				
GO:0090026	positive regulation of monocyte chemotaxis	4	PLA2G7,CCL5,CXCL10,S100A7	3.92E-05	9.90E-04				
GO:0030154	cell differentiation	24	CSRP2,FOXC1,CBX2,NXN,PIWIL4,	7.81E-05	1.90E-03				
GO:0006959	humoral immune response	6	PDCD1,IFNG,IL6,LTA,POU2AF1,SH	1.05E-04	2.44E-03				
GO:2000406	positive regulation of T cell migration	3	CCL5,CCL20,CXCL10	1.09E-04	2.44E-03				
GO:0007568	aging	12	ADM,ASS1,KRT83,KRT16,IL6,CX3C	1.12E-04	2.44E-03				
GO:0008284	positive regulation of cell proliferation	25	CXCL5,ADM,ST8SIA1,IL12RB2,FO	1.29E-04	2.72E-03				
GO:0030326	embryonic limb morphogenesis	6	DLX5,PRRX2,DKK1,GDF5,BMP7,H	1.45E-04	2.97E-03				
GO:0006968	cellular defense response	7	FOSL1,PRF1,GNLY,CD19,LBP,SH2	1.51E-04	2.99E-03				
GO:0006569	tryptophan catabolic process	3	IDO1,KYNU,TDO2	1.68E-04	3.15E-03				
GO:0007204	positive regulation of cytosolic calcium ion concentration	10	CIB2,ADM,JAK3,CRHR1,CD38,S1P	1.80E-04	3.28E-03				
GO:0030217	T cell differentiation	5	BCL11A,LCK,TBX21,CD3D,ZAP70	1.85E-04	3.28E-03				
GO:0001878	response to yeast	3	ADM,PTX3,IL6	2.47E-04	4.26E-03				
GO:0006493	protein O-linked glycosylation	8	B3GNT7,B3GNT5,GALNT12,B3GN	2.83E-04	4.76E-03				
GO:0030198	extracellular matrix organization	19	MATN4,COL11A2,COL4A4,BMP2,E	4.25E-04	6.97E-03				
GO:0009409	response to cold	5	TRPM8,ADM,IL6,CXCL10,PCSK1N	5.07E-04	7.91E-03				
GO:0010043	response to zinc ion	5	S100A8,SLC30A3,S100A9,ASS1,LC	5.07E-04	7.91E-03				
GO:0009615	response to virus	9	BATF3,FOSL1,CDK6,IFNG,CCL5,IL	5.55E-04	8.47E-03				
GO:0030322	stabilization of membrane potential	3	KCNN4,KCNK5,KCNK1	6.37E-04	9.49E-03				
GO:0015992	proton transport	6	SLC15A1,SLC2A5,SLC2A6,SLC15A	7.13E-04	1.04E-02				
GO:0001503	ossification	7	FOXC1,RUNX3,COL11A2,MMP9,C	7.84E-04	1.12E-02				
GO:0008283	cell proliferation	20	FSCN1,FOXC1,DLX5,SH2D2A,IL2R	8.32E-04	1.16E-02				
GO:0006952	defense response	6	KCNN4,LILRA3,IL32,CX3CL1,PTPR	8.90E-04	1.22E-02				
GO:0018146	keratan sulfate biosynthetic process	4	CHST2,B3GNT7,B3GNT4,B3GNT3	9.52E-04	1.27E-02				
GO:0007399	nervous system development	16	FZD9,DLX5,APBA2,GAL,GFRA3,SC	9.97E-04	1.31E-02				
GO:0019722	calcium-mediated signaling	5	SPHK1,MCTP2,CXCR3,CXCL8,ED	1.02E-03	1.31E-02				
GO:0071294	cellular response to zinc ion	3	MT1X,MT1M,MT1G	1.06E-03	1.34E-02				
GO:0007154	cell communication	4	CCL18,GJB3,GJB5,KREMEN2	1.12E-03	1.39E-02				
GO:0001501	skeletal system development	10	HAPLN3,FOXC1,EN1,DLX5,COL11	1.18E-03	1.43E-02				
GO:0030216	keratinocyte differentiation	6	CSTA,TGM1,IVL,KRT16,SCEL,S10	1.49E-03	1.71E-02				
GO:0007600	sensory perception	2	PNOC,OPRK1	1.59E-03	1.77E-02				
GO:0022617	extracellular matrix disassembly	9	COL11A2,COL4A4,COL26A1,COL4	1.71E-03	1.87E-02				
GO:1902476	chloride transmembrane transport	7	SLC26A9,CLCN4,GABRE,CLIC3,TT	1.86E-03	1.97E-02				
GO:0001771	immunological synapse formation	2	PRF1,CD6	2.23E-03	2.15E-02				
GO:0003376	sphingosine-1-phosphate signaling pathway	2	SPHK1,S1PR4	2.23E-03	2.15E-02				
GO:0010759	positive regulation of macrophage chemotaxis	2	CX3CL1,CCL5	2.23E-03	2.15E-02				
GO:0015793	glycerol transport	2	AQP9,AQP5	2.23E-03	2.15E-02				
GO:0021527	spinal cord association neuron differentiation	2	LMO4,PAX7	2.23E-03	2.15E-02				
GO:0030183	B cell differentiation	5	FZD9,JAK3,BCL11A,CD79A,CR2	2.30E-03	2.18E-02				
GO:0006953	acute-phase response	4	ASS1,IL6,LBP,SAA1	2.35E-03	2.18E-02				
GO:0030509	BMP signaling pathway	6	DLX5,RGMA,BMP2,NKX2-5,GDF5,B	2.36E-03	2.18E-02				
GO:0098655	cation transmembrane transport	6	SLC30A3,HTR3A,FAM26F,SLC7A5,	2.57E-03	2.31E-02				
GO:0009888	tissue development	2	BARX1,WT1	3.00E-03	2.46E-02				
GO:0009992	cellular water homeostasis	2	AQP9,AQP5	3.00E-03	2.46E-02				
GO:0010038	response to metal ion	2	NDRG1,MT1X	3.00E-03	2.46E-02				
GO:0010996	response to auditory stimulus	2	IL6,CXCL10	3.00E-03	2.46E-02				
GO:0071361	cellular response to ethanol	2	CCL7,CCL5	3.00E-03	2.46E-02				
GO:1900745	positive regulation of p38MAPK cascade	2	OPRK1,BMP2	3.00E-03	2.46E-02				
GO:0009611	response to wounding	5	ADM,ACHE,HOXB13,KLK6,KLK8	3.12E-03	2.53E-02				
GO:0007588	excretion	4	KCNK5,AQP9,ATP6V0A4,AQP5	3.44E-03	2.75E-02				
GO:0006874	cellular calcium ion homeostasis	7	TRPM8,CCL13,CCL7,CCL5,CCL8,	3.61E-03	2.84E-02				
GO:0016337	single organismal cell-cell adhesion	7	CSTA,ICAM5,PKP1,ROPN1B,CDH3	3.87E-03	2.92E-02				
GO:0002446	neutrophil mediated immunity	2	CXCL5,IL6	3.92E-03	2.92E-02				
GO:0014032	neural crest cell development	2	FOXC1,SOX11	3.92E-03	2.92E-02				
GO:0072488	ammonium transmembrane transport	2	RHCG,SLC22A3	3.92E-03	2.92E-02				
GO:0007422	peripheral nervous system development	3	GFRA3,SOX8,UGT8	4.07E-03	2.96E-02				
GO:0010800	positive regulation of peptidyl-threonine phosphorylation	3	SPHK1,CHI3L1,BMP7	4.07E-03	2.96E-02				
GO:0002062	chondrocyte differentiation	4	RUNX3,WNT10B,BMP2,GDF5	4.34E-03	3.13E-02				
GO:0016525	negative regulation of angiogenesis	5	FOXC1,COL4A3,CXCL10,SPINK5,C	4.95E-03	3.52E-02				
GO:2000678	negative regulation of transcription regulatory region DNA binding	2	SOX11,LHX2	4.99E-03	3.52E-02				
GO:0001666	response to hypoxia	10	ADM,CRHR1,LCT,BMP2,CX3CL1,C	5.28E-03	3.68E-02				
GO:0030178	negative regulation of Wnt signaling pathway	4	NXN,BARX1,TRABD2A,DKK1	5.40E-03	3.73E-02				
GO:0009954	proximal/distal pattern formation	3	EN1,HOXC11,HOXC10	5.47E-03	3.74E-02				
GO:0007565	female pregnancy	6	ADM,PNOC,IDO1,FOSL1,CRHR1,C	5.61E-03	3.79E-02				
GO:0008015	blood circulation	4	ADM,XCL2,COL4A3,CXCL10	5.99E-03	3.97E-02				
GO:0001843	neural tube closure	6	MTHFD1L,ADM,LMO4,RGMA,LHX2	6.01E-03	3.97E-02				
GO:0001516	prostaglandin biosynthetic process	2	PTGDS,EDN2	6.23E-03	3.97E-02				
GO:0001817	regulation of cytokine production	2	ACE2,UBASH3A	6.23E-03	3.97E-02				
GO:0071276	cellular response to cadmium ion	2	MT1X,MT1G	6.23E-03	3.97E-02				
GO:0019731	antibacterial humoral response	3	ADM,DEFB1,SLPI	6.29E-03	3.97E-02				
GO:0006821	chloride transport	4	SLC26A9,CLCN4,CLIC3,TTYH1	6.63E-03	4.06E-02				
GO:0071320	cellular response to cAMP	4	WNT10B,ASS1,AQP9,WT1	6.63E-03	4.06E-02				
GO:0021510	spinal cord development	3	SOX11,PHGDH,ZIC1	7.18E-03	4.36E-02				
GO:0006810	transport	17	SLC15A1,CRABP1,SLC30A3,CLCN	7.46E-03	4.43E-02				
GO:0010460	positive regulation of heart rate	2	ADM,EDN2	7.62E-03	4.43E-02				
GO:0010975	regulation of neuron projection development	2	CHODL,KLK6	7.62E-03	4.43E-02				
GO:0022409	positive regulation of cell-cell adhesion	2	L1CAM,SIRPG	7.62E-03	4.43E-02				
GO:0006486	protein glycosylation	6	B3GNT5,ST8SIA1,B3GAT1,FUT3,B	7.87E-03	4.53E-02				
GO:1904659	glucose transmembrane transport	2	SLC2A5,SLC2A6	9.19E-03	5.15E-02				
GO:1901215	negative regulation of neuron death	3	CRHR1,IL6,CCL5	9.19E-03	5.15E-02				
GO:0007157	heterophilic cell-cell adhesion via plasma membrane cell adhesio	4	L1CAM,NCR3,CD6,TIGIT	9.64E-03	5.36E-02				
GO:0030513	positive regulation of BMP signaling pathway	3	SOX11,FOXD1,GDF5	1.03E-02	5.69E-02				
GO:0030182	neuron differentiation	6	ITM2C,WNT6,RUNX3,WNT10B,SO	1.08E-02	5.88E-02				
GO:0006885	regulation of pH	2	RHCG,ATP6V0A4	1.09E-02	5.93E-02				
GO:0002026	regulation of the force of heart contraction	2	ADM,IFNG	1.29E-02	6.44E-02				
GO:0002526	acute inflammatory response	2	S100A8,DEFB1	1.29E-02	6.44E-02				
GO:0007130	synaptonemal complex assembly	2	HORMAD1,SYCE1L	1.29E-02	6.44E-02				
GO:0010579	positive regulation of adenylate cyclase activity involved in G-prot	2	CRHR1,CHRM1	1.29E-02	6.44E-02				
GO:0017015	regulation of transforming growth factor beta receptor signaling p	2	SOX11,FOLR1	1.29E-02	6.44E-02				
GO:0018149	peptide cross-linking	3	CSTA,TGM1,IVL	1.29E-02	6.44E-02				
GO:0030501	positive regulation of bone mineralization	3	WNT10B,BMP2,BMP7	1.29E-02	6.44E-02				
GO:0030307	positive regulation of cell growth	5	S100A8,S100A9,SPHK1,CD38,KRT	1.32E-02	6.58E-02				
GO:0022008	neurogenesis	3	FABP7,CHAC1,BCL11B	1.43E-02	7.03E-02				
GO:0007214	gamma-aminobutyric acid signaling pathway	2	GABBR2,GABRE	1.50E-02	7.27E-02				
GO:0007584	response to nutrient	5	LCT,ASS1,LTA,LIPG,CD3E	1.51E-02	7.27E-02				
GO:0007155	cell adhesion	19	HAPLN3,CD72,MSLN,IL32,ACHE,L	1.82E-02	8.63E-02				
GO:0009790	embryo development	3	NPM2,BMP2,SCEL	1.90E-02	8.92E-02				
GO:0002376	immune system process	2	KCNN4,IDO1	1.97E-02	9.04E-02				
GO:0090023	positive regulation of neutrophil chemotaxis	2	CXCL8,LBP	1.97E-02	9.04E-02				
GO:0016042	lipid catabolic process	5	PLA2G7,PLCH1,PLA2G2D,PLA2G2	2.04E-02	9.29E-02				
GO:0010811	positive regulation of cell-substrate adhesion	3	EGFL6,COL26A1,SPOCK2	2.08E-02	9.35E-02				
GO:0097191	extrinsic apoptotic signaling pathway	4	IFNG,IL12A,CD70,CD27	2.10E-02	9.38E-02				
GO:0008037	cell recognition	2	NCR3,TIGIT	2.23E-02	9.77E-02				
GO:0014911	positive regulation of smooth muscle cell migration	2	P2RY6,CCL5	2.23E-02	9.77E-02				
GO:0071549	cellular response to dexamethasone stimulus	2	ASS1,IL6	2.23E-02	9.77E-02				

GO:BP Terms ID	Terms	up Basal vs. Luminal N	Genes	p-value	q-value	down Basal vs. Luminal N	Genes	p-value	q-value
GO:0097192	extrinsic apoptotic signaling pathway in absence of ligand	3	BCL2A1,CASP14,LCN2	2.27E-02	9.79E-02				
GO:0019221	cytokine-mediated signaling pathway	12	IL22RA2,IL1R2,IL12RB2,MID1,SOC	2.28E-02	9.79E-02				
GO:0001942	hair follicle development	3	DKK1,WNT10A,LHX2	2.47E-02	9.99E-02				
GO:0010862	positive regulation of pathway-restricted SMAD protein phosphory	3	BMP2,GDF5,BMP7	2.47E-02	9.99E-02				
GO:0007126	meiotic nuclear division	2	HORMAD1,SMC1B	2.52E-02	1.01E-01				
GO:0008652	cellular amino acid biosynthetic process	2	PSAT1,PHGDH	2.52E-02	1.01E-01				
GO:0030325	adrenal gland development	2	CRHR1,WT1	2.82E-02	1.04E-01				
GO:0001658	branching involved in ureteric bud morphogenesis	3	WNT6,BMP2,WT1	2.90E-02	1.04E-01				
GO:0006919	activation of cysteine-type endopeptidase activity involved in apop	5	S100A8,S100A9,CASP14,LCK,COL	2.98E-02	1.04E-01				
GO:0008285	negative regulation of cell proliferation	16	RARRES1,ADM,NDRG1,FOSL1,CD	3.02E-02	1.04E-01				
GO:0001816	cytokine production	2	S100A8,S100A9	3.14E-02	1.06E-01				
GO:0006656	phosphatidylcholine biosynthetic process	2	FABP5,ACHE	3.14E-02	1.06E-01				
GO:0019216	regulation of lipid metabolic process	2	FGFR4,BCL11B	3.14E-02	1.06E-01				
GO:2001243	negative regulation of intrinsic apoptotic signaling pathway	2	BCL2A1,MMP9	3.14E-02	1.06E-01				
GO:0007219	Notch signaling pathway	8	FOXC1,GZMB,CDK6,IL2RA,CHAC1,	3.31E-02	1.08E-01				
GO:0007389	pattern specification process	2	MID1,ZIC1	3.48E-02	1.08E-01				
GO:0019433	triglyceride catabolic process	2	FABP7,FABP5	3.48E-02	1.08E-01				
GO:0098656	anion transmembrane transport	3	SLCO5A1,SLCO4A1,SLC4A11	3.61E-02	1.08E-01				
GO:0006879	cellular iron ion homeostasis	4	ATP6V1C2,LCN2,ATP6V0A4,CP	3.67E-02	1.09E-01				
GO:0006366	transcription from RNA polymerase II promoter	21	BATF3,FOXC1,DLX5,NFIL3,LMO4,R	3.69E-02	1.10E-01				
GO:0001707	mesoderm formation	2	DKK1,BMP7	3.83E-02	1.11E-01				
GO:0007159	leukocyte cell-cell adhesion	2	L1CAM,CCL5	3.83E-02	1.11E-01				
GO:0016055	Wnt signaling pathway	8	NXN,WNT6,WNT10B,CCNE1,TRAB	4.05E-02	1.11E-01				
GO:0071230	cellular response to amino acid stimulus	3	SOCS1,ASS1,CCL5	4.14E-02	1.11E-01				
GO:0003333	amino acid transmembrane transport	2	SLC7A5,SLC6A14	4.21E-02	1.11E-01				
GO:0006096	glycolytic process	2	ALDOC,HK3	4.21E-02	1.11E-01				
GO:0006654	phosphatidic acid biosynthetic process	2	PLA2G2D,PLA2G2A	4.21E-02	1.11E-01				
GO:0007271	synaptic transmission, cholinergic	2	HTR3A,CHRM1	4.21E-02	1.11E-01				
GO:0007274	neuromuscular synaptic transmission	2	HTR3A,CHRM1	4.21E-02	1.11E-01				
GO:0010634	positive regulation of epithelial cell migration	2	GLIPR2,IFNG	4.21E-02	1.11E-01				
GO:1901687	glutathione derivative biosynthetic process	2	GSTP1,GSTA1	4.21E-02	1.11E-01				
GO:0006749	glutathione metabolic process	3	GSTP1,CLIC3,GSTA1	4.42E-02	1.15E-01				
GO:0030203	glycosaminoglycan metabolic process	6	CHST2,B3GNT7,FOXC1,B3GNT4,B	4.54E-02	1.16E-01				
GO:0006730	one-carbon metabolic process	2	MTHFD1L,CA9	4.60E-02	1.16E-01				
GO:0006958	complement activation, classical pathway	2	CR1,CR2	4.60E-02	1.16E-01				
GO:0007186	G-protein coupled receptor signaling pathway	32	FZD9,GABBR2,CXCL5,CCL18,CCL	3.26E-08	2.67E-06	16	CARTPT,ATRNL1,PYY,NPY5R,TME	1.68E-02	1.11E-01
GO:0010628	positive regulation of gene expression	16	SLC26A9,WNT6,NFIL3,TLR9,CDK6	1.61E-04	3.10E-03	9	PIP,MYT1,AZU1,SLC6A4,NOS1AP,	3.99E-02	1.23E-01
GO:0006811	ion transport	11	SLC26A9,SLC15A1,SLC7A5,SLCO	1.52E-03	1.71E-02	7	SLC1A1,SLC7A2,TMC3,WNK4,SLC	3.49E-02	1.21E-01
GO:2001244	positive regulation of intrinsic apoptotic signaling pathway	4	S100A8,PLEKHF1,S100A9,LCK	1.78E-03	1.91E-02	2	PMAIP1,BCL2	4.02E-02	1.23E-01
GO:0007169	transmembrane receptor protein tyrosine kinase signaling pathwa	8	JAK3,CD7,DOK2,CD8B,MATK,LCK,	1.33E-03	1.57E-02	6	FLT3,RET,SRMS,PTPRT,ERBB4,IG	7.67E-03	7.67E-02
GO:0019229	regulation of vasoconstriction	3	ACE2,PRKCQ,EDN2	2.45E-03	2.23E-02	2	ATP1A2,AGTR1	1.30E-02	9.79E-02
GO:0007417	central nervous system development	8	HAPLN3,B3GNT5,GSTP1,DNER,MA	3.64E-03	2.84E-02	6	ROBO2,DCLK1,CHST8,RPS6KA6,A	1.64E-02	1.11E-01
GO:0071773	cellular response to BMP stimulus	3	DLX5,BMP2,BMP7	6.29E-03	3.97E-02	2	BMPR1B,GATA3	2.58E-02	1.11E-01
GO:0006865	amino acid transport	3	SLC7A5,SLC16A10,SLC6A14	1.15E-02	6.11E-02	2	SLC7A2,SLC7A8	4.02E-02	1.23E-01
GO:0030317	sperm motility	4	PLTP,ROPN1B,DNAH11,WT1	6.63E-03	4.06E-02	3	NEURL1,DNAH5,CCDC40	1.90E-02	1.11E-01
GO:0007411	axon guidance	22	DLX5,EPHB6,JAK3,CD72,GFRA3,R	1.65E-02	7.91E-02	18	ROBO2,PLA2G10,FGF10,FLRT3,SF	3.78E-02	1.22E-01
GO:0031069	hair follicle morphogenesis	2	WNT10A,KRT17	4.21E-02	1.11E-01	2	FGF10,BCL2	2.84E-02	1.15E-01
GO:0001654	eye development	2	FOXC1,SMOC1	2.82E-02	1.04E-01	2	BMPR1B,SALL2	1.88E-02	1.11E-01
GO:0001823	mesonephros development	2	BMP7,WT1	3.00E-03	2.46E-02	2	FGF10,GATA3	1.93E-03	5.29E-02
GO:0015701	bicarbonate transport	3	SLC26A9,SLC4A11,CA9	2.08E-02	9.35E-02	3	CA14,PTGER3,CA12	1.25E-02	9.79E-02
GO:0001657	ureteric bud development	3	FOXC1,BMP7,WT1	1.90E-02	8.92E-02	3	ROBO2,RET,NPNT	1.13E-02	9.27E-02
GO:0001895	retina homeostasis	3	CDH3,LYZ,PIGR	1.15E-02	6.11E-02	3	PIP,AZGP1,ZG16B	6.78E-03	7.63E-02
GO:0001656	metanephros development	3	BMP7,WT1,HOXC11	9.19E-03	5.15E-02	3	ROBO2,FGF10,BCL2	5.37E-03	6.90E-02
GO:0006813	potassium ion transport	5	KCNG1,KCNN4,KCNK5,KCNJ15,KC	2.28E-02	9.79E-02	5	ATP1A2,KCNJ3,KCNH1,ABCC8,KC	1.15E-02	9.27E-02
GO:0010951	negative regulation of endopeptidase activity	9	A2ML1,PI3,CSTA,CST7,COL4A3,SL	1.94E-03	2.02E-02	9	CST1,CST5,SERPINA6,CST2,SER	6.06E-04	3.16E-02
GO:0007416	synapse assembly	3	ACHE,DNER,SPOCK2	4.72E-02	1.16E-01	4	GPM6A,FLRT3,SLITRK6,PCLO	6.09E-03	7.11E-02
GO:0007200	phospholipase C-activating G-protein coupled receptor signaling p	3	P2RY6,CRHR1,OPRK1	3.87E-02	1.11E-01	4	AGTR1,GPR139,PTGER3,ESR1	4.64E-03	6.65E-02
GO:0007631	feeding behavior	2	GAL,CRHR1	4.21E-02	1.11E-01	3	PYY,NPY5R,NPY1R	4.16E-03	6.40E-02
GO:0007586	digestion	4	SLC15A1,AMY1A,HTR3A,CYP39A1	1.15E-02	6.11E-02	5	PYY,CAPN9,TFF1,TFF3,CAPN8	1.05E-03	3.67E-02
GO:0007218	neuropeptide signaling pathway	7	GAL,PNOC,NXPH4,CRHR1,OPRK1	1.34E-03	1.57E-02	9	CARTPT,GLRA3,PYY,NPY5R,NXPH	1.42E-05	2.94E-03
GO:0007010	cytoskeleton organization					5	PYY,FGD3,ABLIM3,PCLO,MAST4	4.82E-02	1.27E-01
GO:0016049	cell growth					3	AR,BCL2,XBP1	4.45E-02	1.26E-01
GO:0007626	locomotory behavior					4	NOVA1,NPY1R,ABAT,CELSR1	4.44E-02	1.26E-01
GO:0009791	post-embryonic development					4	MYT1,SCUBE1,BCL2,GATA3	4.44E-02	1.26E-01
GO:0030032	lamellipodium assembly					2	ABLIM3,VAV3	4.34E-02	1.24E-01
GO:0006665	sphingolipid metabolic process					4	ACER2,DEGS2,UGCG,ARSG	4.24E-02	1.23E-01
GO:0009636	response to toxic substance					4	DHRS2,SLC6A4,SLC7A8,BCL2	4.24E-02	1.23E-01
GO:0008104	protein localization					3	WNK4,NBEA,KIF5C	4.21E-02	1.23E-01
GO:0009953	dorsal/ventral pattern formation					2	BMPR1B,LMX1B	4.02E-02	1.23E-01
GO:0019933	cAMP-mediated signaling					2	PCLO,ADCY1	4.02E-02	1.23E-01
GO:0030890	positive regulation of B cell proliferation					2	VAV3,BCL2	4.02E-02	1.23E-01
GO:0072593	reactive oxygen species metabolic process					2	PMAIP1,BCL2	4.02E-02	1.23E-01
GO:0006836	neurotransmitter transport					2	SLC6A4,RIMS4	3.70E-02	1.21E-01
GO:0007017	microtubule-based process					2	TUBA3C,TUBA3E	3.70E-02	1.21E-01
GO:0007528	neuromuscular junction development					2	COL4A5,CACNA2D2	3.70E-02	1.21E-01
GO:0030073	insulin secretion					2	SLC30A8,PCLO	3.70E-02	1.21E-01
GO:0007338	single fertilization					3	CLGN,DNALI1,AR	3.53E-02	1.21E-01
GO:0023014	signal transduction by protein phosphorylation					3	BMPR1B,KCNH1,ERBB4	3.32E-02	1.19E-01
GO:0008344	adult locomotory behavior					3	ATP1A2,SEZ6L,SLITRK6	3.11E-02	1.15E-01
GO:0007616	long-term memory					2	GRIA1,ADCY1	2.58E-02	1.11E-01
GO:1900740	positive regulation of protein insertion into mitochondrial membra					2	PMAIP1,BCL2	2.58E-02	1.11E-01
GO:0030900	forebrain development					3	DCLK1,LRP2,FGFR3	2.55E-02	1.11E-01
GO:0001755	neural crest cell migration					3	RET,SEMA3B,ERBB4	2.37E-02	1.11E-01
GO:0006633	fatty acid biosynthetic process					3	PRKAA2,ELOVL5,XBP1	2.37E-02	1.11E-01
GO:0071277	cellular response to calcium ion					3	SYT1,KCNH1,ADCY1	2.37E-02	1.11E-01
GO:0072332	intrinsic apoptotic signaling pathway by p53 class mediator					2	ZNF385B,PMAIP1	2.34E-02	1.11E-01
GO:0002931	response to ischemia					2	KCNJ11,BCL2	2.10E-02	1.11E-01
GO:0007368	determination of left/right symmetry					3	FGF10,PCSK6,DNAH5	1.90E-02	1.11E-01
GO:0030155	regulation of cell adhesion					3	LAMA3,RET,PRLR	1.90E-02	1.11E-01
GO:0019915	lipid storage					2	PLIN5,NRIP1	1.88E-02	1.11E-01
GO:0030511	positive regulation of transforming growth factor beta receptor sig					2	LRG1,NPNT	1.88E-02	1.11E-01
GO:0009083	branched-chain amino acid catabolic process					2	MCCC2,ACADSB	1.68E-02	1.11E-01
GO:0007566	embryo implantation					3	NLRP5,PRLR,STC2	1.49E-02	1.04E-01
GO:0001836	release of cytochrome c from mitochondria					2	PMAIP1,BCL2	1.48E-02	1.04E-01
GO:0009880	embryonic pattern specification					2	FGF10,ERBB4	1.48E-02	1.04E-01
GO:0010165	response to X-ray					2	PMAIP1,CCND1	1.48E-02	1.04E-01
GO:0071353	cellular response to interleukin-4					2	GATA3,XBP1	1.48E-02	1.04E-01
GO:0006699	bile acid biosynthetic process					2	ACOX2,SLC27A2	1.30E-02	9.79E-02
GO:0010719	negative regulation of epithelial to mesenchymal transition					2	HPN,FOXA1	1.30E-02	9.79E-02
GO:2000378	negative regulation of reactive oxygen species metabolic process					2	PLIN5,BCL2	1.30E-02	9.79E-02
GO:0008202	steroid metabolic process					4	CYP2A6,UGT2B15,LRP2,CYP2B6	1.23E-02	9.79E-02
GO:0001502	cartilage condensation					2	BMPR1B,CHAD	1.14E-02	9.27E-02
GO:0090102	cochlea development					2	SLITRK6,FGFR3	1.14E-02	9.27E-02
GO:0009566	fertilization					3	NLRP5,SPTBN4,SPEF2	1.13E-02	9.27E-02
GO:0001676	long-chain fatty acid metabolic process					2	SLC27A2,ACOT4	9.85E-03	8.74E-02
GO:0007520	myoblast fusion					2	KCNH1,CDON	9.85E-03	8.74E-02
GO:0071577	zinc II ion transmembrane transport					2	SLC30A8,SLC39A6	9.85E-03	8.74E-02
GO:0002028	regulation of sodium ion transport					2	SPTBN4,NKAIN1	8.44E-03	7.82E-02
GO:0006165	nucleoside diphosphate phosphorylation					2	AK8,NME5	8.44E-03	7.82E-02
GO:0019226	transmission of nerve impulse					2	CACNG4,SPTBN4	8.44E-03	7.82E-02
GO:0071310	cellular response to organic substance					2	ABCC8,BCL2	8.44E-03	7.82E-02
GO:2001240	negative regulation of extrinsic apoptotic signaling pathway in abs					3	FGF10,KIAA1324,BCL2	8.43E-03	7.82E-02
GO:0006520	cellular amino acid metabolic process					3	SLC7A4,SLC7A2,SLC7A8	7.58E-03	7.67E-02
GO:0010107	potassium ion import					3	ATP1A2,KCNJ3,KCNJ11	7.58E-03	7.67E-02
GO:0017157	regulation of exocytosis					3	SYT1,PCLO,RASEF	7.58E-03	7.67E-02
GO:0006883	cellular sodium ion homeostasis					2	ATP1A2,SGK3	7.16E-03	7.64E-02
GO:0009813	flavonoid biosynthetic process					2	UGT2B11,UGT2B15	7.16E-03	7.64E-02
GO:0071157	negative regulation of cell cycle arrest					2	FGF10,CCND1	7.16E-03	7.64E-02
GO:0017144	drug metabolic process					3	CYP2A6,CYP2B6,FMO5	6.78E-03	7.63E-02
GO:0006790	sulfur compound metabolic process					2	CHST8,ARSG	6.00E-03	7.11E-02
GO:0008631	intrinsic apoptotic signaling pathway in response to oxidative stres					2	BCL2,MAPT	6.00E-03	7.11E-02
GO:0010259	multicellular organismal aging					2	SLC1A2,SPEF2	6.00E-03	7.11E-02
GO:0010832	negative regulation of myotube differentiation					2	CEACAM5,XBP1	6.00E-03	7.11E-02
GO:0006508	proteolysis					19	PIP,CPB1,MMP10,NRIP3,F7,CAPN	5.17E-03	6.79E-02
GO:0001678	cellular glucose homeostasis					2	CARTPT,MYT1	4.96E-03	6.65E-02
GO:0003351	epithelial cilium movement					2	NME5,CCDC40	4.96E-03	6.65E-02
GO:0010744	positive regulation of macrophage derived foam cell differentiatio					2	PLA2G10,AGTR1	4.96E-03	6.65E-02
GO:2000811	negative regulation of anoikis					2	CEACAM5,BCL2	4.96E-03	6.65E-02
GO:0086010	membrane depolarization during action potential					3	SCN7A,CACNA1F,CACNA1D	4.74E-03	6.65E-02
GO:0014047	glutamate secretion					3	SLC1A1,SYT1,SLC1A2	4.16E-03	6.40E-02

GO:BP Terms ID	Terms	up Basal vs. Luminal N	Genes	p-value	q-value	down Basal vs. Luminal N	Genes	p-value	q-value
GO:0007215	glutamate receptor signaling pathway					2	GRIK4,GRIK3	4.04E-03	6.40E-02
GO:1901381	positive regulation of potassium ion transmembrane transport					2	KCNC2,NOS1AP	4.04E-03	6.40E-02
GO:1902043	positive regulation of extrinsic apoptotic signaling pathway via dea					2	BMPR1B,PMAIP1	4.04E-03	6.40E-02
GO:0030334	regulation of cell migration					5	MMP10,LAMA3,SGK3,ERBB4,PARD	3.27E-03	5.73E-02
GO:0006882	cellular zinc ion homeostasis					2	SLC30A8,SLC39A6	3.23E-03	5.73E-02
GO:0089711	L-glutamate transmembrane transport					2	SLC1A1,SLC1A2	3.23E-03	5.73E-02
GO:2000179	positive regulation of neural precursor cell proliferation					2	ASCL1,CDON	3.23E-03	5.73E-02
GO:0014065	phosphatidylinositol 3-kinase signaling					3	GATA3,IGF1R,XBP1	3.16E-03	5.73E-02
GO:0007269	neurotransmitter secretion					5	SLC1A1,GRM4,SYT1,SLC1A2,ABAT	3.01E-03	5.73E-02
GO:0071805	potassium ion transmembrane transport					7	KCNC2,KCNE4,KCNH1,ABCC8,KCI	2.71E-03	5.69E-02
GO:0030166	proteoglycan biosynthetic process					2	CHST8,BMPR1B	2.53E-03	5.50E-02
GO:0030277	maintenance of gastrointestinal epithelium					2	TFF1,SERPINA3	2.53E-03	5.50E-02
GO:0007595	lactation					4	NEURL1,CCND1,PRLR,ERBB4	2.50E-03	5.50E-02
GO:0006112	energy reserve metabolic process					7	KCNC2,PRKAA2,CACNA2D2,ABCC	2.39E-03	5.50E-02
GO:0002088	lens development in camera-type eye					3	SLITRK6,CDON,GATA3	2.33E-03	5.50E-02
GO:0006805	xenobiotic metabolic process					10	UGT2B11,CYP2A6,CYP2A7,CYP4F	2.07E-03	5.44E-02
GO:0009100	glycoprotein metabolic process					2	ST8SIA6,PCSK6	1.93E-03	5.29E-02
GO:0002675	positive regulation of acute inflammatory response					2	NPY5R,IL6ST	1.43E-03	4.29E-02
GO:0006636	unsaturated fatty acid biosynthetic process					2	ELOVL2,ELOVL5	1.43E-03	4.29E-02
GO:0071392	cellular response to estradiol stimulus					3	ZNF703,NRIP1,ESR1	1.39E-03	4.29E-02
GO:0007628	adult walking behavior					4	TRH,SPTBN4,GLRB,MAPT	1.03E-03	3.67E-02
GO:0007156	homophilic cell adhesion via plasma membrane adhesion molecu					10	ROBO2,PCDHA12,CDHR3,IGSF9B,	9.62E-04	3.67E-02
GO:0007409	axonogenesis					7	CACNA1F,SLITRK6,SPTBN4,ADCY	7.52E-04	3.16E-02
GO:0021591	ventricular system development					3	AK8,CELSR2,NME5	7.51E-04	3.16E-02
GO:0031017	exocrine pancreas development					2	IGF1R,XBP1	6.92E-04	3.16E-02
GO:0001764	neuron migration					8	ASCL1,GPM6A,DCLK1,DCDC2,CEL	6.46E-04	3.16E-02
GO:0019373	epoxygenase P450 pathway					3	CYP2A6,CYP2A7,CYP2B6	5.95E-04	3.16E-02
GO:0001964	startle response					3	SLITRK6,KCNH1,GLRB	4.64E-04	3.16E-02
GO:0016079	synaptic vesicle exocytosis					6	SYT1,SYT13,PCLO,SYTL5,SYT9,SY	1.96E-04	1.55E-02
GO:0006906	vesicle fusion					6	SYT1,SYT13,VAV3,SYTL5,SYT9,SY	1.33E-04	1.20E-02
GO:0017158	regulation of calcium ion-dependent exocytosis					5	SYT1,SYT13,SYTL5,SYT9,SYTL4	1.14E-04	1.20E-02
GO:0001580	detection of chemical stimulus involved in sensory perception of b					4	CST1,PIP,CST2,AZGP1	2.66E-05	3.35E-03
GO:0010039	response to iron ion					4	TFF1,CCND1,BCL2,ABAT	1.86E-05	2.94E-03
GO:0007268	synaptic transmission					25	GRIA1,CARTPT,SLC1A1,GRM4,SYT	1.70E-06	5.37E-04
GO:0003341	cilium movement					7	SPAG17,CFAP221,DNAH5,DNAH7,I	1.53E-08	9.63E-06

Supplementary Table 7: List of 89 proteins/phosphoproteins differentially expressed between the ER+/HER2- Luminal and Basal breast cancers (TCGA)

Gene	Luminal vs. Basal				
	Protein	N	Odds ratio [95%CI]	p-value[1]	q-value
ESR1	ER-alpha	510	4.30 [3.30-5.58]	1.50E-18	1.18E-16
PGR	PR	510	3.45 [2.54-4.70]	9.51E-11	8.31E-10
GATA3	GATA3	510	2.32 [1.94-2.79]	1.40E-13	3.67E-12
INPP4B	INPP4B	510	1.87 [1.61-2.16]	6.60E-12	6.49E-11
AR	AR	510	1.81 [1.59-2.07]	3.07E-13	4.83E-12
PREX1	PREX1	510	1.77 [1.53-2.04]	1.16E-10	9.12E-10
BCL2	Bcl-2	510	1.75 [1.55-1.99]	4.76E-13	6.24E-12
ESR1	ER-alpha_pS118	510	1.65 [1.48-1.85]	2.93E-13	4.83E-12
FASN	FASN	510	1.54 [1.26-1.87]	3.49E-04	0.000741893
KDR	VEGFR2	510	1.47 [1.32-1.64]	2.35E-08	1.68E-07
GUSP4	DUSP4	510	1.41 [1.20-1.64]	3.60E-04	0.000745137
ERBB2	HER2	510	1.39 [1.26-1.54]	7.96E-08	5.22E-07
ACACA ACACB	ACC_pS79	510	1.34 [1.18-1.52]	1.18E-04	0.000290034
CLDN7	Claudin-7	510	1.30 [1.12-1.51]	4.37E-03	0.006365097
GSK3A GSK3B	GSK3-alpha-beta_pS21_S9	510	1.25 [1.11-1.40]	1.93E-03	0.003300022
TP53BP1	53BP1	510	1.24 [1.12-1.37]	5.02E-04	0.00101241
PARK7	DJ-1	510	1.23 [1.15-1.32]	1.58E-06	7.77E-06
ACACA	ACC1	510	1.19 [1.03-1.37]	4.31E-02	0.040356671
GSK3A GSK3B	GSK3_pS9	510	1.19 [1.05-1.33]	1.78E-02	0.019940292
EEF2K	eEF2K	510	1.19 [1.09-1.29]	1.39E-03	0.00259739
PRKAA1	AMPK_alpha	510	1.18 [1.11-1.25]	8.73E-06	3.27E-05
BCL2L11	Bim	510	1.18 [1.07-1.30]	5.86E-03	0.008086119
ERBB3	HER3	510	1.18 [1.07-1.29]	4.08E-03	0.00629227
MAPK9	JNK2	510	1.18 [1.11-1.26]	2.86E-05	9.22E-05
BRAF	B-Raf	510	1.17 [1.04-1.32]	2.62E-02	0.028229019
RPTOR	Raptor	510	1.17 [1.11-1.23]	1.42E-06	7.45E-06
RB1	Rb_pS807_S811	510	1.17 [1.05-1.30]	1.66E-02	0.018922406
MTOR	mTOR	510	1.17 [1.07-1.28]	2.77E-03	0.004538955
COG3	COG3	510	1.16 [1.05-1.27]	1.47E-02	0.017256785
IRS1	IRS1	510	1.16 [1.09-1.23]	6.33E-05	0.000171681
MAPK8	JNK_pT183_pY185	510	1.14 [1.06-1.22]	4.54E-03	0.006376541
PDK1	PDK1_pS241	510	1.13 [1.06-1.21]	1.85E-03	0.003233528
ERCC5	ERCC5	510	1.12 [1.04-1.20]	1.52E-02	0.017581342
ERBB2	HER2_pY1248	510	1.12 [1.06-1.19]	1.63E-03	0.00291375
DIABLO	Smac	510	1.12 [1.02-1.22]	4.12E-02	0.040006407
CDKN1B	p27	510	1.12 [1.05-1.20]	6.11E-03	0.008285726
SMAD3	Smad3	510	1.11 [1.06-1.16]	2.38E-04	0.000519986
RICTOR	Rictor_pT1135	510	1.09 [1.03-1.15]	1.28E-02	0.015730675
TSC2	Tuberin_pT1462	510	1.08 [1.01-1.16]	4.41E-02	0.040515633
DPP4	CD26	510	1.07 [1.01-1.13]	4.43E-02	0.040515633
BIRC2	cIAP	510	1.07 [1.02-1.12]	3.14E-02	0.032929545
IRF1	IRF-1	510	1.06 [1.01-1.11]	4.67E-02	0.042219684
RAD50	Rad50	510	1.06 [1.01-1.12]	4.97E-02	0.043922164
FOXO3	FOXO3a_pS318_S321	510	0.96 [0.93-0.99]	3.91E-02	0.039427524
CHEK1	Chk1_pS345	510	0.94 [0.90-0.99]	4.19E-02	0.040179554
PIK3CA	PI3K-p110-alpha	510	0.94 [0.89-0.98]	2.23E-02	0.024360697
SMAD1	Smad1	510	0.94 [0.90-0.98]	8.98E-03	0.011578808
CCNE2	Cyclin_E2	510	0.93 [0.88-0.98]	3.45E-02	0.035704492
NRG1	Heregulin	510	0.93 [0.89-0.97]	6.83E-03	0.009105128
PCNA	PCNA	510	0.93 [0.88-0.98]	2.74E-02	0.029123006
SRC	Src	510	0.93 [0.88-0.97]	1.44E-02	0.017160736
AXL	Axl	431	0.92 [0.86-0.98]	4.08E-02	0.040006407
CHEK1	Chk1	510	0.92 [0.88-0.97]	4.36E-03	0.006365097
CDH2	N-Cadherin	510	0.92 [0.87-0.98]	4.12E-02	0.040006407
EIF4EBP1	4E-BP1_pT70	510	0.91 [0.86-0.96]	3.00E-03	0.004719202
PEA15	PEA15_pS116	510	0.91 [0.85-0.98]	3.60E-02	0.036773006
SRC	Src_pY416	510	0.91 [0.84-0.98]	4.83E-02	0.043169976
TGM2	Transglutaminase	510	0.91 [0.87-0.96]	4.37E-03	0.006365097
G6PD	G6PD	510	0.90 [0.84-0.96]	9.58E-03	0.012153215
WWTR1	TAZ	510	0.90 [0.85-0.95]	2.92E-03	0.004687099
CHEK2	Chk2_pT68	510	0.89 [0.82-0.98]	4.24E-02	0.040179554
NOTCH1	Notch1	510	0.89 [0.85-0.93]	4.95E-05	0.000144198
EEF2	eEF2	510	0.89 [0.83-0.95]	4.46E-03	0.006376541
PRKCA	PKC-alpha	510	0.88 [0.83-0.92]	1.44E-05	5.15E-05
EIF4EBP1	4E-BP1	510	0.86 [0.77-0.95]	1.80E-02	0.019940292
EIF4EBP1	4E-BP1_pS65	510	0.86 [0.82-0.91]	6.06E-06	2.51E-05
MAP2K1	MEK1	510	0.86 [0.80-0.92]	2.34E-04	0.000519986
TSC1	TSC1	510	0.86 [0.77-0.95]	1.22E-02	0.015231288
EGFR	EGFR	510	0.85 [0.80-0.90]	1.33E-06	7.45E-06
PRKCA	PKC-alpha_pS657	510	0.85 [0.80-0.91]	7.71E-05	0.000202139
CDH3	P-Cadherin	510	0.84 [0.79-0.89]	1.16E-06	7.02E-06
MSH6	MSH6	510	0.83 [0.76-0.91]	1.16E-03	0.002225315
EIF4EBP1	4E-BP1_pT37_T46	510	0.82 [0.73-0.93]	7.76E-03	0.010172503
CCNE1	Cyclin_E1	510	0.82 [0.76-0.88]	5.31E-06	2.32E-05
SERPINE1	PAI-1	510	0.82 [0.74-0.91]	1.99E-03	0.003330217
TP53	p53	510	0.81 [0.73-0.89]	2.16E-04	0.00049968
FOXM1	FoxM1	510	0.80 [0.73-0.87]	2.20E-05	7.52E-05
TFRC	TFRC	510	0.79 [0.69-0.89]	1.42E-03	0.00259739
LCK	Lck	510	0.77 [0.71-0.84]	1.93E-06	8.93E-06
ANXA1	Annexin-1	510	0.76 [0.68-0.85]	4.13E-05	0.000124938
RPS6	S6_pS240_S244	510	0.75 [0.66-0.84]	6.20E-05	0.000171681
RPS6	S6_pS235_S236	510	0.74 [0.65-0.84]	1.74E-04	0.000414718
SYK	Syk	510	0.74 [0.66-0.82]	7.32E-06	2.88E-05
NDRG1	NDRG1_pT346	510	0.73 [0.64-0.83]	1.13E-04	0.000286704
PARP1	PARP1	253	0.73 [0.59-0.90]	1.34E-02	0.016214695
GAPDH	GAPDH	510	0.69 [0.58-0.83]	7.66E-04	0.001506212
ASNS	ASNS	510	0.68 [0.62-0.74]	1.13E-13	3.67E-12
CCNB1	Cyclin_B1	510	0.67 [0.57-0.78]	2.93E-05	9.22E-05
CASP7	Caspase-7_cleavedD198	510	0.64 [0.58-0.71]	3.34E-12	3.75E-11

[1], logistic regression

Supplementary Table 8: List of breast cancer data sets included in the study

Reference	Source of data	N° of samples	Technological platform	N° of probe sets	N° of samples used
1	http://microarray-pubs.stanford.edu/wound_NKI/	295	Agilent Hu25K	25K	254
2	http://www.rii.com/publications/2002/vantveer.html	117	Agilent Hu25K	25K	117
3	https://expo.intgen.org/geo GEO: GSE2109	348	Affymetrix U133 Plus 2.0	54K	348
4	GEO: GSE1561	49	Affymetrix U133A	22K	49
5	GEO: GSE2603	99	Affymetrix U133A	22K	99
6	GEO: GSE2034	286	Affymetrix U133A	22K	286
7	MDA133	133	Affymetrix U133A	22K	131
8	GEO: GSE4922, GSE1456	448	Affymetrix U133 A+B	2x22K	448
9	GEO: GSE2990	189	Affymetrix U133A	22K	80
10	GEO: GSE6861, GSE4779	161	Affymetrix X3P	61K	125
11	GEO: GSE7390	198	Affymetrix U133A	22K	154
12	GEO: GSE6596	26	Affymetrix U133A	22K	24
13	GEO: GSE13787	23	Affymetrix U133 Plus 2.0	54K	23
14	Array Express: E-TABM-158	130	Affymetrix U133AAofAv2	23K	130
15	GEO: GSE11121	200	Affymetrix U133A	22K	200
16	GEO: GSE5364	196	Affymetrix U133A	22K	183
17	GEO: GSE12276	204	Affymetrix U133 Plus 2.0	54K	204
18	GEO: GSE12763	30	Affymetrix U133 Plus 2.0	54K	30
19	GEO: GSE12093	136	Affymetrix U133A	22K	136
20	GEO: GSE5462	116	Affymetrix U133A	22K	116
21	GEO: GSE23593	50	Affymetrix U133 Plus 2.0	54K	50
22	GEO: GSE18728	61	Affymetrix U133 Plus 2.0	54K	61
23	GEO: GSE18229	337	Agilent Hu25K	25K	264
24	GEO: GSE18864	84	Affymetrix U133 Plus 2.0	54K	84
25	GEO: GSE20271	178	Affymetrix U133A	22K	178
26	GEO: GSE22133	359	Swegene H_v2.1.1 55K	55K	346

27	GEO: GSE10780	185	Affymetrix U133 Plus 2.0	54K	42
28	GEO: GSE20194	278	Affymetrix U133A	22K	91
29	GEO: GSE22093, GSE22597	247	Affymetrix U133A	22K	100
30	GEO: GSE16446	120	Affymetrix U133 Plus 2.0	54K	120
31	Array Express: E-MTAB-365	537	Affymetrix U133 Plus 2.0	54K	452
32	GEO: GSE25066	508	Affymetrix U133A	22K	504
33	GEO: GSE31448	353	Affymetrix U133 Plus 2.0	54K	286
34	TCGA Data Portal - BRCA -	1215	Illumina, RNAseq V2	20K	1092
35	GEO: GSE29442, GSE35186	201	Agilent-014850 4x44K	44K	201
36	EGA: EGAS00000000083	2136	Illumina HT 12	49K	1974
	TOTAL	10233			8982

1 van de Vijver, M. J. *et al.* A gene-expression signature as a predictor of survival in breast cancer. *N Engl J Med* **347**, 1999-2009. (2002).

2 van 't Veer, L. J. *et al.* Gene expression profiling predicts clinical outcome of breast cancer. *Nature* **415**, 530-536 (2002).

3 Curley, E. Expression Project for Oncology (expO). *Unpublished* http://www.intgen.org/research-services/biobanking-experience/expo/ (2005).

4 Farmer, H. *et al.* Targeting the DNA repair defect in BRCA mutant cells as a therapeutic strategy. *Nature* **434**, 917-921 (2005).

5 Minn, A. J. *et al.* Genes that mediate breast cancer metastasis to lung. *Nature* **436**, 518-524, doi:10.1038/nature03799 (2005).

6 Wang, Y. *et al.* Gene-expression profiles to predict distant metastasis of lymph-node-negative primary breast cancer. *Lancet* **365**, 671-679. (2005).

7 Hess, K. R. *et al.* Pharmacogenomic predictor of sensitivity to preoperative chemotherapy with paclitaxel and fluorouracil, doxorubicin, and cyclophosphamide in breast cancer. *J Clin Oncol* **24**, 4236-4244 (2006).

8 Ivshina, A. V. *et al.* Genetic reclassification of histologic grade delineates new clinical subtypes of breast cancer. *Cancer Res* **66**, 10292-10301 (2006).

9 Sotiriou, C. *et al.* Gene expression profiling in breast cancer: understanding the molecular basis of histologic grade to improve prognosis. *J Natl Cancer Inst* **98**, 262-272 (2006).

10 Bonnefoi, H. *et al.* Validation of gene signatures that predict the response of breast cancer to neoadjuvant chemotherapy: a substudy of the EORTC 10994/BIG 00-01 clinical trial. *Lancet Oncol* **8**, 1071-1078 (2007).

11 Desmedt, C. *et al.* Strong time dependence of the 76-gene prognostic signature for node-negative breast cancer patients in the TRANSBIG multicenter independent validation series. *Clin Cancer Res* **13**, 3207-3214 (2007).

12 Klein, A. *et al.* Comparison of gene expression data from human and mouse breast cancers: identification of a conserved breast tumor gene set. *Int J Cancer* **121**, 683-688, doi:10.1002/ijc.22630 (2007).

13 Marty, B. *et al.* Frequent PTEN genomic alterations and activated phosphatidylinositol 3-kinase pathway in basal-like breast cancer cells. *Breast Cancer Res* **10**, R101, doi:10.1186/bcr2204 (2008).

14 Chin, K. *et al.* Genomic and transcriptional aberrations linked to breast cancer pathophysiologies. *Cancer Cell* **10**, 529-541 (2006).

15 Schmidt, M. *et al.* The humoral immune system has a key prognostic impact in node-negative breast cancer. *Cancer Res* **68**, 5405-5413 (2008).

16 Yu, K. *et al.* A precisely regulated gene expression cassette potently modulates metastasis and survival in multiple solid cancers. *PLoS Genet* **4**, e1000129, doi:10.1371/journal.pgen.1000129 (2008).

17 Bos, P. D. *et al.* Genes that mediate breast cancer metastasis to the brain. *Nature* **459**, 1005-1009, doi:10.1038/nature08021 (2009).

18 Hoeflich, K. P. *et al.* In vivo antitumor activity of MEK and phosphatidylinositol 3-kinase inhibitors in basal-like breast cancer models. *Clin Cancer Res* **15**, 4649-4664, doi:10.1158/1078-0432.CCR-09-0317 (2009).

19 Zhang, Y. *et al.* The 76-gene signature defines high-risk patients that benefit from adjuvant tamoxifen therapy. *Breast Cancer Res Treat* **116**, 303-309, doi:10.1007/s10549-008-0183-2 (2009).

20 Miller, W. R. & Larionov, A. Changes in expression of oestrogen regulated and proliferation genes with neoadjuvant treatment highlight heterogeneity of clinical resistance to the aromatase inhibitor, letrozole. *Breast Cancer Res* **12**, R52, doi:10.1186/bcr2611 (2010).

21 Barry, W. T. *et al.* Intratumor heterogeneity and precision of microarray-based predictors of breast cancer biology and clinical outcome. *J Clin Oncol* **28**, 2198-2206, doi:10.1200/JCO.2009.26.7245 (2010).

22 Korde, L. A. *et al.* Gene expression pathway analysis to predict response to neoadjuvant docetaxel and capecitabine for breast cancer. *Breast Cancer Res Treat* **119**, 685-699, doi:10.1007/s10549-009-0651-3 (2010).

23 Prat, A. *et al.* Phenotypic and molecular characterization of the claudin-low intrinsic subtype of breast cancer. *Breast Cancer Res* **12**, R68 (2010).

24 Silver, D. P. *et al.* Efficacy of neoadjuvant Cisplatin in triple-negative breast cancer. *J Clin Oncol* **28**, 1145-1153 (2010).

25 Tabchy, A. *et al.* Evaluation of a 30-gene paclitaxel, fluorouracil, doxorubicin, and cyclophosphamide chemotherapy response predictor in a multicenter randomized trial in breast cancer. *Clin Cancer Res* **16**, 5351-5361 (2010).

26 Jonsson, G. *et al.* Genomic subtypes of breast cancer identified by array-comparative genomic hybridization display distinct molecular and clinical characteristics. *Breast Cancer Res* **12**, R42, doi:10.1186/bcr2596 (2010).

27 Chen, D. T. *et al.* Proliferative genes dominate malignancy-risk gene signature in histologically-normal breast tissue. *Breast Cancer Res Treat* **119**, 335-346, doi:10.1007/s10549-009-0344-y (2010).

28 Popovici, V. *et al.* Effect of training-sample size and classification difficulty on the accuracy of genomic predictors. *Breast Cancer Res* **12**, R5, doi:10.1186/bcr2468 (2010).

29 Iwamoto, T. *et al.* Gene pathways associated with prognosis and chemotherapy sensitivity in molecular subtypes of breast cancer. *J Natl Cancer Inst* **103**, 264-272 (2011).

30 Desmedt, C. *et al.* Multifactorial approach to predicting resistance to anthracyclines. *J Clin Oncol* **29**, 1578-1586 (2011).

31 Guedj, M. *et al.* A refined molecular taxonomy of breast cancer. *Oncogene* **31**, 1196-1206 (2012).

32 Hatzis, C. *et al.* A genomic predictor of response and survival following taxane-anthracycline chemotherapy for invasive breast cancer. *JAMA* **305**, 1873-1881 (2011).

33 Sabatier, R. *et al.* Down-regulation of ECRG4, a candidate tumor suppressor gene, in human breast cancer. *PLoS One* **6**, e27656, doi:10.1371/journal.pone.0027656 (2011).

34 Cancer Genome Atlas, N. Comprehensive molecular portraits of human breast tumours. *Nature* **490**, 61-70, doi:10.1038/nature11412 (2012).

35 Ellis, M. J. *et al.* Whole-genome analysis informs breast cancer response to aromatase inhibition. *Nature* **486**, 353-360, doi:10.1038/nature11143 (2012).

36 Curtis, C. *et al.* The genomic and transcriptomic architecture of 2,000 breast tumours reveals novel subgroups. *Nature* **486**, 346-352, doi:10.1038/nature10983 (2012).
